# Synthesis of Fusahexin and Characterization of a Natural β‑Turn Dipeptide

**DOI:** 10.1021/acs.joc.5c01080

**Published:** 2025-07-29

**Authors:** Carmen Bäuerlein, Armin Geyer

**Affiliations:** 54308 Philipps-University Marburg, Department of Chemistry, Hans-Meerwein-Straße 4, 35043 Marburg, Germany

## Abstract

We describe the first synthesis of the natural cyclic hexapeptide Fusahexin *cyclo*(-ala-Leu-ehr=Pro-leu-Leu) (**1**) containing the bicyclic dipeptide ehr=Pro, a tetrahydro-1,3-oxazin-4-one formed from erythronine and proline. Fusahexin and several other peptides were obtained either by using the bicyclic dipeptide Fmoc-ehr=Pro-OH (**2**) in SPPS or by late-stage oxidation of a precursor peptide aldehyde. In all cases studied, the bicyclic dipeptide ehr=Pro cyclizes by acid-promoted condensation, forming the bridge head of the fused bicyclic ring exclusively in *R* configuration. The conformational behavior of Fusahexin and several stereochemical variants was characterized by NMR and modeling. Its convenient accessibility and its transferability make the β-turn dipeptide ehr=Pro a useful general reverse-turn dipeptide.

## Graphical abstract

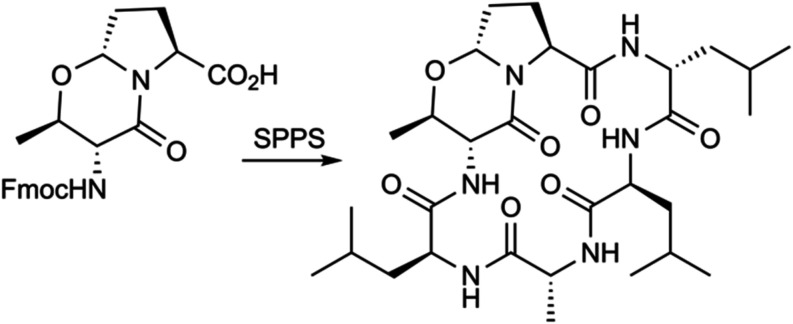

## Introduction

The peptide natural product Fusahexin (**1**) from was described by Westphal et al.^1^ This cyclic hexapeptide is characterized by an alternating sequence of d- and l-configurated amino acids and the 6,5-fused bicyclic dipeptide ehr=Pro. α-Amino acids are labeled here in a three-letter code with the first letter capitalized for l-configuration and small letters for d-amino acids. The double hyphen indicates a bicyclic lactam, which tethers two neighboring amino acid side chains to the backbone. A nomenclature established in literature.^2^ The biosynthesis of Fusahexin (**1**) was proposed by an oxidative functionalization of Pro, followed by the addition of the secondary alcohol. The reduction of Glu to Glu­(δ-aldehyde) followed by *N*-acyliminium cyclization is a plausible biosynthetic alternative. Both pathways were studied by using different amino acids in the chemical synthesis of bicyclic *N*-acyl-*N*,*O*-acetal dipeptides. Either an aldehyde was generated by olefin oxidation^3^ or the pyrrolidine ring was activated by electrochemical oxidation.^4^ Here, we investigate whether d-*allo*-Thr (ehr, erythronine) in a ehr-Glu­(δ-aldehyde) dipeptide form the bridge head under substrate control or whether it requires enzymatic catalysis to obtain the stereocenter under high stereocontrol in a *N*-acyliminium cyclization. Fusahexin (**1**) is not only an interesting natural product but also a role model for the characterization of the β-turn mimic ehr=Pro. We describe the synthesis of Fusahexin and several other peptides by using the bicyclic dipeptide Fmoc-ehr=Pro-OH (**2**) in solid phase peptide synthesis (SPPS) or by the late-stage oxidation of precursor peptides containing the ehr-Hag (l-homoallylglycine) sequence. Our synthetic protocol toward the storable dipeptide Fmoc-ehr=Pro-OH (**2**) or late-stage oxidation of the ehr-Hag precursor, respectively, makes the natural β-turn dipeptide ehr=Pro available for the investigation of its β-turn stabilizing effects in other peptides, too.

## Results and Discussion

### Synthesis

Scheme 1 shows the synthesis of Fusahexin.

**Scheme 1:**
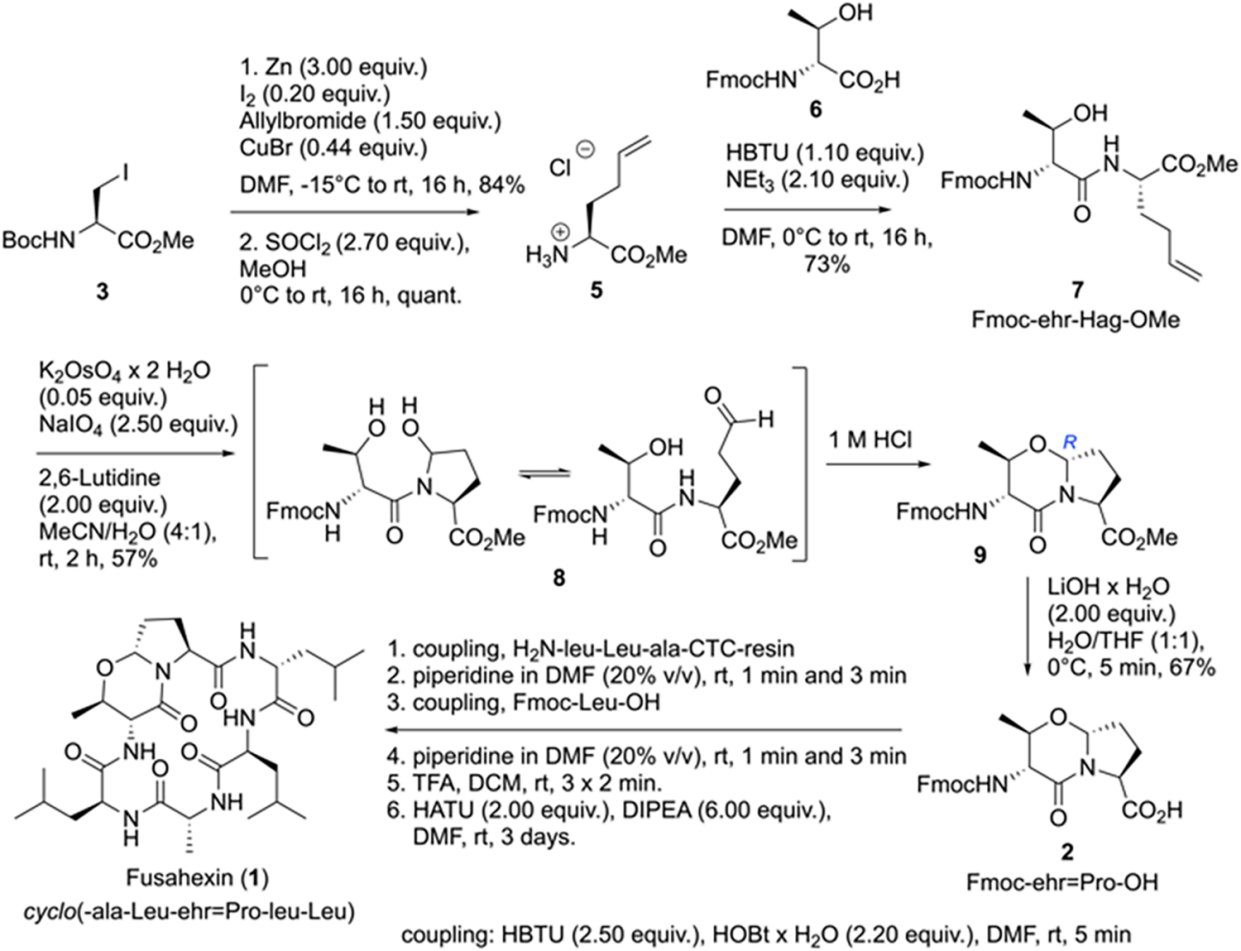
Synthesis of Fusahexin (**1**)

Boc-protected iodoalanine methyl ester (**3**)^5^ was activated by zinc for a Negishi-type allylation with catalytic amounts of copper bromide to obtain Boc-Hag-OMe (**4**) in 84% yield according to an improved protocol^6^ of the original synthesis.^7^ Acid-promoted Boc-fragmentation with thionyl chloride (2.7 equiv) yielded the salt HCl.H-Hag-OMe (**5**), which was subsequently acylated by HBTU-activated Fmoc-ehr-OH (**6**) to obtain dipeptide Fmoc-ehr-Hag-OMe (**7**) in 73% over two steps. The ehr secondary alcohol did not require protection in this condensation step. The Hag terminal olefin was cleaved by osmium tetraoxide and sodium periodate to yield Fmoc-ehr-Glu­(δ-aldehyde)-OMe (**8**) in a single transformation (Lemieux-Johnson reaction).^3^ This aldehyde closes quantitatively to the hemiaminal, showing two signal sets in the ^1^H NMR spectrum from *cis*-*trans* isomerism about the tertiary amide bond. Catalytic amounts of 1 m HCl were required for the acid-promoted dehydration via the N-acyliminium intermediate to obtain Fmoc-ehr=Pro-OMe (**9**) as a single signal set in the ^1^H NMR spectrum from the highly diastereoselective addition of the secondary alcohol of ehr to the iminium ion in the formation of the oxazine ring (Figure 1).^8^ The configuration for the bridge head proton was defined by ROESY contacts between the bridge head, the methyl group, and the Hα of ehr. The intensity of this contact indicates *R*-configuration at the bridge head.

**Figure 1:**
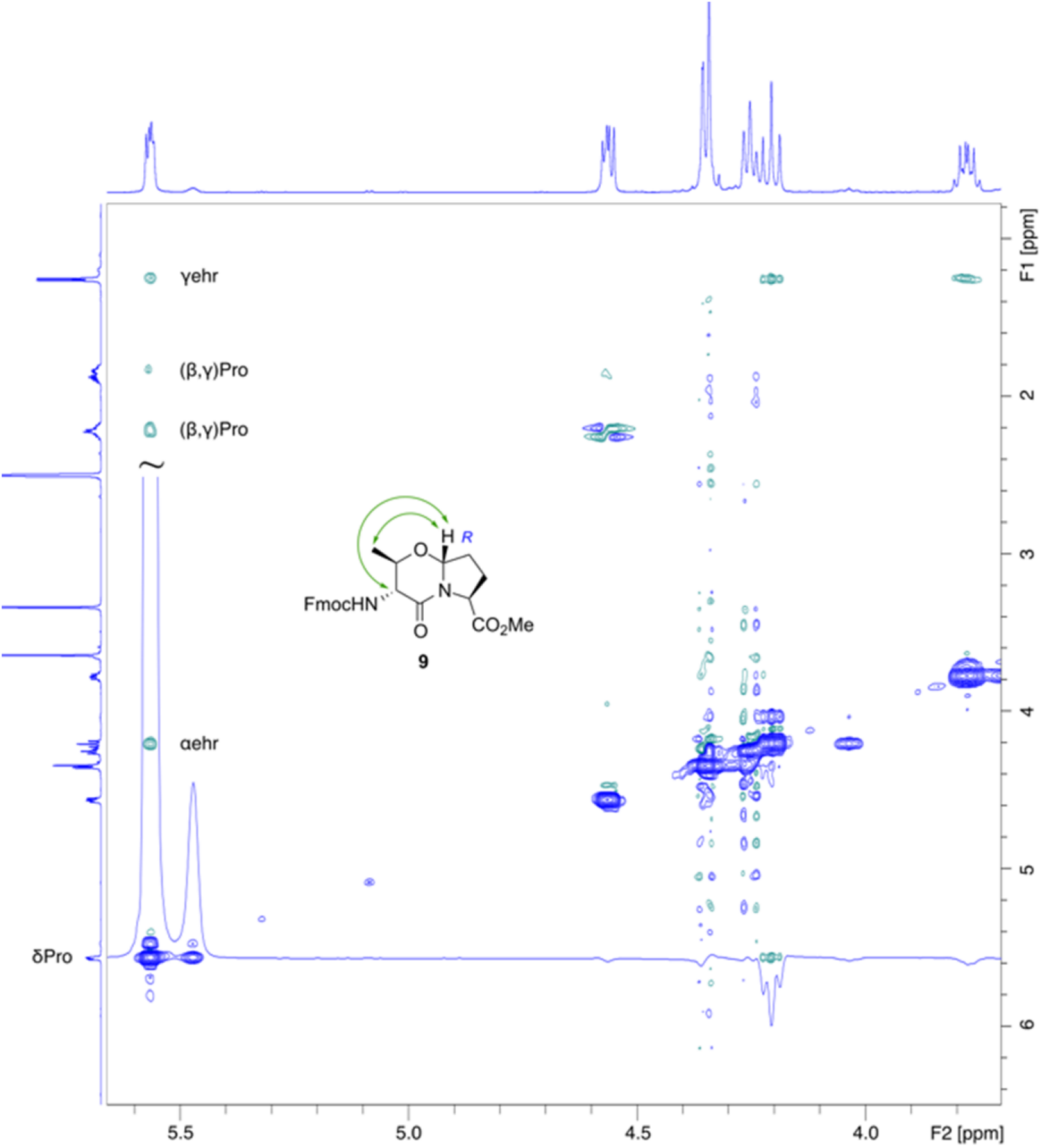
Expansion from the ROESY spectrum of Fmoc-ehr=Pro-OH (**9**). The row shows that the second signal set from the cis-urethane isomer is in slow exchange with the main signal set. The positive cross signal identifies exchange, and the negative is the NOE-contacts, which identify the bridge head proton δPro to be on the same ring side as αehr and γehr (500 MHz, 300 K, DMSO-*d*_6_).

Final saponification of the methyl ester with LiOH (2.00 equiv) under optimized conditions at 0 °C to avoid Fmoc elimination yielded the storable dipeptide Fmoc-ehr=Pro-OH (**2**) for further use in peptide synthesis.

SPPS was carried out on a semiautomatic peptide synthesizer with 5.0 equiv of Fmoc amino acid and DIC/Oxyma activation, except for Fmoc-ehr=Pro-OH (**2**), which was used with only a 2-fold excess to save material. After cleaving the peptide from the resin with 1% TFA in DCM, 3 times for 2 min, the deletion sequences were separated by HPLC. All hexapeptides were synthesized with a C-terminal ala/Ala and cyclized under dilution conditions with the coupling reagent HATU (2.0 equiv) and DIPEA (6.0 equiv) for 72 h at a peptide dilution of 1 mmol/L.^9^ The ehr=Pro dipeptide shows no problems with the isomerization of *N*-acylated *N*,*O*-acetal. The tetrahydro-1,3-oxazin-4-one proved to be stable under peptide synthesis conditions and in all further linear and cyclic peptides studied here. Fusahexin (**1**) was purified by removing the DMF and washing the pale yellow precipitate with water/MeCN (4:1) for 5 min in an ultrasonic bath. After the suspension was centrifuged, the solvent was decanted, and the remaining solid was washed again with water/MeCN (4:1) to yield the pure synthetic Fushexin (**1**) as a white solid. The diastereomeric peptides *cyclo*(-Ala-Leu-ehr=Pro-Leu-Leu) (**10**) and *cyclo*(-Ala-Leu-ehr=Pro-Leu-leu) (**11**) (Table S12) were synthesized accordingly. The cyclic hexapeptides showed different solubilities in aqueous solution. The NMR spectroscopic data then confirmed the individual characteristics of the peptides.

### Conformational Analysis

To a first approximation, cyclic hexapeptides resemble mini-β-sheets assembled from two antiparallel β-hairpin turns.^10^ A d-configurated amino acid acts as a conformational anchor that shifts epitopes to different β-turn positions in a predictable manner.^11^ Proline or *N*-alkylated amino acids are used in a similar way.^12^ Cβ-branched bicyclic dipeptides^13^ can override the directing influence of d-amino acids or that of flexible dipeptides like BTD.^14^ The bicyclic dipeptide ehr=Pro combines both directing elements in a single molecule, the d-configurated backbone, and the Cβ-branching.

Since Fusahexin (**1**) is practically insoluble in water, we studied its conformational behavior in DMSO-*d*_6_ and pyridine-d_5_, respectively, without detecting significant differences in the order of chemical shift resonances, NOE patterns, and temperature dependence of chemical shifts (Δδ/Δ*T*) between the two aprotic solvents. Therefore, only the NMR data detected in DMSO-*d*_6_ are discussed in the following. The ^1^H NMR resonance signals of synthetic Fusahexin resemble the signals observed for the isolated peptide in Lit.^1^ The signal overlap of ala^1^NH and ehr^3^NH around 308 K is resolved at higher temperatures because the Δδ/Δ*T* of ala^1^NH is more positive than that of ehr^3^NH. Figure 2 shows an expansion of the NH/NH region and the ^1^H NMR spectrum between 300 and 330 K. Further information is gained from the ROESY spectrum. Besides the strong leu^5^NH→Leu^6^NH NOE contact, one observes a medium NOE contact leu^5^NH→ Pro^4^αH characteristic for a β-turn around Pro^4^ and leu^5^ in the *i + 1* and *i + 2* positions. The exocyclic ^3^ *J*_NH,α_ coupling of 8.9 Hz points to an extended orientation as expected for ehr=Pro in the *i* to *i + 1* position of a β-turn. The ^3^ *J*_α,β_ coupling constant of the oxazine ring shows values between 8 and 9 Hz from the bisequatorial orientation of the α-amino substituent and the neighboring methyl group. This antiperiplanar coupling is observed for the dipeptide ehr=Pro, for the linear hexapeptides, and for all the cyclic peptides studied here. Further NMR data hint toward fast conformational averaging with temperature dependence of NH chemical shifts varying from 3 to 5 ppb/K and the NH/NH as well as NH/Hα NOE contacts are of similar (time averaged) intensity. An energy-minimized, dominant, but probably not exclusive conformation is shown in Figure 2.

**Figure 2:**
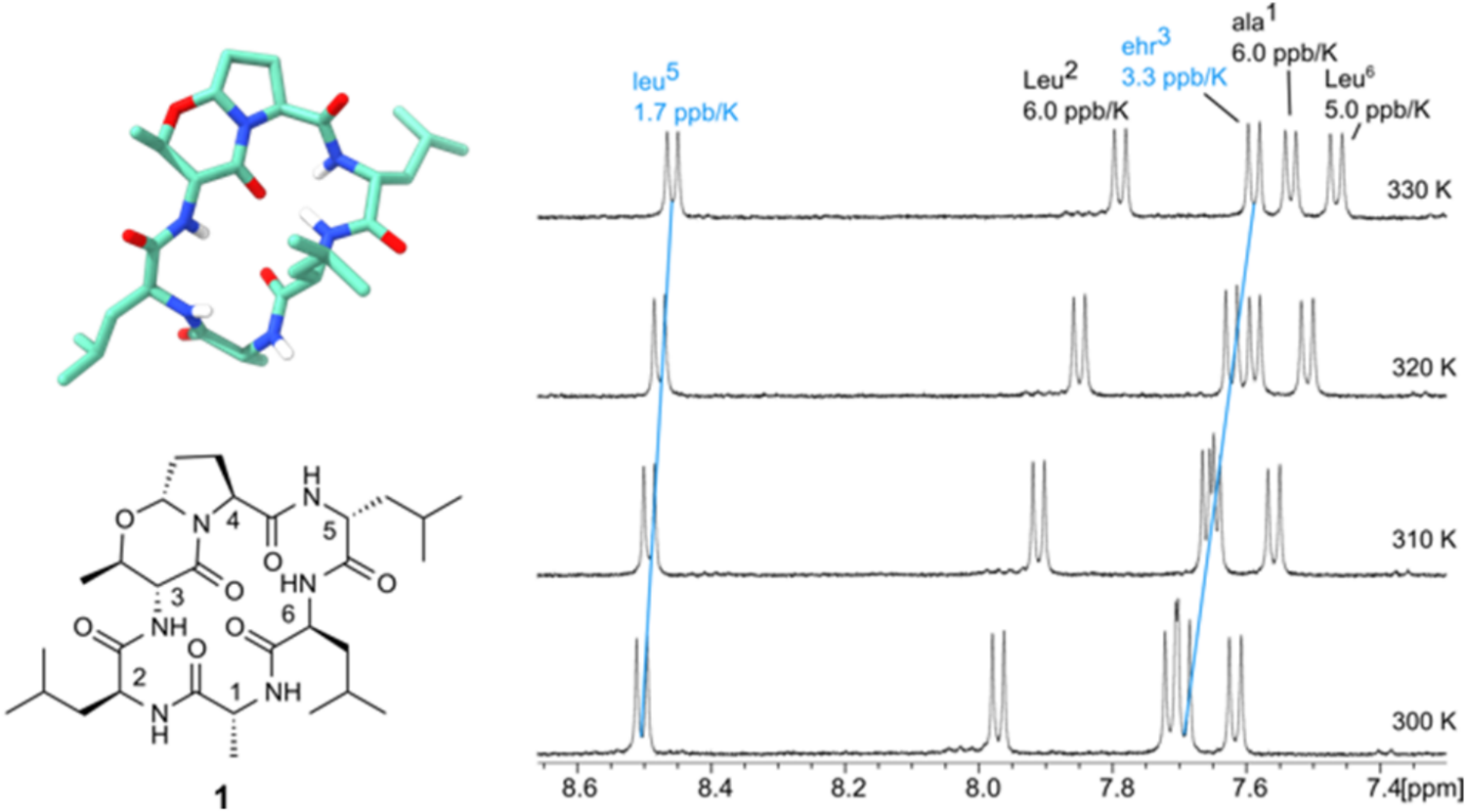
3D structure of Fusahexin (**1**) showing the opposing βI- and βII-turns, which resemble the main conformation in solution. leu^5^NH is significantly deshielded and shows the lowest chemical shift dependence upon variation of the measuring temperature (^1^H NMR spectrum, 500 MHz, 300–330 K, DMSO-*d*_6_).

The influence of ehr=Pro on the overall hexapeptide conformation is obvious from the comparison with the monocyclic hexapeptide of alternating stereochemistry *cyclo*(-ala-Leu-ehr-Hag-leu-Leu) (**12**), lacking the pyrrolidine ring. As expected for an 18-membered ring with a *cyclo*(-d-l)_3_ stereochemical pattern, this peptide is characterized by fast conformational averaging between three γ- and three γ^i^-turns which is evident from the smallest NH chemical shift dispersion of only 0.45 ppm which is the smallest of all cyclopeptides studied here, time averaged ^3^ *J*_NH,Hα_ couplings between 7.3 and 8.6 Hz, averaged temperature gradients between 3.0 and 3.6 ppb/K. The low dispersion of the three Leu spin systems makes their complete assignment impossible.

With the aim of separating the influence of the d-amino acids and the β-turn dipeptide ehr=Pro, we synthesized *cyclo*(-Ala-Leu-ehr=Pro-Leu-Leu) (**10**) containing ehr=Pro and an all-l-tetrapeptide. Yet again, the NMR data are characterized by significant conformational averaging (Figure 3).

**Figure 3:**
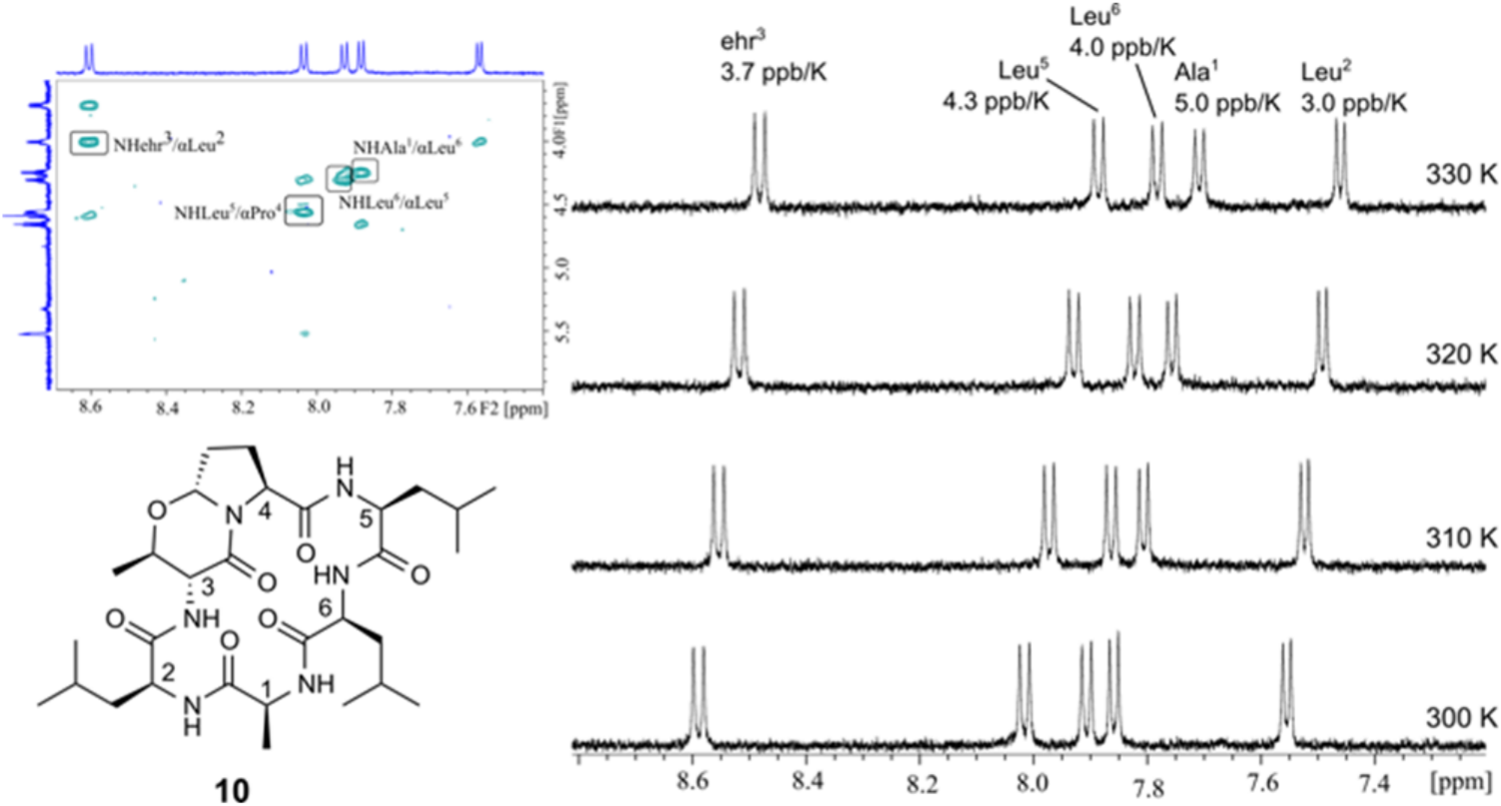
Expansion from the ROESY spectrum (600 MHz, 298 K) of **10** shows time-averaged intra- and interresidue NOEs. The temperature dependences of NH chemical shifts are averaged, too. They vary between 3 and 5 ppb/K (^1^H NMR spectrum, 500 MHz, 300–330 K, DMSO-*d*_6_).

Gratifyingly, mutant *cyclo*(-Ala-Leu-ehr=Pro-Leu-leu) (**11**) populated a single conformer with both d-configurated amino acids in the *i + 1* positions of two opposing β-turns (Figure 4). The temperature dependence identifies two strongly shielded NH protons that are not accessible to the aqueous solvent, and therefore, either have little negative dependence of the NH chemical shift with increasing measuring temperature, Δδ/Δ*T* = 1.7 ppb/K for Leu^5^, or even shift to more positive values, Δδ/Δ*T* = −2.3 ppb/K for Leu^2^. NOE-derived distances were again used for restraint molecular modeling. The dominating conformer shows ehr=Pro in a βII′-turn, opposing a second βII′-turn with leu^6^ and Ala^1^ in *i + 1* and *i + 2* positions.

**Figure 4:**
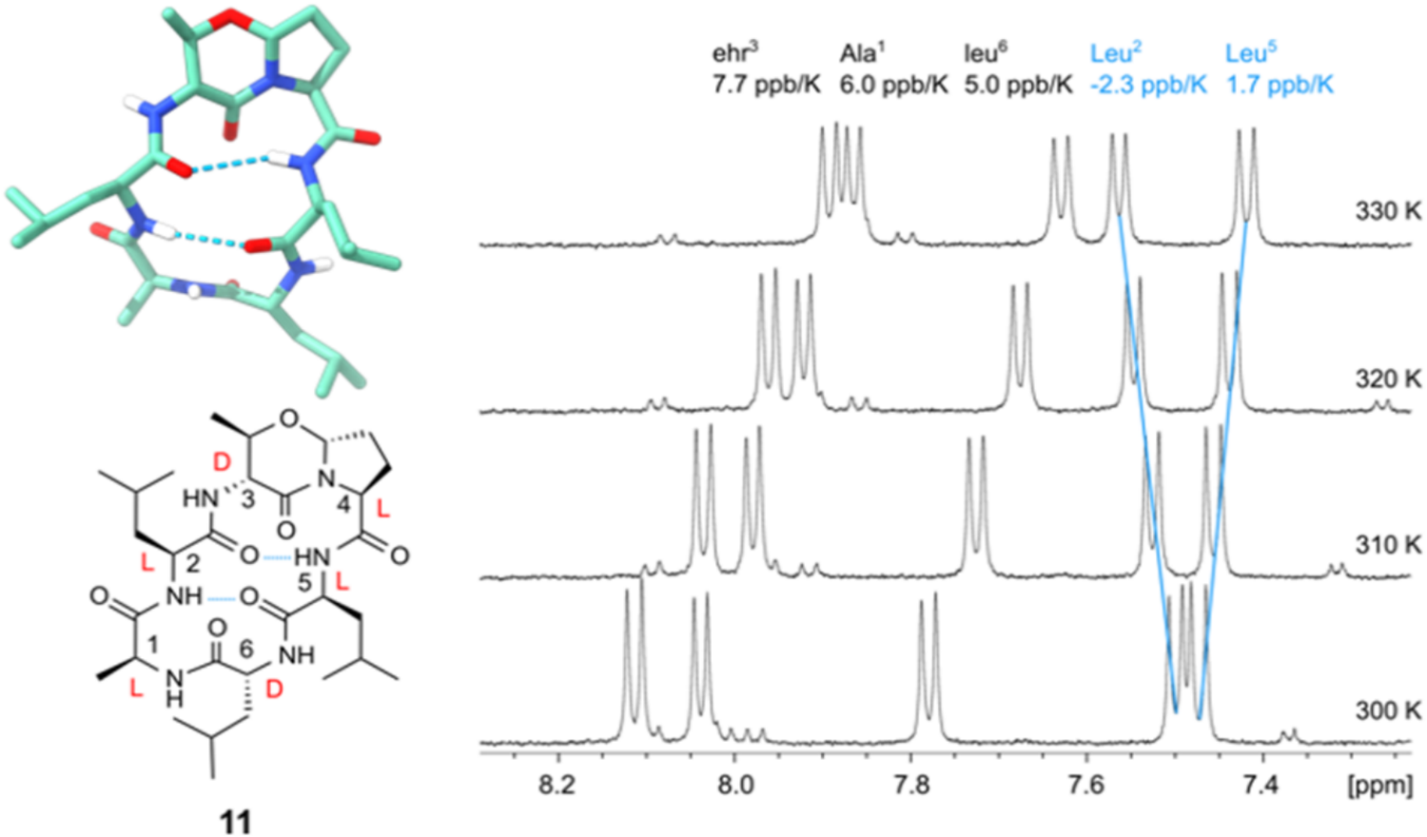
Cyclic hexapeptide **11** with the stereochemical pattern *cyclo*(-d-l-l)_2_ is the only isomer showing a double hairpin structure. (^1^H NMR spectrum, 500 MHz, 300–330 K, DMSO-*d*_6_).

Stronger than Pro but comparable to many other so-called β-turn dipeptides,^14^ ehr=Pro kinks a peptide chain to terminate secondary structural motifs and consequently prefers the *i* to *i + 1* position of a β-turn and less frequently the *i + 1* to *i + 2* position. Yet, Fusahexin-type peptides are dominated by conflicting influences caused by the alternating d, l-configuration and the constrained hydrophobic environment of the 18,6,5-tricyclic ring system. Long-range influences of ehr=Pro can be more thoroughly studied within macrocyclic peptides, but with a less restricted environment than Fusahexin. We decided to synthesize the hexapeptide *cyclo*(-ehr=Pro-Phe-Gly-Gly-Gly) (**13**), which has only one chiral amino acid next to the bicyclic dipeptide ehr=Pro. Synthesis was carried out according to the methods described in the experimental section, and the well-soluble peptide was investigated by NMR spectroscopy. Peptide **13** assumes a classical conformation of a cyclohexapeptide with two antiparallel β-turns identified by the low temperature dependence of the two high-field amide protons Phe^3^NH (−1.4 ppb/K) and Gly^6^NH (−2.0 ppb/K), which are both inaccessible to the solvent. NOEs identify a βI turn with Pro^2^ in the *i + 1* position and a βI/II flip about Gly^5^ and Gly^6^ (SI Table S15). The dipeptide ehr=Pro reminded us of the dipeptide hot=Tap (d-γ-hydroxythreonine=thiaproline), which has the same 6,5-bicyclic ring system in spite of the different functional groups and substitution pattern (Figure 5).^15^ The hexapeptide *cyclo*(-hot=Tap-Phe-Gly-Gly-Gly) (**14**) populates a dominating conformation in solution, which is characterized by two antiparallel β-turns with hot^1^ and Gly^4^ forming a pair of antiparallel hydrogen bonds.^15b^ The interesting question arose as to which turn position is preferred by ehr=Pro.

**Figure 5:**
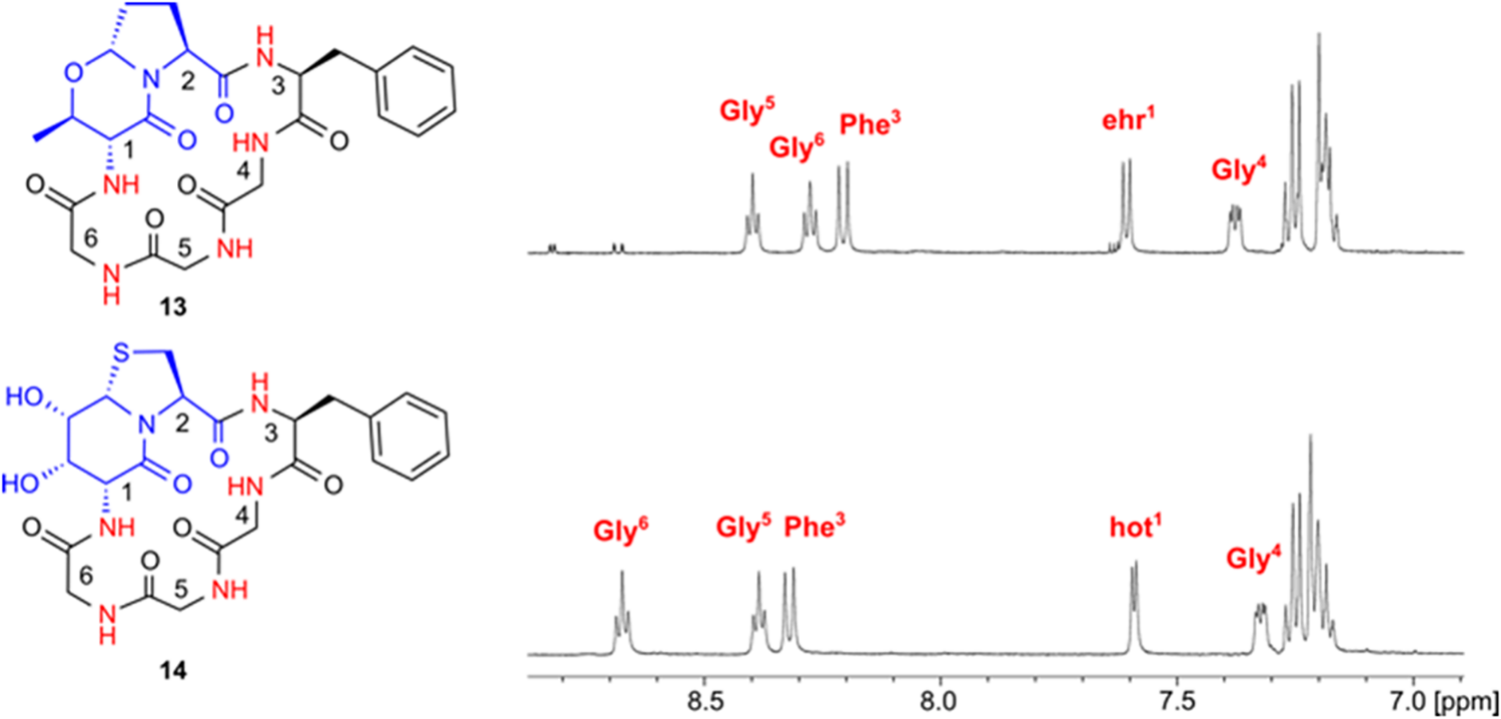
Expansions of the amide region (^1^H NMR spectrum, 300 K, 500 MHz) of *cyclo*(-ehr=Pro-Phe-Gly-Gly-Gly) (**13**) and *cyclo*(-hot=Tap-Phe-Gly-Gly-Gly) (**14**) wherein hot=Tap stands for hydroxythreonine = thiaproline. The ^3^ *J* couplings and chemical shifts are identical within ± 0.7 Hz except for ehr^1^ and hot^1^, which differ by 2.4 Hz.

The NH chemical shifts of cyclic hexapeptide *cyclo*(-ehr=Pro-Phe-Gly-Gly-Gly) (**13**) were surprisingly similar to those of *cyclo*(-hot=Tap-Phe-Gly-Gly-Gly) (**14**). Even their temperature dependences of amide protons were close to identical, identifying ehr^1^NH and Gly^4^NH as proton donors in intramolecular hydrogen bonds. The ring conformations of both peptides are nearly identical, even the orientation of the benzylic side chain rotamers is the same (Table S13). In conclusion, the bicyclic dipeptide ehr=Pro is found in the *i + 1* to *i + 2* position of a β-turn in peptide **11** and in the *i* to *i + 1* position of a β-turn in peptide **13**. In spite of considerable conformational averaging caused by the alternating d, l-configurated backbone of Fusahexin, ehr=Pro mainly occupies the *i* to *i + 1* position.

The question arises as to what extent does ehr=Pro induce turn structures in linear peptides? To this regard, we looked for similarities among the linear precursor peptides. What immediately catches the eye is the low-field shift of ehrNH in peptides **15**, **16**, and **17**, which all start with the sequence TFA.Leu-ehr=Pro-. Further NMR data, like temperature dependence of chemical shifts, ^3^ *J* couplings, and NOEs, indicate conformational averaging.

### Late-Stage Oxidation

The high diasteroselectivity of the oxazine ring formation encouraged us to try out the late-stage cyclization of a macrocyclic precursor peptide containing the amino acids ehr and Hag. The motivation is the possible photocleavage of the olefin, followed by the formation of the bicyclic ring and thus the change in the bioactivity of a target peptide. Unfortunately, the hydrophobicity of the Fusahexin derivatives makes the reaction inefficient and hardly tractable due to the low solubility of this peptide class. Therefore, we performed the late-stage oxidation on *cyclo*(-ehr-Hag-Phe-Gly-Gly-Gly) (**18**). In spite of the presence of osmate salts, the reaction could be followed directly by NMR (Figure 6) to yield *cyclo*(-ehr=Pro-Phe-Gly-Gly-Gly) (**13**) as a single product. The newly formed stereocenter was assigned on the basis of NOE contacts between the δH Pro and the γHehr and αHehr

**Figure 6:**
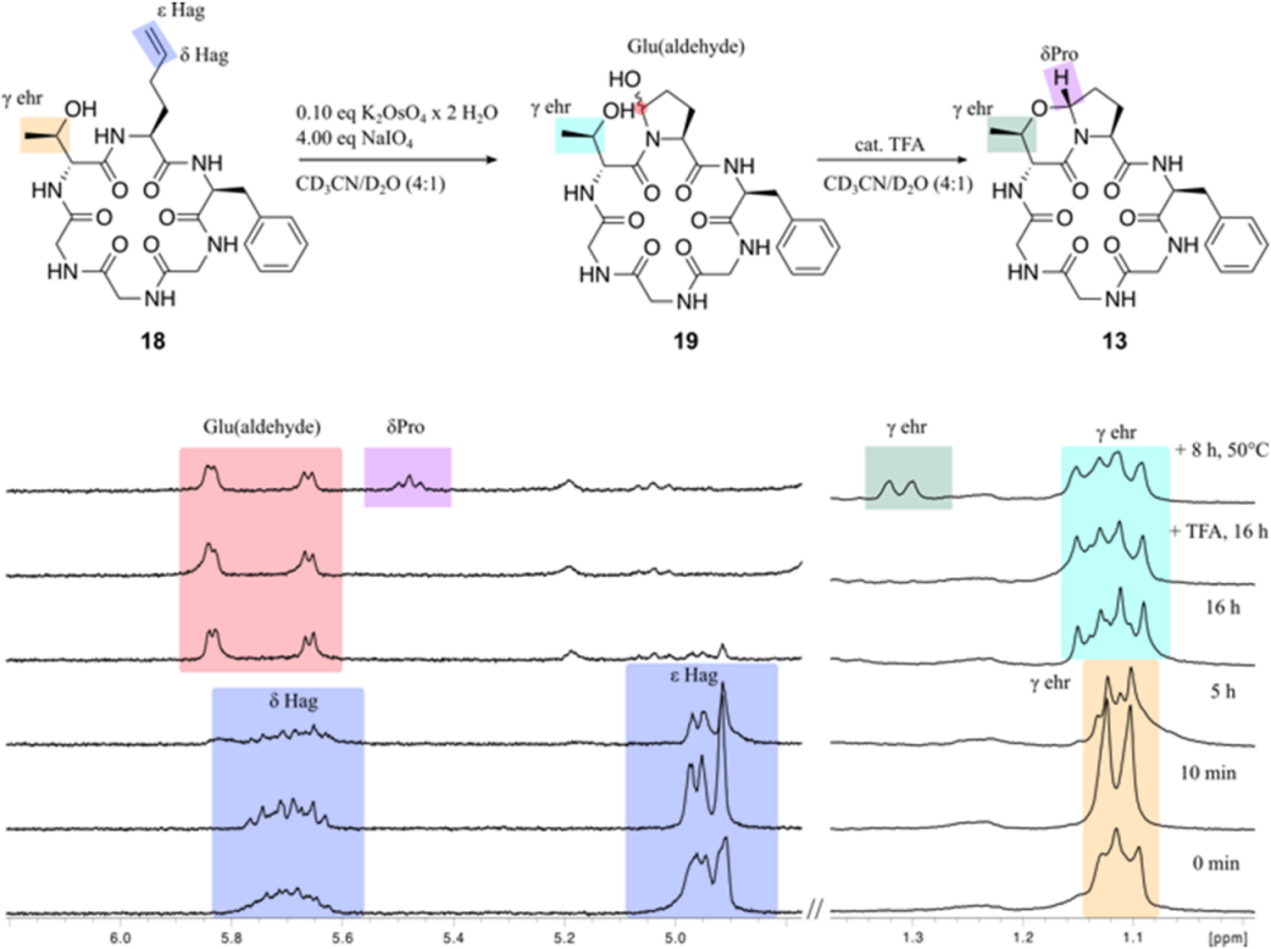
^1^H NMR expansions (300 MHz, 300 K) of the Lemieux-Johnson oxidation of *cyclo*(-ehr-Hag-Phe-Gly-Gly-Gly) (**18**). From bottom to top: 1 mg of *cyclo*(-ehr-Hag-Phe-Gly-Gly-Gly) (**18**) in CD_3_CN/D_2_O (4:1) after addition of 10 mol% K_2_OsO_4_ × 2 H_2_O and 4.00 equiv. NaIO_4_. The oxidation is complete after 16 h, and TFA is added to promote the *N*,*O*-acetal formation. The complete spectra are shown in SI.

## Conclusions

In this first synthesis of Fusahexin (**1**), we show that this peptide natural product can be formed from the Glu­(δ-aldehyde) precursor in a highly diastereoselective dehydration reaction. This observation speaks for glutamic acid, the precursor in the biosynthesis of ehr=Pro in Fusahexin, as questioned in the introduction. The bicyclic dipeptide Fmoc-ehr=Pro-OH (**2**) is a versatile building block for the synthesis of derivatives of Fusahexin (**1**) and for the characterization of ehr=Pro as a β-turn mimic. The late-stage cyclization of an ehr-Glu­(δ-aldehyde) precursor peptide allows the incorporation of the ehr=Pro turn mimic from commercial amino acids in a straightforward protocol in other peptides with the aim of using ehr=Pro as a conformation-restricting element in bioactive peptides.

## Experimental Section

### General Information

All chemicals that were used for the syntheses were purchased from commercial sources and not purified further. The proteinogenic amino acids and the 2-CTC-resin were purchased from *Iris Biotech*. Solvents, which were used for reactions under an argon atmosphere, were purchased in HPLC quality, and DMF was used in peptide grade without purification. All other solvents were purified by distillation. If not otherwise noted, all reactions were performed under an argon atmosphere at ambient pressure. Fmoc-Hag-OH was synthesized according to the literature, starting with Boc-Hag-OMe. The analytical data match the data known from the literature.^16^ Analytical thin layer chromatography TLC plates coated with silica gel 60 F_254_ (*Macherey-Nagel*) were used for the reaction control. The detection was done by fluorescence quenching under ultraviolet (UV) light (*l* = 254 nm) or by staining with potassium permanganate solution, followed by heating to 300 °C. For flash chromatography, silica gel 60 (60 Å, 230–400 mesh) from *Macherey-Nagel* was used. Unless otherwise noted, the peptides were purified by semipreparative reversed-phase HPLC with *Thermo Scientific* UltiMate3000 including a MWD-3000 detector and a *Macherey-Nagel* ACE 5 SuperC18 (150 × 10 mm^2^, 5 mm, 90 Å) or a VP Nucleodur C18 gravity column (125 × 21 mm^2^, 5 mm, 100 Å). For the analytical HPLC, an ACE UltraCore 2.5 SuperC18 (150 × 2.1 mm^2^) was used on the same system. For both HPLC systems, H_2_O + 0.1% TFA (Solvent A) and MeCN + 0.085% TFA (Solvent B) were used as the solvents. After purification, the peptides were lyophilized with a *Christ α* 2–4LD plus. Mass spectra were measured on an LTQ-FT instrument (*Thermo Fisher Scientific*). Routine ^1^H NMR and ^13^C­{^1^H} NMR spectra were measured on a *Bruker* Avance 300 (300 MHz). Measurements at 500 or 600 MHz were performed on *Bruker* Avance AV 500, *Bruker* DRX 500, *Bruker* Avance AV 600, or *Bruker* Avance NEO 600 spectrometers. The spectra were calibrated to the chemical shift of DMSO-*d*_6_: 2.50 and 39.52 ppm.

## Experimental Procedures

### Fmoc-ehr-OH (**6**)

To a stirred solution of d-*allo*-Threonine (1.00 g, 8.39 mmol, 1.0 equiv) in water (30 mL) was added Na_2_CO_3_ (1.78 g, 16.8 mmol, 2.0 equiv) and the solution was cooled down to 0 °C. A mixture of Fmoc-OSu (2.83 g, 8.39 mmol, 1.0 equiv) in MeCN (30 mL) was added slowly dropwise. After complete addition, the solution was stirred at 0 °C for 2 h before the ice bath was removed, and the reaction was stirred for 16 h at rt. The solution was diluted with water (30 mL) and the pH value was set to 2 with HCl (2 m) and then extracted with EtOAc (3 × 100 mL). The combined organic layers were washed with brine (100 mL) and dried over MgSO_4_, before the solvent was removed in vacuo. The product **6** was isolated as a white solid (2.80 g, 8.20 mmol, 97%). ^1^H NMR (500 MHz, 300 K, DMSO-*d*_6_) δ 7.89 (d, ^3^ *J* = 7.5 Hz, 2H, Fmoc^Ar^), 7.74 (d, ^3^ *J* = 7.5 Hz, 2H, Fmoc^Ar^), 7.50 (d, ^3^ *J* = 8.5 Hz, 1H, NH), 7.42 (dd, ^3^ *J* = 8.20 Hz, ^3^ *J* = 7.4 Hz, 2H, Fmoc^Ar^), 7.33 (dd, ^3^ *J* = 8.20 Hz, ^3^ *J* = 7.3 Hz, 2H, Fmoc^Ar^), 4.95 (b, 1H, OH), 4.28–4.20 (m, 3H, CH-Fmoc, CH_2_–Fmoc), 3.95–3.90 (m, 2H, αH, βH), 1.10 (d, ^3^ *J* = 6.2 Hz, 3H, γH). ^13^C­{^1^H} NMR (75 MHz, 300 K, DMSO-*d*_6_) δ: 172.3, 156.1, 156.1, 143.8, 140.7, 127.6, 127.1, 125.3, 120.1, 66.5, 65.8, 60.3, 46.6, 19.7. HRMS (ESI) *m*/*z*: [M + Na]^+^ Calcd for C_19_H_19_NO_5_Na 364.1155; Found 364.1150.

### Boc-Hag-OMe (**4**)

Boc-Iodalanine-OMe (**3**) was synthesized according to the literature.^5,6^ Activated zinc (1.19 g, 18.2 mmol, 3.00 equiv) was heated with a heat gun at 450 °C for 5 min. After cooling to room temperature, the zinc was suspended in dry DMF (10 mL) and iodine (0.15 g, 1.22 mmol, 0.20 equiv) was added. After the color had disappeared, a solution of iodalanin (2.00 g, 6.08 mmol, 1.00 equiv) in dry DMF (50 mL) was dripped in. The solution was stirred until full conversion was detected on the TLC. The liquid was transferred to a mixture of copper bromide (0.44 g, 3.04 mmol, 0.50 equiv), which was heated with a heat gun at 450 °C for 5 min before, and allyl bromide (0.79 mL, 9.12 mmol, 1.50 equiv) in dry DMF (10 mL) at – 15 °C. Once the addition was completed, the reaction solution was allowed to warm to room temperature and stirred for 16 h. The mixture was diluted with EtOAc (50 mL) and 1 m HCl (50 mL). After separating, the aqueous phase was extracted 3 times with EtOAc (50 mL). The combined organic phase was washed with 1 m Na_2_S_2_O_3_ (50 mL), water (50 mL), and brine (50 mL) and dried over MgSO_4_. The solvent was removed under reduced pressure, and the crude product was purified with column chromatography (cyclohexane/ethyl acetate 9:1) to give the pure product **4** as a light yellow oil (1.20 g, 4.93 mmol, 81%). **R**_***f***_: 0.46 (cyclohexane:ethyl acetate 4:1 v/v). ^1^H NMR **(**500 MHz, 300 K, DMSO-*d*_6_) δ 7.27 (d, ^3^ *J* = 7.82 Hz, 1H, NH), 5.81:5.72 (m, 1H, δH), 5.02:4.97 (m, 2H, εH), 3.97:3.93 (m, 1H, αH), 3.61 (s, 3H, OMe), 2.09:2.00 (m, 2H, γH), 1.74:1.61 (m, 2H, βH), 1.38 (s, 9H, Boc). ^13^C­{^1^H} NMR (75 MHz, 300 K, DMSO-*d*_6_) δ: 173.5, 155.5, 137.3, 115.5, 78.1, 52.6, 51.5, 29.6, 29.3, 27.9. HRMS (ESI) *m*/*z*: [M + Na]^+^ Calcd for C_12_H_21_NO_4_Na 266.1363; Found 266.1365.

### H-Hag-OMe x HCl (**5**)

Boc-Hag-OMe (8.92g, 36.6 mmol, 1.00 equiv., **4**) was dissolved in MeOH (230 mL) and cooled down to 0 °C. Afterward thionyl chloride (7.18 mL, 99.0 mmol, 2.70 equiv) was added and the solution was warmed up to room temperature for 16h.^17^ The solvent was removed under vacuum, and the crude product **5** (6.01 g, 33.5 mmol, 91%) was used as a chloride salt without any further purification. ^1^H NMR (500 MHz, DMSO-*d*_6_) δ 8.75 (s, 3H, NH_3_), 5.78 (dddd, ^3^ *J* = 17.2 Hz, ^3^ *J* = 10.4 Hz, ^3^ *J* = 6.7 Hz, ^3^ *J* = 6.5 Hz, 1 H, δH), 5.10–5.01 (m, 2H, εH), 3.98–3.96 (m, 1H, αH), 3.74 (s, 3H, OMe), 2.24–2.17 (m, 1H, γH), 2.12–2.05 (m, 1H, γH), 1.92 (m, 2H, βH). ^13^C­{^1^H} NMR (125 MHz, 300 K, DMSO-*d*_6_) δ: 170.3, 137.2, 116.5, 53.2, 51.8, 29.6, 28.6. HRMS (ESI) *m*/*z*: [M]^+^ Calcd for C_7_H_14_NO_2_ 144.1019; Found 144.1017.

### Fmoc-ehr-Hag-OMe (**7**)

Fmoc-ehr-OH (**6**) (0.78 g, 2.28 mmol, 1.00 equiv) and H_2_N-Hag-OMe x HCl (**5**) (0.41 g, 2.28 mmol, 1.00 equiv) were dissolved in DMF (20 mL) and cooled down to 0 °C. Then HBTU (0.95 g, 2.51 mmol, 1.10 equiv) and triethylamine (0.66 mL, 4.79 mmol, 2.10 equiv) were added and the reaction was stirred for 72 h at room temperature.^18^ Afterward, the solution was diluted with EtOAc (30 mL) and NaOAc (30 mL) and the layers were separated. The aqueous layer was extracted 3 times with EtOAc (30 mL) and the organic layers were washed with water (30 mL) and brine (40 mL), dried over MgSO_4_, and the solvent was removed in vacuum. The crude product was purified with column chromatography (cyclohexane/ethyl acetate 1:1 v/v), and the pure product **7** was isolated as a light yellow solid (0.78 g, 1.66 mmol, 73%). **R**_***f***_: 0.35 (cyclohexane:ethyl acetate 1:1 v/v). ^1^H NMR (500 MHz, 300 K, DMSO-*d*_6_) δ 8.20 (d, ^3^ *J* = 7.6 Hz, 1H, NH Hag), 7.88 (d, ^3^ *J* = 7.5 Hz, 2H, Fmoc^Ar^), 7.75–7.74 (m, 2H, Fmoc^Ar^), 7.42 (dd, ^3^ *J* = 8.2 Hz, ^3^ *J* = 7.5 Hz, 2H, Fmoc^Ar^), 7.34–7.31 (m, 3H, Fmoc^Ar^, NH ehr), 5.76 (dddd, ^3^ *J* = 17.0 Hz, ^3^ *J* = 10.2 Hz, ^3^ *J* = 6.7 Hz, ^3^ *J* = 6.5 Hz, 1H, δH), 5.02–4.97 (m, 2H, εH), 4.81 (d, ^3^ *J* = 5.4 Hz, 1H, OH), 4.29–4.20 (m, 4H, Fmoc-CH_2_, Fmoc-CH, αH Hag), 4.05 (dd, ^3^ *J* = 9.5 Hz, ^3^ *J* = 6.8 Hz, 1H, αH ehr), 3.85 (dq, ^3^ *J* = 13.2 Hz, ^3^ *J* = 6.30, 1H, βH ehr), 3.62 (s, 3H, OMe), 2.10–1.99 (m, 2H, γH Hag), 1.80–1.67 (m, 2H, βH Hag), 1.07 (d, ^3^ *J* = 6.3 Hz, 3H, γH ehr). ^13^C­{^1^H} NMR (75 MHz, 300 K, DMSO-*d*_6_) δ: 172.4, 170.4, 143.8, 143.8, 140.7, 137.3, 127.6, 127.0, 125.3, 120.1, 115.6, 66.7, 65.8, 60.4, 51.8, 51.2, 46.6, 30.2, 29.2, 19.6. HRMS (ESI) *m*/*z*: [M + Na + H]^2+^ Calcd for C_26_H_30_N_2_O_6_NaH 490.2022; Found 490.2028.

### Fmoc-ehr=Pro-OMe (**9**)

Fmoc-ehr-Hag-OMe (0.83 g, 1.77 mmol, 1.00 equiv, **7**) was dissolved in MeCN/water (8 mL, 3:1, v/v), and sodium periodate (0.95 g, 4.44 mmol, 2.50 equiv) was added. Then K_2_OsO_4_ × 2 H_2_O (0.03 g, 0.09 mmol, 0.05 equiv) was added and the solution was stirred for 12 h at room temperature. The suspension was diluted with water (20 mL) and ethyl acetate (20 mL) and the phases were separated. The aqueous phase was extracted 3 times with ethyl acetate (20 mL) and the combined organic layers were washed with 1 m HCl (50 mL) and dried over MgSO_4_.^19^ After the solvent was removed in vacuo, the crude product was purified with column chromatography (cyclohexane:ethyl acetate, 4:1 to 1:1 v/v) to isolate the product **9** as a white foam (0.46 g, 1.02 mmol, 57%). **R**_***f***_: 0.49 (cyclohexane:ethyl acetate 1:1 v/v).^1^H NMR (500 MHz, 300 K, DMSO-*d*_6_) δ 7.89 (d, ^3^ *J* = 7.5 Hz, 2H, Fmoc^Ar^), 7.72 (d, ^3^ *J* = 7.4 Hz, 2H, Fmoc^Ar^), 7.59 (d, ^3^ *J* = 9.2 Hz, 1H, NH), 7.42 (dd, ^3^ *J* = 8.1 Hz, ^3^ *J* = 7.5 Hz, 2H, Fmoc^Ar^), 7.33 (dd, ^3^ *J* = 8.1 Hz, ^3^ *J* = 7.4 Hz, 2H, Fmoc^Ar^), 5.56 (dd, ^3^ *J* = 5.8 Hz, ^3^ *J* = 3.1 Hz, 1H, δH Pro), 4.57 (dd, ^3^ *J* = 7.3 Hz, ^3^ *J* = 4.5 Hz, 1H, αH Pro), 4.36–4.34 (m, 2H, Fmoc-CH_2_), 4.25­(dd, 1H, ^3^ *J* = 8.0 Hz, ^3^ *J* = 7.0 Hz, Fmoc-CH), 4.21 (dd, ^3^ *J* = 10.0 Hz, ^3^ *J* = 9.2 Hz, 1H, αH ehr), 3.83–3.74 (m, 1H, βH ehr), 3.65 (s, 3H, OMe), 2.30–2.15 (m, 2H, βH Pro, γH Pro), 1.93–1.79 (m, 2H, βH Pro, γH Pro), 1.26 (d, ^3^ *J* = 6.2 Hz, 3H, γH ehr). ^13^C­{^1^H} NMR (125 MHz, 300 K, DMSO-*d*_6_) δ: 171.5, 167.4, 156.5, 143.9, 143.7, 140.8, 127.7, 127.1, 125.2, 120.2, 120.2, 83.2, 71.3, 65.7, 57.7, 55.5, 55.2, 52.2, 46.6, 30.9, 26.5, 18.8. HRMS (ESI) *m*/*z*: [M + Na]^+^ Calcd for C_25_H_26_N_2_O_6_Na 473.1683; Found 473.1671.

### Hemiaminal **8**

To isolate the half aminal **8** the following procedure was used.^3,20^ The dipeptide Fmoc-ehr-Hag-OMe (**7**, 0.224 g, 0.480 mmol, 1.00 equiv) was dissolved in MeCN/H_2_O (12 mL, 4:1 v/v) and sodium periodate (0.411 g, 1.92 mmol, 4.00 equiv) was added. Afterward, the salt K_2_OsO_4_ × 2 H_2_O (0.018 g, 0.048 mmol, 0.10 equiv) was added to the light yellow solution and stirred for 4 h at room temperature. The reaction was quenched with 5%(w/w) Na_2_SO_3_ solution (5 mL) and stirred for 30 min before EtOAc (10 mL) was added. The layers were separated and the aqueous layer was re-extracted 2 times with EtOAc (10 mL). The combined organic layer was washed with water (10 mL) and dried over MgSO_4,_ and the solvent was removed under reduced pressure. The crude product as a yellow solid (0.125 g, 0.324 mmol, 67%) was used without any further purification. HRMS (ESI) *m*/*z*: [M + Na]^+^ Calcd for C_25_H_28_N_2_O_7_Na 491.1789; Found 491.1779.

### Fmoc-ehr=Pro-OH (**2**)

Fmoc-ehr=Pro-OMe (0.31 g, 0.69 mmol, 1.00 equiv, **9**) was dissolved in THF (6.50 mL) and cooled down to 0 °C. A solution of LiOH x H_2_O (58.3 mg, 1.39 mmol, 2.00 equiv) in water (6.50 mL), which was also cooled down to 0 °C, was added dropwise and the reaction was stirred for 10 min at 0 °C. After complete conversion the solution was brought to pH 2 with 1 m HCl and extracted 2 times with ethyl acetate (20 mL). The organic phase was washed with brine (20 mL) and dried over MgSO_4_, and the solvent was removed under reduced pressure. The crude product was purified with column chromatography (DCM/methanol/acidic acid 10:1:0.1 v/v/v) to give the pure product **2** as a white foam (0.20 g, 0.47 mmol, 67%).^1^H NMR (500 MHz, 300 K, DMSO-*d*_6_) δ 12.7 (bs, 1H, COOH), 7.89 (d, ^3^ *J* = 7.5 Hz, 2H, Fmoc^Ar^), 7.72 (dd, ^3^ *J* = 7.3 Hz, ^3^ *J* = 4.2 Hz, 2H, Fmoc^Ar^), 7.56 (d, ^3^ *J* = 9.3 Hz, 1H, NH ehr), 7.42 (dd, ^3^ *J* = 8.4 Hz, ^3^ *J* = 7.5 Hz, 2H, Fmoc-^Ar^), 7.33 (dd, ^3^ *J* = 8.5 Hz, ^3^ *J* = 7.5 Hz, 2H, Fmoc-^Ar^), 5.53–5.51 (m, 1H, δH Pro), 4.47 (m, 1H, αH Pro), 4.36–4.34 (m, 2H, Fmoc-CH_2_), 4.26 (dd, ^3^ *J* = 7.9 Hz, ^3^ *J* = 7.0 Hz, 1H, Fmoc-CH), 4.18 (dd, ^3^ *J* = 10.1 Hz, ^3^ *J* = 9.2 Hz, 1H, αH ehr), 3.81–3.75 (m, 1H, βH ehr), 2.26–2.16 (m, 2H, βH Pro, γH Pro), 1.91–1.80 (m, 2H, βH Pro, γH Pro), 1.26 (d, ^3^ *J* = 6.2 Hz, 3H, γH ehr). ^13^C­{^1^H} NMR (125 MHz, 300 K, DMSO-*d*_6_) δ: 172.4, 167.0, 156.4, 143.8, 143.6, 140.7, 127.6, 127.0, 125.1, 120.1, 120.0, 83.0, 71.2, 65.7, 57.8, 55.5, 46.6, 30.8, 26.4, 18.8. HRMS (ESI) *m*/*z*: [M – H]^−^ Calcd for C_24_H_23_N_2_O_6_ 435.1562; Found 435.1563.

### Synthesis of the Peptides

#### General Procedure for the Solid Phase Peptide Synthesis (GP1)

##### Resin Loading with Fmoc Amino Acids

2-CTC-resin was preloaded with l-alanine, d-alanine, or l-glycine. At first, 2-CTC-resin (1 g, B = 1.52 mmol/g) from *IRIS* was suspended in DMF and shaken for 30 min. Then DIPEA (6.00 equiv) and the Fmoc amino acid (2.00 equiv) were added and the reaction mixture was shaken at rt for 3 h, before the solvent was removed. The resin was washed 3 times with DMF, MeOH, and DCM. A solution of DCM/MeOH/DIPEA (80:15:5) was added to the resin and it was shaken for 30 min. The solvent was removed and the procedure was done a second time. Thereafter, the resin was washed 3 times with DMF and MeOH and 5 times with DCM. The preloaded resin was dried under reduced pressure.

To detect the loading of the CTC-resin, a specific amount of resin was suspended in piperidine in DMF (25% v/v, 1 mL) for 45 min at room temperature. Afterward, MeOH (5 mL) was added and UV–vis spectra were measured at 289 and 300 nm 3 times. With the following mathematical formula, the new loading of the resin was calculated.

$$B\left[\mathrm{mmol}/g\right]=\frac{A_{289/300\mathrm{nm}}}{\varepsilon_{289/300\left[M^{-1}{\mathrm{cm}}^{-1}\right]\cdot D\left[\mathrm{cm}\right]}}\cdot \frac{V_{\mathrm{solution}}\left[\mathrm{mL}\right]}{m_{\mathrm{resin}}\left[g\right]}$$

extinction coefficient: *e*_289_ = 5800 L·M^–1^ cm^–1^, e_300_ = 7800 L·M^–1^ cm^–1^; A = absorption at this specific wavelength, *D* = 1 cm (thickness of the cuvette); *V*_solution_ = 6 mL.

##### General Procedure for the SPPS with Ultrasonic (GP1.1)

The SPPS with ultrasonic bath was done following a modified literature-known procedure.^21^

##### Cleavage of the Fmoc Protection Group

At first, the Fmoc protection group was cleaved from the resin by treating the preloaded resin with piperidine in DMF (20% v/v, 3 mL) for 1 min in the ultrasonic bath. This was done a second time for 3 min with ultrasonication, before the resin was washed 3 times with DMF (5 mL), MeOH (5 mL), and DCM (5 mL).

##### Coupling of the Amino Acids

Proteinogenic amino acids Fmoc-ehr-OH and Fmoc-Hag-OH: The amino acid (0.25 mmol) was dissolved in DMF (3 mL) and the coupling reagents HBTU (95.0 mg, 0.25 mmol, 2.50 equiv) and HOBt x H_2_O (34.0 mg, 0.22 mmol, 2.20 equiv) were added. After DIPEA (86 μL) was added, the amino acid was pre activated for 5 min and then given to the resin. The reaction solution was treated for 5 min with ultrasonics, and then the solvent was removed. The resin was washed again 3 times with DMF (5 mL), MeOH (5 mL), and DCM (5 mL).

Nonproteinogenic amino acid Fmoc-ehr=Pro-OH: The amino acid (0.10 mmol) was dissolved in DMF (3 mL) and the coupling reagents HBTU (95.0 mg, 0.25 mmol, 2.50 equiv) and HOBt x H_2_O (34.0 mg, 0.22 mmol, 2.20 equiv) were added. After DIPEA (86 μL, 0.50 mmol, 5.00 equiv) was added, the amino acid was pre activated for 5 min and then given to the resin. The reaction solution was treated for 5 min with ultrasonic and then the solvent was removed. The resin was washed again 3 times with DMF (5 mL), MeOH (5 mL), and DCM (5 mL). The procedure for the coupling of the amino acid was repeated until the entire peptide sequence was synthesized.

##### General Procedure of the Automated Solid Phase Peptide Synthesis (GP1.2)

The automatic synthesis of the peptides was done with a *CEM Liberty Blue* peptide synthesizer assisted with a microwave. The preloaded CTC-resin was swollen in DMF (15 mL) at room temperature for 20 min before the following cycles of Fmoc-deprotection and amino acid coupling were done.

Fmoc-deprotection: *T* = 50 °C, *P*_microwave_ = 30 W, t = 210 s with piperidine (20 wt % in DMF, 3.00 mL/deprotection).

Amino acid coupling: *T* = 50 °C, *P*_microwave_ = 30 W, t = 600 s with Fmoc-protected amino acid (5.00 equiv, 0.2 m in DMF, 2.5 mL/coupling), Oxyma (5.00 equiv, 1.0 m in DMF, 0.5 mL/coupling), and DIC (5.00 equiv, 1.0 m in DMF, 0.5 mL/coupling).

##### Cleavage of the Resin

The cleavage of the peptides was done according to the following procedure. The CTC-resin was treated 3 times with TFA (20 μL) in DCM (1.9 mL) for 2 min at rt. The solution was set to pH 8 by adding DIPEA. The solvent was removed under reduced pressure to yield the crude product as a solid.

##### General Procedure of the Macrocyclization of the Peptides (GP2)

The cyclization was done following literature-known reaction conditions.^9^ The peptide was dissolved in DMF to get a concentration of 0.001 mol/L. Afterward, HATU (2.00 equiv) and DIPEA (6.00 equiv) were added, and the resulting yellow solution was stirred at rt until full conversion was detected. The solvent was removed under reduced pressure, and the crude product was purified after the following procedures.

##### Purification of the Peptides with Semipreparative HPLC (GP3)

The peptides were purified by semipreparative reversed-phase HPLC with *Thermo Scientific* UltiMate3000 including a MWD-3000 detector and a *Macherey-Nagel* ACE 5 SuperC18 (150 × 10 mm^2^, 5 μm, 90 Å) or a VP Nucleodur C18 gravity column (125 × 21 mm^2^, 5 μm, 100 Å).

##### Wash the Peptide (GP4)

The peptides that had poor solubility in water/MeCN were purified with another procedure. The crude peptide was suspended in a water/MeCN (4:1) mixture with TFA (1%). The suspension was treated for 10 min with ultrasonic. After centrifugation, the supernatant was removed, and the solid was suspended again in water/MeCN (4:1) with TFA (1%) and treated for 10 min with ultrasonic. This procedure was performed a third time before the pure peptide was isolated as a white solid.

##### TFA.H-Leu-ehr=Pro-leu-Leu-ala-OH (**15**)

The peptide **15** was synthesized on a 0.10 mmol scale following the general procedure (GP1.1) and purified by washing the crude peptide following the procedure described above (GP4). The pure peptide was isolated as a white solid (9.5 mg).

##### Cyclo­(-ala-Leu-ehr=Pro-leu-Leu) (**1**)

The linear peptide (9.5 mg, 0.015 mmol, 1.00 equiv) was cyclized with HATU (2.00 equiv) and DIPEA (6.00 equiv) in DMF (0.001 mol/L) as described above (GP2). The cyclic product Fusahexin was purified after the general procedure GP4 and was isolated as a white solid (9.01 mg, 0.0148 mmol, 97%).

##### TFA.H-Leu-ehr-Hag-leu-Leu-ala-OH (**20**)

The peptide **20** was synthesized with the automated solid phase peptide synthesis as described in the GP1.2. The peptide was cleaved from the resin with TFA/TIPS/H_2_O (92/4/4, 5 mL) for 30 min at room temperature. The solvent was removed *in vacuo*, and the crude peptide was isolated as a light yellow solid and used without any further purification.

##### Cyclo­(-ala-Leu-ehr-Hag-leu-Leu) (**12**)

The linear hexapeptide **20** was cyclized as described in the GP2. Afterward, the peptide was cleaved from the resin with TFA/TIPS/H_2_O (92/4/4, 5 mL) for 30 min at room temperature. The solvent was removed *in vacuo* to yield crude product **12**, which was used without further purification because of its poor solubility in MeCN or water.

##### TFA.H-Leu-ehr=Pro-Leu-Leu-Ala-OH (**16**)

The linear peptide **16** was synthesized as described in the general procedure 1.1 (GP1.1) and cleaved as described above (GP2). The crude peptide was purified with the semipreparative HPLC (GP3) to isolate the peptide (11.4 mg) as a white foam.

##### Cyclo­(-Ala-Leu-ehr=Pro-Leu-Leu) (**10**)

Peptide **16** was cyclized according to the general procedure described before (GP2). The pure peptide **10** was isolated as a white foam (0.9 mg) after purification with rp HPLC (GP4).

##### TFA.H-Leu-ehr=Pro-Leu-leu-Ala-OH (**17**)

The linear peptide was synthesized according to the GP1.1 and cleaved from the resin as described in the general procedure GP1. The crude product was purified with HPLC (GP3) to isolate the pure peptide **17** as a white foam (3.22 mg).

##### Cyclo­(-Ala-Leu-ehr=Pro-Leu-leu) (**11**)

The peptide **17** was cyclized according to the general procedure described before (GP2). The crude product was purified with HPLC (GP3) to yield pure peptide **11** as a white foam (1.85 mg).

##### TFA.H-ehr=Pro-Phe-Gly-Gly-Gly-OH (**21**)

Peptide **21** was synthesized according to the general procedure (GP1, GP1.1) and purified with rp HPLC (GP3) to isolate the pure peptide as a white foam (1.01 mg).

##### Cyclo­(-ehr=Pro-Phe-Gly-Gly-Gly) (**13**)

The peptide **21** was cyclized as described in the general procedure (GP2). The crude product was purified with HPLC (GP3) and peptide **13** was isolated as a white foam (0.9 mg).

##### Cyclo­(-ehr-Hag-Phe-Gly-Gly-Gly) (**18**)

The linear peptide **22** was synthesized according to the general procedure (GP1.2). The crude peptide was cyclized as described above (GP2). After the cyclization was complete, the peptide **18** was purified with HPLC (GP3) to isolate the pure peptide as a white foam (5.6 mg).

## Supplementary Material



## Data Availability

The data underlying this study are available in the published article and its Supporting Information.

## Notes

NMR spectra for all compounds; including temperature gradients, and NMR experiments (PDF)

## References

[1] Westphal K. R., Bachleitner S., Severinsen M. M., Brundtø M. L., Hansen F. T., Sørensen T., Wollenberg R. D., Lysøe E., Studt L., Sørensen J. L., Sondergaard T. E., Wimmer R. (2021). Cyclic, Hydrophobic Hexapeptide Fusahexin
Is the Product of a Nonribosomal Peptide Synthetase in. J. Nat.
Prod..

[2] Haack M., Enck S., Seger H., Geyer A., Beck-Sickinger A. G. (2008). Pyridone
Dipeptide Backbone Scan To Elucidate Structural Properties of a Flexible
Peptide Segment. J. Am. Chem. Soc..

[3] Baldwin J. E., Hulme C., Schofield C. J., Edwards A. J. (1993). Synthesis of Potential
β-Turn Bicyclic Dipeptide Mimetics. J.
Chem. Soc., Chem. Commun..

[4] Slomczynska U., Chalmers D. K., Cornille F., Smythe M. L., Beusen D. D., Moeller K. D., Marshall G. R. (1996). Electrochemical Cyclization of Dipeptides
To Form Novel Bicyclic, Reverse-Turn Peptidomimetics. 2. Synthesis
and Conformational Analysis of 6,5-Bicyclic Systems. J. Org. Chem..

[5] Danner P., Morkunas M., Maier M. E. (2013). Synthesis
of d-Abrines by Palladium-catalyzed
Reaction of ortho-Iodoanilines with *N*-Boc-*N*-methylalanyl-substituted Acetaldehyde and Acetylene. Org. Lett..

[6] Daus F., Xie X., Geyer A. (2022). The silica
mineralisation properties of synthetic Silaffin-1A1
(synSil-1A_1_). Org. Biomol. Chem..

[7] Salih N., Adams H., Jackson R. F. W. (2016). Synthesis
of ω-Oxo Amino Acids
and *trans*-5-Substituted Proline Derivatives Using
Cross-Metathesis of Unsaturated Amino Acids. J. Org. Chem..

[8] Chiou W.-H., Mizutani N., Ojima I. (2007). Highly Efficient
Synthesis of Azabicyclo­[x.y.0]­alkane
Amino Acids and Congeners by Means of Rh-Catalyzed Cyclohydrocarbonylation. J. Org. Chem..

[9] Rohrbacher F., Deniau G., Luther A., Bode J. W. (2015). Spontaneous head-to-tail
cyclization of unprotected linear peptides with the KAHA ligation. Chem. Sci..

[10] Kessler H. (1982). Conformation
and Biological Activity of Cyclic Peptides. Angew. Chem., Int. Ed. Engl..

[11] Gurrath M., Müller G., Kessler H., Aumailley M., Timpl R. (1992). Conformation/Activity
Studies of Rationally Designed Potent Anti-Adhesive
RGD Peptides. Eur. J. Biochem..

[12] Jwad R., Weissberger D., Hunter L. (2020). Strategies for Fine-Tuning the Conformations
of Cyclic Peptides. Chem. Rev..

[13] Eckhardt B., Grosse W., Essen L.-O., Geyer A. (2010). Structural characterization
of a β-turn mimic within a protein–protein interface. Proc. Natl. Acad. Sci. U.S.A..

[14] Nagai U., Sato K., Nakamura R., Kato R. (1993). Bicyclic Turned Dipeptide
(BTD) as a β-Turn Mimetic; its Design Synthesis and Incorporation
into Bioactive Peptides. Tetrahedron.

[15] b Wuttke, A. Synthetische Oligomere von amyloidogenen Peptiden durch reversible Chemie. Ph.D. Thesis, Philipps-University Marburg, 2016.

[16] Atmuri N.
D. P., Lubell W. D. (2015). Insight
into Transannular Cyclization Reactions To
Synthesize Azabicyclo­[*X*.*Y*.*Z*]­alkanone Amino Acid Derivatives from 8-, 9-, and 10-Membered
Macrocyclic Dipeptide Lactams. J. Org. Chem..

[17] Lei M., Zhang H., Miao H., Du X., Zhou H., Wang J., Wang X., Feng H., Shi J., Liu Z., Shen J., Zhu Y. (2019). Preparation and biological evaluation
of soluble tetrapeptide epoxyketone proteasome inhibitors. Bioorg. Med. Chem..

[18] Manzor K., Kelleher F. (2016). Synthesis of orthogonally protected
thioamide dipeptides
for use in solid-phase peptide synthesis. Tetrahedron
Lett..

[19] Kim D., Lee J. M., Song J., Lee S. W., Lee H. G., Kim K. T. (2022). Synthesis
of Enantiomeric w-Substituted Hydroxy Acids
from Terminal Epoxides and Alkenes: Functional Building Blocks for
Discrete and Sequence-Defined Polyesters. Macromolecules.

[20] Claridge T. D. W., Hulme C., Kelly R. J., Lee V., Nash I. A., Schofield C. J. (1996). Synthesis and Analysis of Leu-Enkephalin analogues
containing reverse turn peptidomimetics. Bioorg.
Med. Chem. Lett..

[21] Merlino F., Tomassi S., Yousif A. M., Messere A., Marinelli L., Grieco P., Novellino E., Cosconati S., Di Maro S. (2019). Boosting Fmoc Solid-Phase Peptide
Synthesis by Ultrasonic. Org. Lett..

