# Supplementary material for: Synthesis of Fusahexin and Characterization of a Natural β‑Turn Dipeptide
Source: J Org Chem. 2025 Jul 29;90(31):11142–50. doi: 10.1021/acs.joc.5c01080 (PMC12340964; doi:10.1021/acs.joc.5c01080)
Supplement: Supplementary file 1 [file jo5c01080_si_001.pdf]

Supporting Information

Synthesis of Fusahexin and Characterization of a Natural β-Turn Dipeptide

Carmen Bäuerlein[a] and Armin Geyer[a*]

[a] Philipps-University Marburg, Department of Chemistry, Hans-Meerwein-Straße 4, 35043 Marburg (Germany)

[*] Corresponding author. E-mail: geyer@staff.uni-marburg.de

Table of contents

1. Synthesis of peptides S3
2. Analytical data S15
3. Temperature gradients S34
4. Late-stage Oxidation of peptide **18** S42
5. ^{1}H NMR spectra of synthetic and isolated Fusahexin S43
6. Computer modeling S44
7. References S45

1. Synthesis of peptides

TFA.H-Leu-ehr=Pro-leu-Leu-ala-OH (**15**)

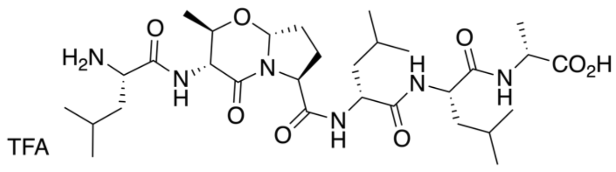

15

Table S1: ^{1}H NMR signal assignment for peptide **15** from TOCSY and HSQC spectrum (600 MHz, 300 K, DMSO-d_6), n.d = not definable.

amino acid	NH / ppm	αH / ppm	βH / ppm	further / ppm
ala[1]	7.99	4.15	1.25	
Leu[2]	n.d	3.81	1.66	γH: 1.56<br>δH: 0.96
ehr[3]	8.83	4.61	3.71	γH: 1.24
Pro[4]	n.d	4.59	2.16, 1.82	γH: 2.16, 1.82<br>δH: 5.51
leu[5]	8.15	4.24	1.46	γH: 1.46<br>δH: 0.90, 0.86
Leu[6]	8.26	4.24	1.55	γH: 1.46<br>δH: 0.83, 0.80

HRMS (ESI) m/z: $[M - H]^-$ Calcd for $C_{30}H_{51}N_6O_8$ 623.3774; Found 623.3781.

analytical *rp* HPLC: t_R=9.205 min (0.45 mL/min, 15-65% MeCN in 20 min).

semi-preparative *rp* HPLC: 7 mL/min, 15-65% MeCN in 20 min.

purity detected by *rp*-HPLC: 86%.

cyclo(-ala-Leu-ehr=Pro-leu-Leu) (**1**)

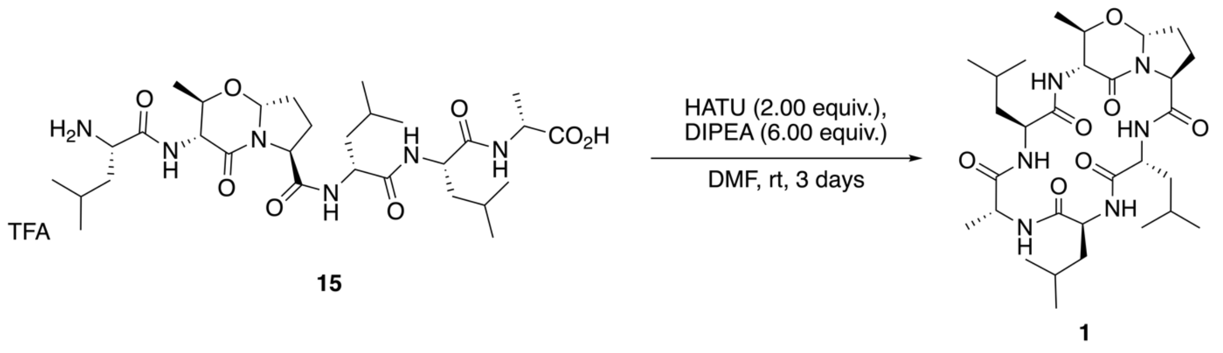

Table S2: ^{1}H NMR signal assignment of Fusahexin (**1**) with TOCSY and HSQC spectrum (600 MHz, 300 K, DMSO-d$_6$).

amino acid	NH / ppm	αH / ppm	βH / ppm	further / ppm
ala^1	7.73	4.35	1.15	
Leu^2	7.98	4.35	1.53	γH: 1.47<br>δH: 0.88, 0.83
ehr^3	7.70	4.28	3.84	γH: 1.24
Pro^4	n.d	4.32	2.36, 1.71	δH: 5.52<br>γH: 2.04, 1.89
leu^5	8.50	4.24	1.57	γH: 1.46<br>δH: 0.87, 0.80
Leu^6	7.63	4.39	1.49	γH: 1.36, 1.29<br>δH: 0.82, 0.80

HRMS (ESI) m/z: $[M + H]^+$ Calcd for $C_{30}H_{50}N_6O_7H$ 607.3814; Found 607.3814.

analytical *rp* HPLC: t_R=11.857 min (0.45 mL/min, 10-100% MeCN in 20 min).

purity detected by *rp*-HPLC: 94%.

TFA.H-Leu-ehr-Hag-leu-Leu-ala-OH (**20**)

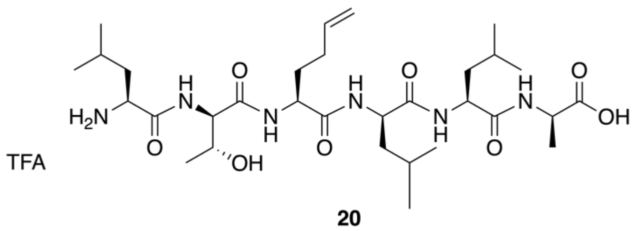

20

Table S3: ^{1}H NMR signal assignment of peptide **20** with TOCSY and HSQC spectrum (600 MHz, 300 K, DMSO-d_6).

amino acid	NH / ppm	αH / ppm	βH / ppm	further / ppm
ala^1	8.05	4.18	1.28	
Leu^2	8.06	3.88	1.52	γH: 1.65<br>δH: 0.89
ehr^3	8.65	4.38	3.81	γH: 1.05
Hag^4	7.97	4.43	1.65	γH: 2.01<br>δH: 5.76<br>εH: 4.95
leu^5	8.20	4.30	1.41	γH: 1.52<br>δH: 0.85
Leu^6	8.31	4.26	1.45	γH: 1.55<br>δH: 0.83

HRMS (ESI) m/z: $[M + H]^+$ Calcd for $C_{30}H_{56}N_6O_8H$ 641.4232; Found 641.4226.

analytical *rp* HPLC: t_R=8.953 min (0.45 mL/min, 10-90% MeCN in 20 min).

purity detected by *rp*-HPLC: 51%.

cyclo(-ala-Leu-ehr-Hag-leu-Leu) (**12**)

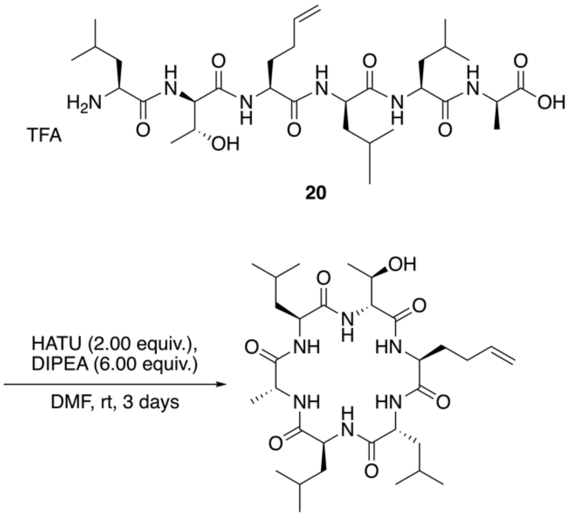

Table S4: ^{1}H NMR signal assignment of peptide **12** with TOCSY and HSQC spectrum (600 MHz, 300 K, DMSO-d$_6$).

amino acid	NH / ppm	αH / ppm	βH / ppm	further / ppm
ala^1	7.87	4.26	1.12	
Leu^2	8.15	4.26	1.43	γH: 1.50<br>δH: 0.83
ehr^3	8.03	4.10	3.80	γH: 1.01<br>OH: 4.69
Hag^4	7.71	4.33	1.65, 1.55	γH: 2.01, 1.92<br>δH: 5.78<br>εH: 4.94
leu^5	8.07	4.33	1.41	γH: 1.50<br>δH: 0.84
Leu^6	8.05	4.27	1.41	γH: 1.50<br>δH: 0.84

HRMS (ESI) m/z: [M + Na]$^+$ Calcd for $C_{31}H_{54}N_6O_7Na$ 645.3946; Found 645.3947.

TFA.H-Leu-ehr=Pro-Leu-Leu-Ala-OH (**16**)

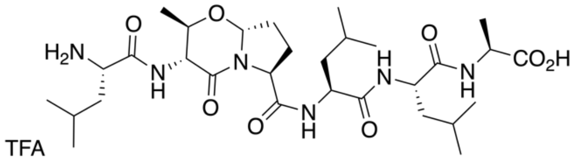

16

Table S5: ^{1}H NMR signal assignment of peptide **16** with TOCSY and HSQC spectrum (600 MHz, 300 K, DMSO-d_6).

amino acid	NH / ppm	αH / ppm	βH / ppm	further / ppm
Ala[1]	8.04	4.17	1.24	
Leu[2]	8.15	3.83	1.57	γH: 1.57<br>δH: 0.81
ehr[3]	8.84	4.65	3.72	γH: 1.24
Pro[4]	n.d	4.57	2.12, 1.83	γH: 2.12, 1.83<br>δH: 5.55
Leu[5]	7.99	4.30	1.43	γH: 1.43<br>δH: 0.86
Leu[6]	7.83	4.27	1.45	γH: 1.45<br>δH: 0.86

HRMS (ESI) m/z: $[M + H]^+$ Calcd for $C_{30}H_{52}N_6O_8H$ 625.3919; Found 625.3900.

rp HPLC: t_R=11.09 min (0.45 mL/min, 10-50% MeCN in 20 min).

semi-preparative *rp* HPLC: 15 mL/min, 20-50% MeCN in 20 min.

purity detected by *rp* HPLC: 97%.

cyclo(-Ala-Leu-ehr=Pro-Leu-Leu) (**10**)

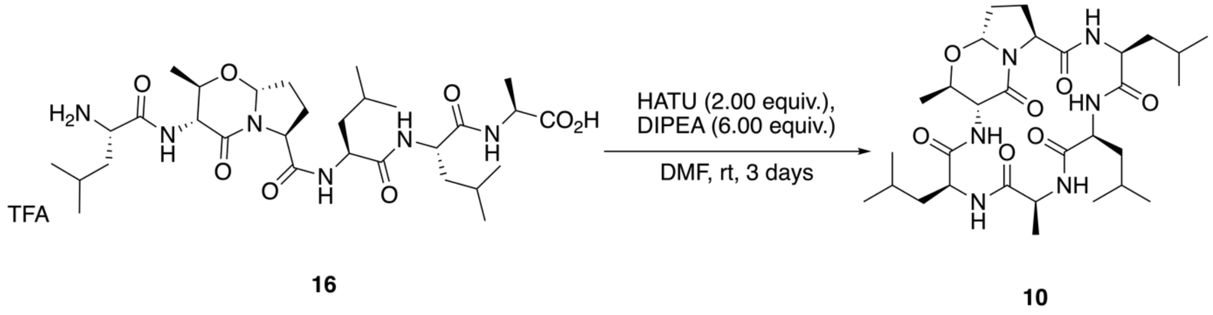

Table S6: ^{1}H NMR signal assignment of peptide **10** with TOCSY and HSQC spectrum (600 MHz, 300 K, DMSO-d$_6$).

amino acid	NH / ppm	αH / ppm	βH / ppm	further / ppm
Ala^1	7.89	4.66	1.14	
Leu^2	7.57	4.00	1.66, 1.57	γH: 1.66<br>δH: 0.94
ehr^3	8.61	4.59	3.72	γH: 1.24
Pro^4	n.d	4.56	2.11, 1.82	γH: 2.11, 1.82<br>δH: 5.53
Leu^5	8.04	4.30	1.45	γH: 1.57<br>δH: 0.88, 0.83
Leu^6	7.93	4.25	1.45	γH: 1.57<br>δH: 0.88, 0.83

HRMS (ESI) m/z: $[M + H]^+$ Calcd for $C_{30}H_{50}N_6O_7H$ 607.3814; Found 607.3814.

rp HPLC: t_R = 9.071 min (0.45 mL/min, 10-90% MeCN in 20 min).

semi-preparative *rp* HPLC: 7 mL/min, 10-90% MeCN in 20 min.

purity detected by *rp* HPLC: 70%.

TFA.H-Leu-ehr=Pro-Leu-leu-Ala-OH (**17**)

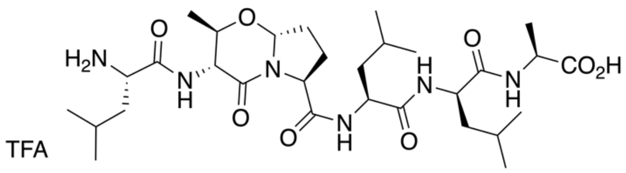

17

Table S7: ^{1}H NMR signal assignment of peptide **17** with TOCSY and HSQC spectrum (600 MHz, 300 K, DMSO-d$_6$).

amino acid	NH / ppm	αH / ppm	βH / ppm	further / ppm
Ala^1	8.02	4.18	1.27	
Leu^2	8.18 (NH_3^+)	3.84	1.58	γH: 1.67<br>δH: 0.91
ehr^3	8.88	4.66	3.73	γH: 1.24
Pro^4	n.d	4.55	2.14, 1.82	γH: 2.14, 1.82<br>δH: 5.54
Leu^5	8.04	4.32	1.46	γH: 1.54<br>δH: 0.88, 0.86
leu^6	8.22	4.27	1.46	γH: 1.56<br>δH: 0.86, 0.81

HRMS (ESI) m/z: $[M + H]^+$ Calcd for $C_{30}H_{52}N_6O_8H$ 625.3919; Found 625.3914.

rp HPLC: t_R = 8.142 min (0.45 mL/min, 10-90% MeCN in 20 min).

semi-preparative *rp* HPLC: 7 mL/min, 10-90% MeCN in 20 min.

purity detected by *rp* HPLC: 81%.

cyclo(-Ala-Leu-ehr=Pro-Leu-leu) (**11**)

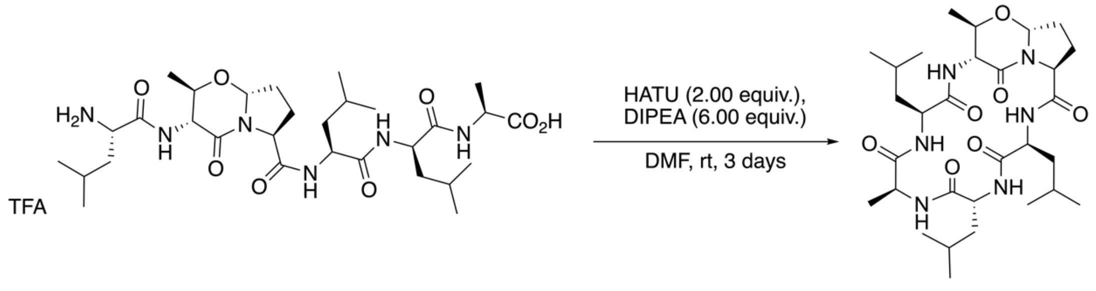

17 **11**

Table S8: ^{1}H NMR signal assignment of peptide **11** with TOCSY and HSQC spectrum (600 MHz, 300 K, DMSO-d$_6$).

amino acid	NH / ppm	αH / ppm	βH / ppm	further / ppm
Ala^1	8.06	4.15	1.20	
Leu^2	7.49	4.17	1.52	γH: 1.52<br>δH: 0.88
ehr^3	8.14	4.61	3.69	γH: 1.27
Pro^4	n.d	4.10	2.30, 1.70	γH: 2.30, 1.65<br>δH: 5.63
Leu^5	7.48	4.37	1.52, 1.41	γH: 1.52<br>δH: 0.88, 0.83
leu^6	7.79	4.27	1.44	γH: 1.51<br>δH: 0.87, 0.82

HRMS (ESI) m/z: $[M + Na]^+$ Calcd for $C_{30}H_{50}N_6O_7Na$ 629.3644; Found 629.3623.

rp HPLC: t_R = 7.514 min (0.45 mL/min, 10-90% MeCN in 20 min).

semi-preparative *rp* HPLC: 7 mL/min, 10-90% MeCN in 20 min.

purity detected by *rp* HPLC: 92%.

TFA.H-ehr=Pro-Phe-Gly-Gly-Gly-OH (**21**)

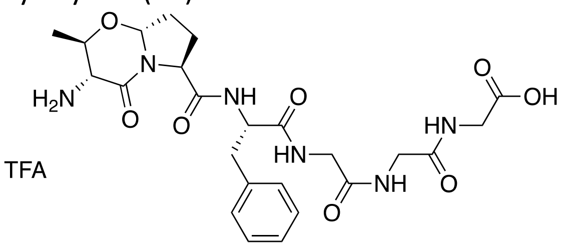

21

Table S9: ^{1}H NMR signal assignment of peptide **21** with TOCSY and HSQC spectrum (600 MHz, 300 K, DMSO-d_6).

amino acid	NH / ppm	αH / ppm	βH / ppm	further / ppm
ehr[1]	n.d	4.04	3.93	γH: 1.41
Pro[2]	n.d	4.56	1.92, 1.70	γH: 2.04, 1.70<br>δH: 5.35
Phe[3]	8.11	4.61	3.06, 2.82	$H_{arom.}$: 7.25
Gly[4]	8.26	3.75		
Gly[5]	8.19	3.76		
Gly[6]	8.11	3.75		

HRMS (ESI) m/z: $[M + Na + H]^{2+}$ Calcd for $C_{24}H_{32}N_6O_8NaH$ 556.2203; Found 556.2192.

rp HPLC: t_R = 4.545 min (0.45 mL/min, 10-90% MeCN in 20 min).

semi-preparative *rp* HPLC: 7 mL/min, 10-90% MeCN in 20 min.

purity detected by *rp* HPLC: 81%.

<u>*cyclo*(-ehr=Pro-Phe-Gly-Gly-Gly) (**13**)</u>

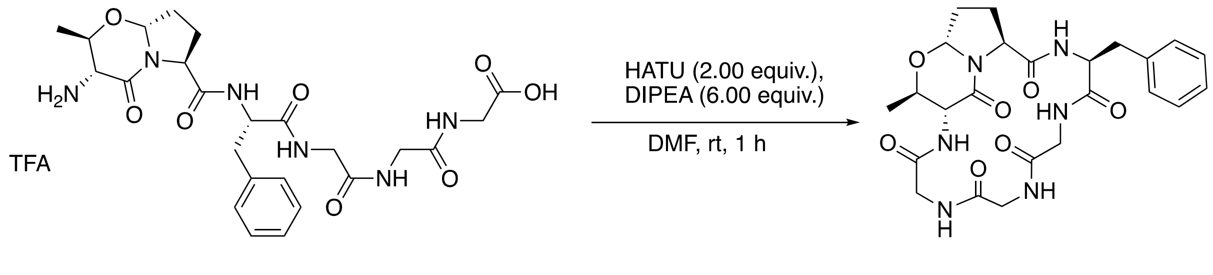

21 **13**

Table S10: ^{1}H NMR signal assignment of peptide **13** with TOCSY and HSQC spectrum (600 MHz, 300 K, DMSO-d$_6$).

amino acid	NH / ppm	αH / ppm	βH / ppm	further / ppm
ehr[1]	7.60	4.29	4.06	γH: 1.32
Pro[2]	n.d	4.07	2.23, 1.68	γH: 2.01, 1.27<br>δH: 5.50
Phe[3]	8.20	4.47	3.30, 2.78	H2,6: 7.19<br>H3,5: 7.25<br>H4: 7.17
Gly[4]	7.37	4.15, 3.61		
Gly[5]	8.39	3.79, 3.60		
Gly[6]	8.27	3.77, 3.63		

HRMS (ESI) m/z: $[M + Na]^+$ Calcd for $C_{24}H_{30}N_6O_7Na$ 537.2068; Found 537.2051.

rp HPLC: t_R = 6.618 min (0.45 mL/min, 10-90% MeCN in 20 min).

semi-preparative *rp* HPLC: 7 mL/min, 10-90% MeCN in 20 min.

purity detected by *rp* HPLC: 91%.

<u>*cyclo*(-ehr-Hag-Phe-Gly-Gly-Gly) (**18**)</u>

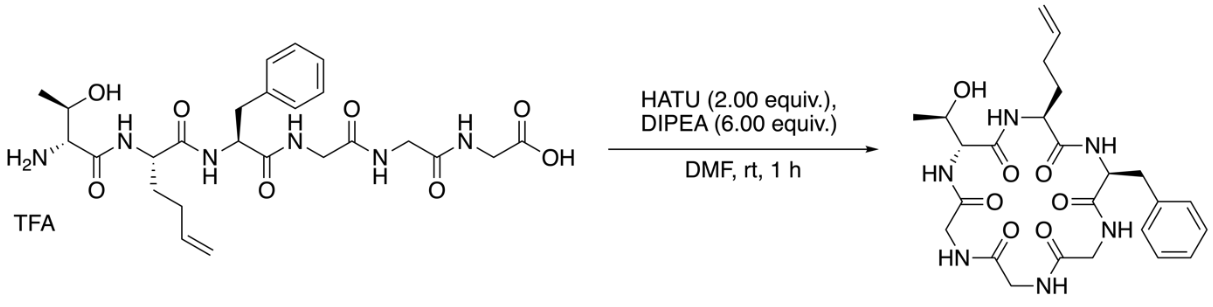

22

18

Table S11: ^{1}H NMR signal assignment of peptide **18** with TOCSY and HSQC spectrum (600 MHz, 300 K, DMSO-d_6).

amino acid	NH / ppm	αH / ppm	βH / ppm	further / ppm
ehr^{1}	8.06	4.10	3.84	γH: 1.07<br>OH: 4.85
Hag^{2}	8.35	4.00	1.63, 1.49	γH: 1.93<br>δH: 5.72<br>εH: 4.95
Phe^{3}	7.53	4.53	3.20, 2.93	H_{arom}: 7.25, 7.19
Gly^{4}	8.80	3.70, 3.51		
Gly^{5}	8.18	3.74, 3.64		
Gly^{6}	7.45	3.76		

HRMS (ESI) m/z: $[M + Na]^+$ Calcd for $C_{25}H_{34}N_6O_7Na$ 553.2381; Found: 553.2371.

rp HPLC: t_R = 16.206 min (0.45 mL/min, 1-30% MeCN in 20 min).

semi-preparative *rp* HPLC: 15 mL/min, 1-30% MeCN in 20 min.

purity detected by *rp* HPLC: 95%.

Table S12 Fusahexin and cyclic analogs together with the linear precursors. According to the original description of Fusahexin, we start the numbering at alanine. All α-amino acids are shown in the three-letter-code with the first letter capitalized for L-configuration and small letters for D-amino acids. A double line indicated a bicyclic lactam bridge instead of the usual amide bond (single line).

TFA.H-Leu-**ehr=Pro**-leu-Leu-ala-OH	(**15**)	lin. Fusahexin
cyclo(-ala-Leu-**ehr=Pro**-leu-Leu)	(**1**)	Fusahexin
TFA.H-Leu-**ehr-Hag**-leu-Leu-ala-OH	(**20**)	
cyclo(-ala-Leu-**ehr-Hag**-leu-Leu)	(**12**)	
TFA.H-Leu-ehr=Pro-**Leu**-Leu-**Ala**-OH	(**16**)	
cyclo(-**Ala**-Leu-ehr=Pro-**Leu**-Leu)	(**10**)	
TFA.H-Leu-ehr=Pro-**Leu-leu**-Ala-OH	(**17**)	
cyclo(-Ala-Leu-ehr=Pro-**Leu-leu**)	(**11**)	
TFA.H-**ehr=Pro**-Phe-Gly-Gly-Gly)	(**21**)	
cyclo(-**ehr=Pro**-Phe-Gly-Gly-Gly)	(**13**)	

2. Analytical data

Fmoc-ehr-OH (**6**)

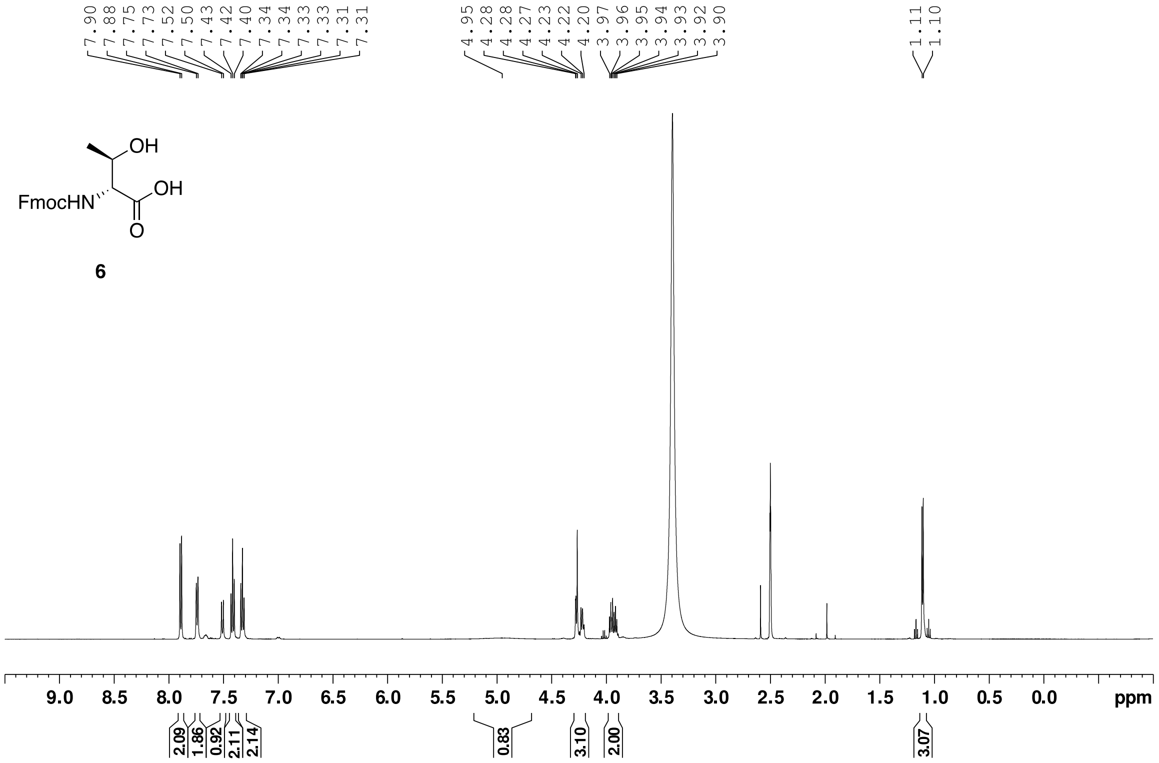

Figure S1: ^{1}H NMR spectrum of Fmoc-ehr-OH (**6**) (500 MHz, 300 K, DMSO-d_6).

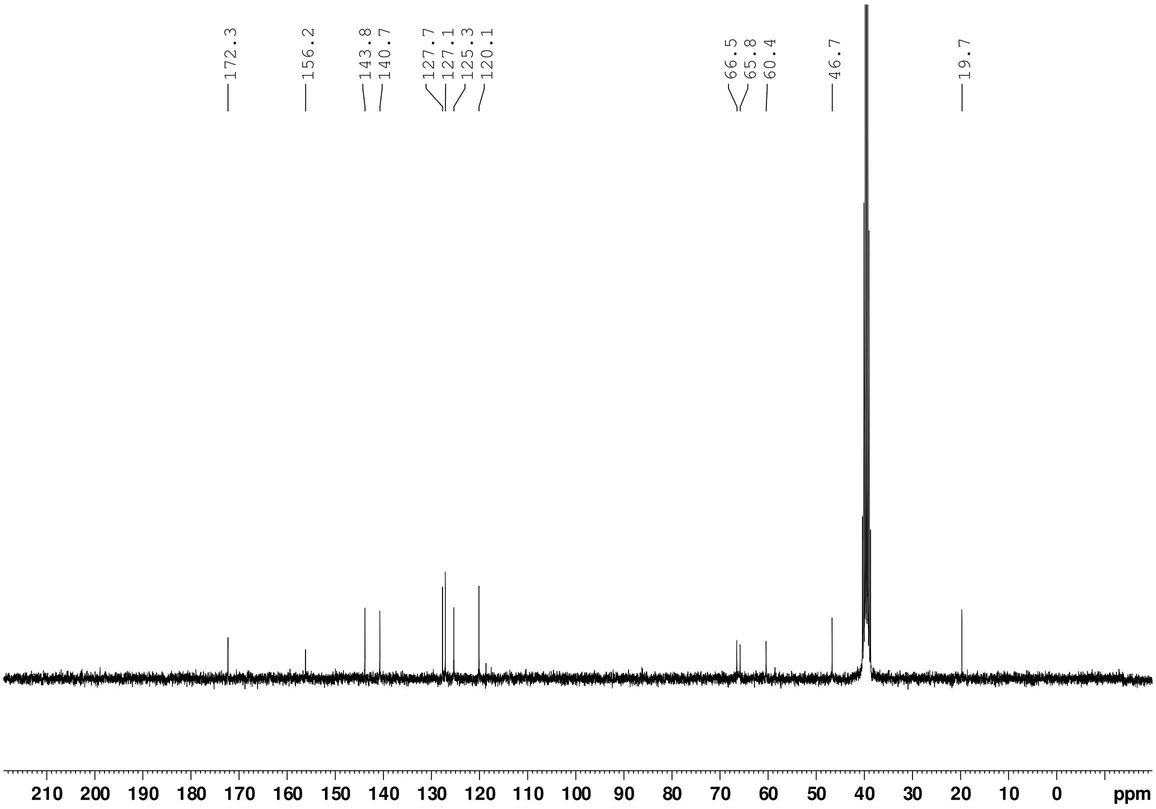

Figure S2: ^{13}C{^{1}H} NMR spectrum of Fmoc-ehr-OH (**6**) (75 MHz, 300 K, DMSO-d_6).

Boc-Hag-OMe (**4**)

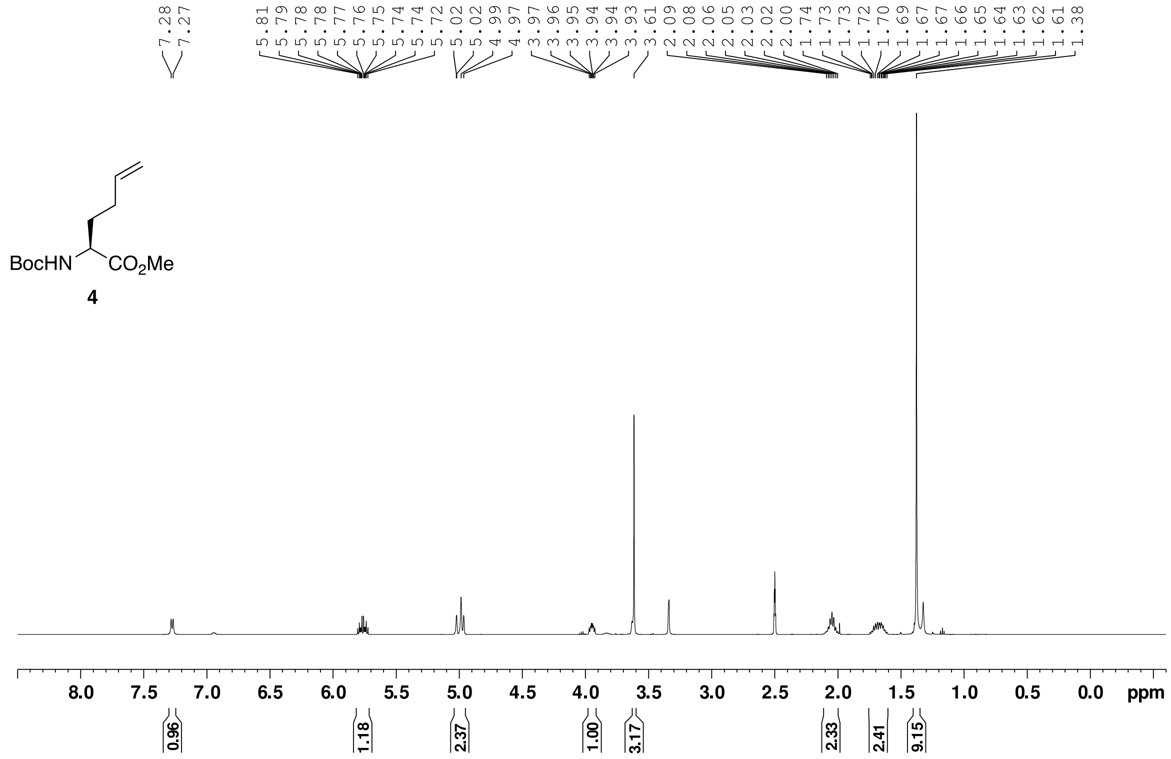

Figure S3: ^{1}H NMR spectrum of Boc-Hag-OMe (**4**) (500 MHz, 300 K, DMSO-d_6).

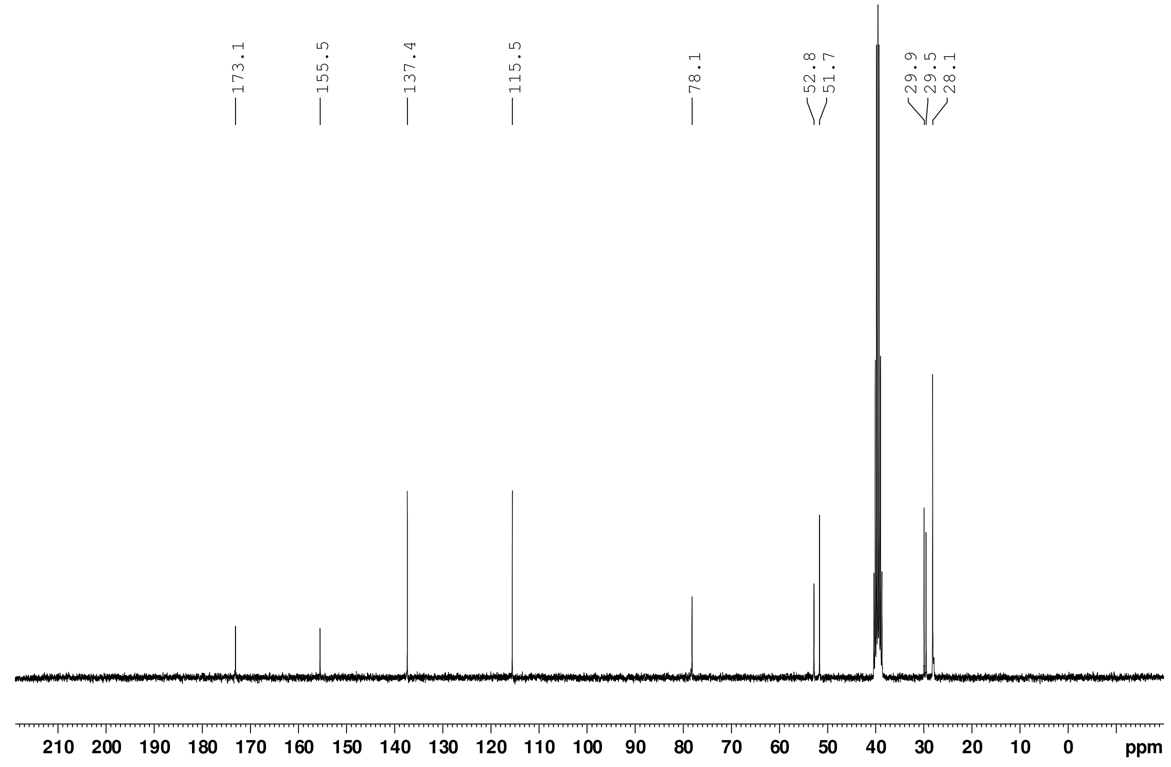

Figure S4: $^{13}C\{^{1}H\}$ NMR spectrum of Boc-Hag-OMe (**4**) (75 MHz, 300 K, DMSO-d_6).

H-Hag-OMe x HCl (**5**)

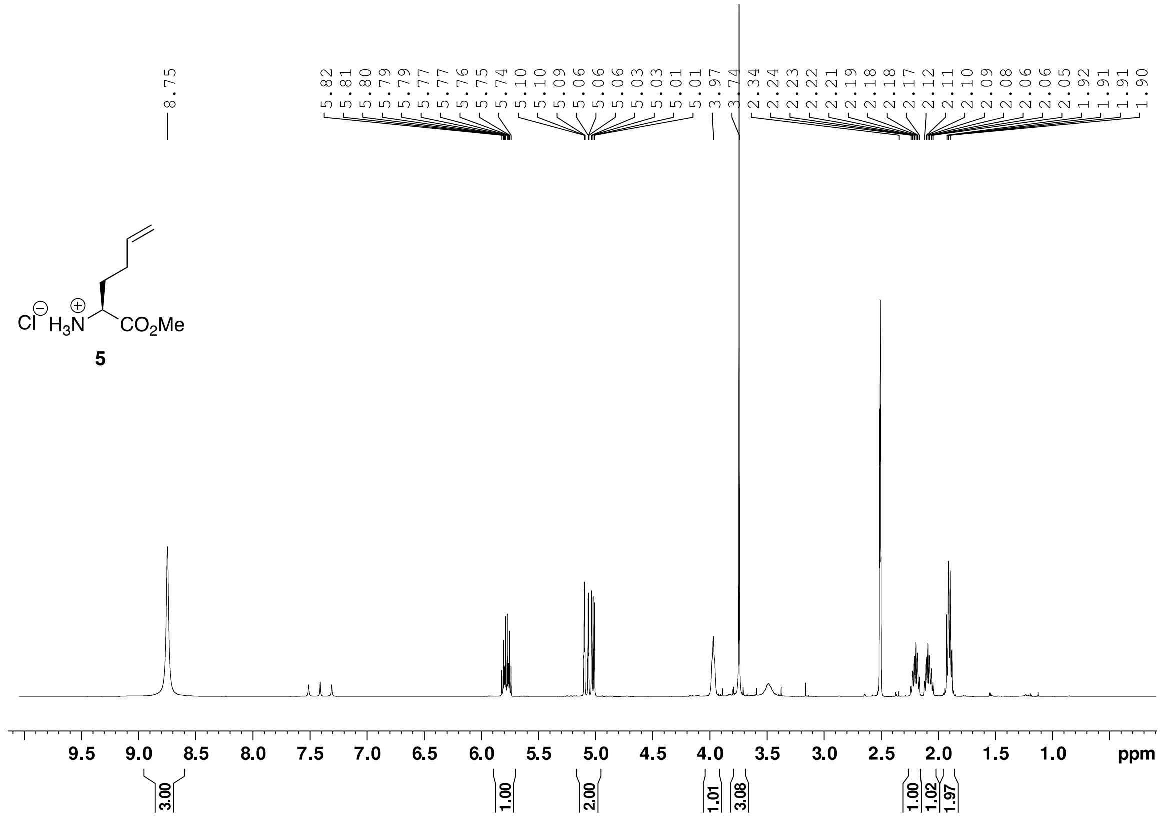

Figure S5: ^{1}H NMR spectrum of H-Hag-OMe x HCl (**5**) (500 MHz, 300 K, DMSO-d_6).

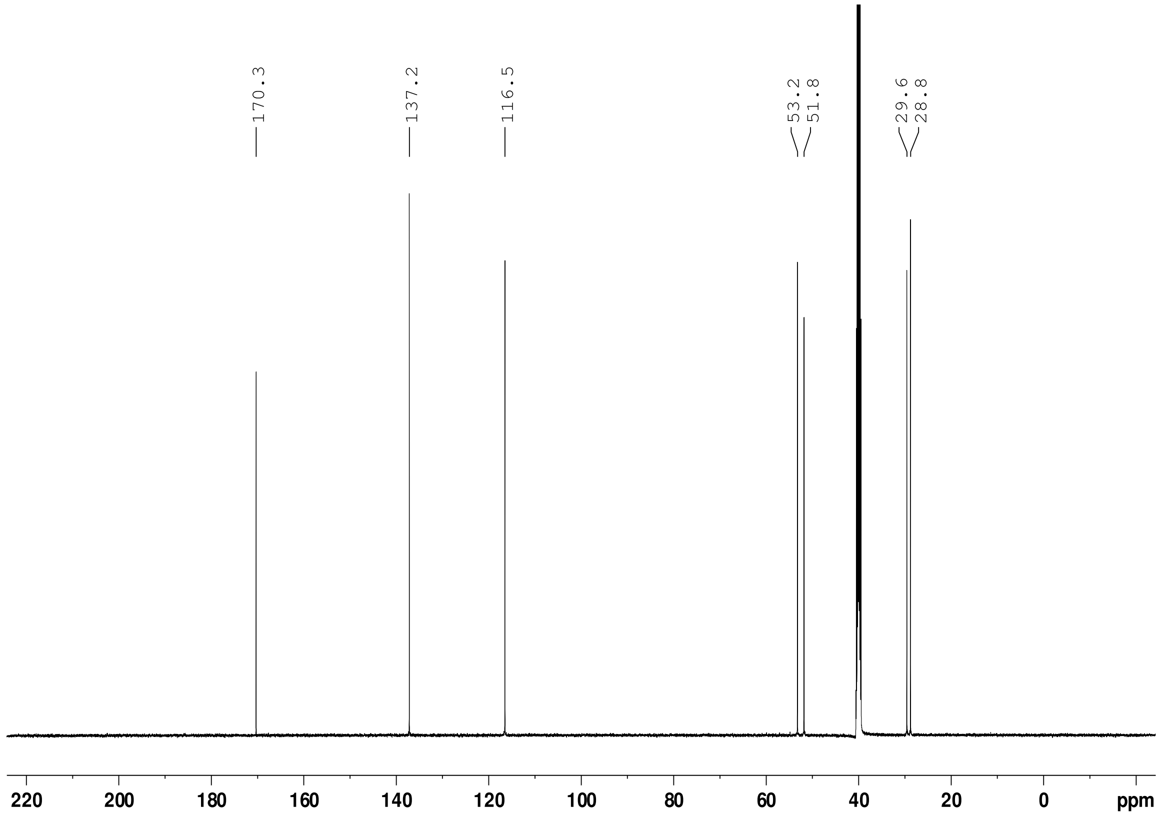

Figure S6: $^{13}C\{^{1}H\}$ NMR spectrum of H-Hag-OMe x HCl (**5**) (125 MHz, 300 K, DMSO-d_6).

Fmoc-her-Hag-OMe (**7**)

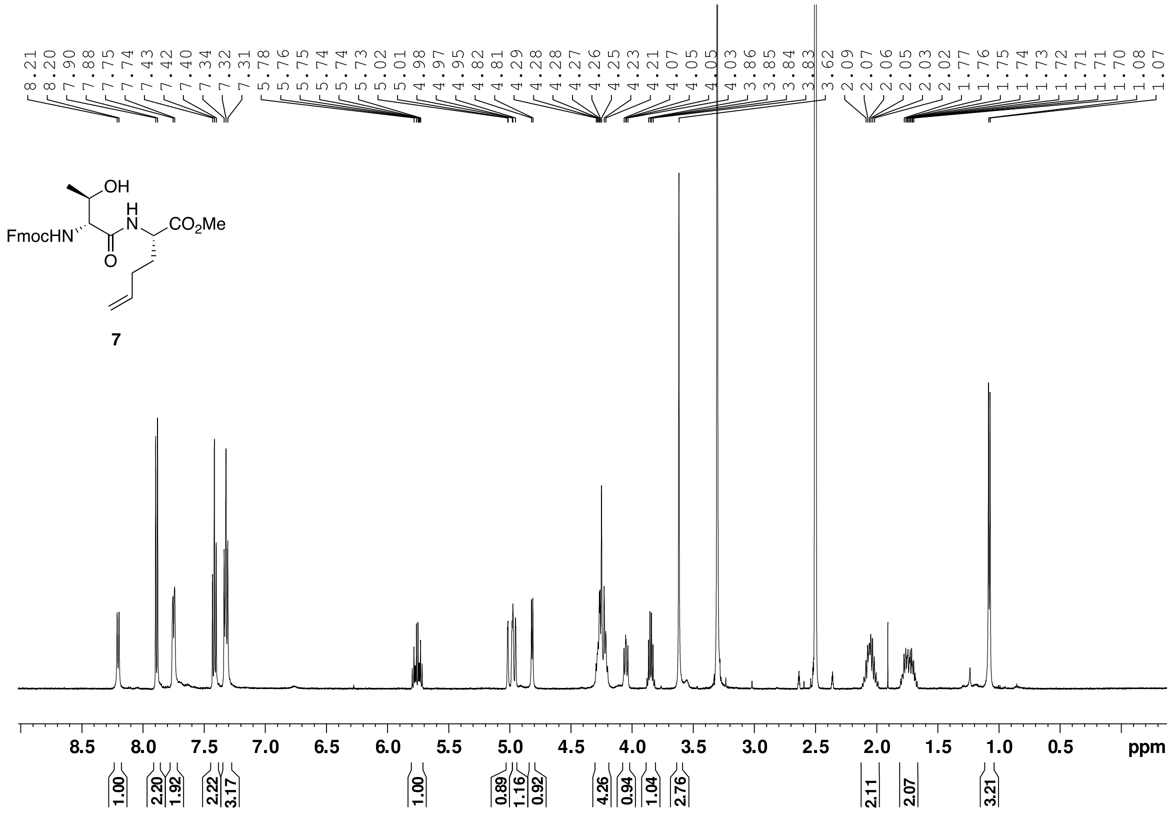

Figure S7: ^{1}H NMR spectrum of Fmoc-ehr-Hag-OMe (**7**) (500 MHz, 300 K, DMSO-d$_6$).

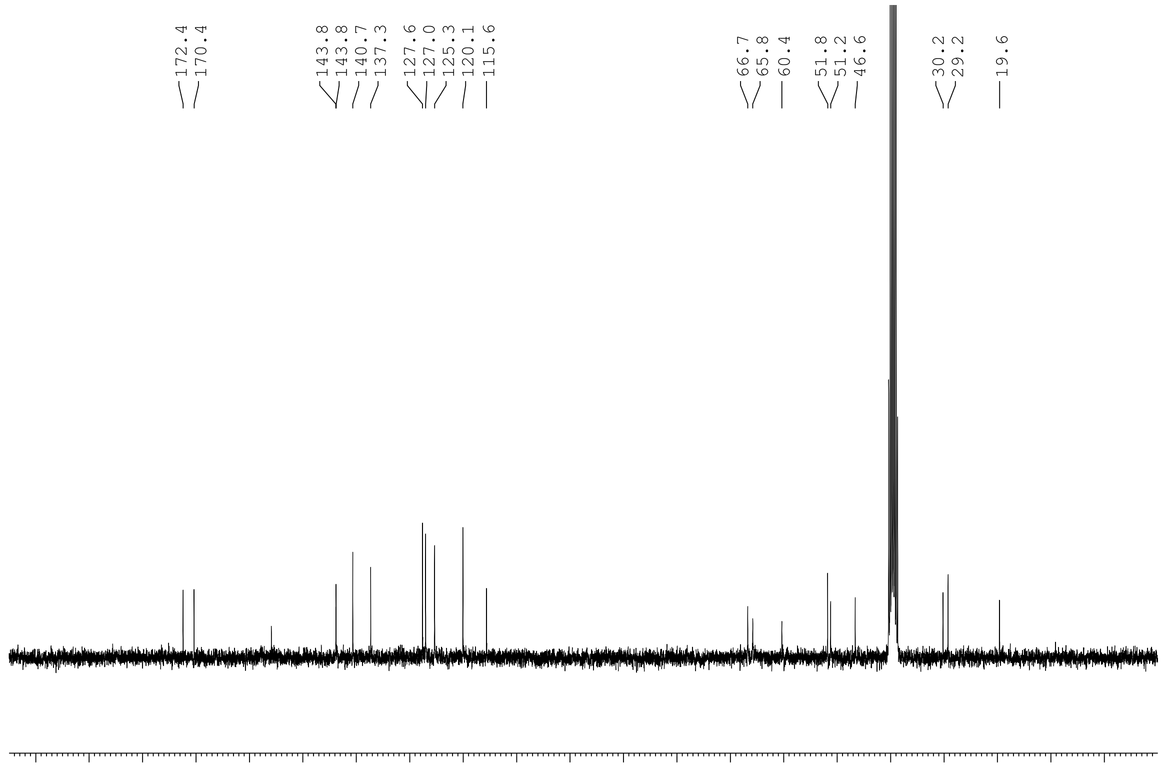

Figure S8: $^{13}C\{^{1}H\}$ NMR spectrum of Fmoc-ehr-Hag-OMe (**7**) (75 MHz, 300 K, DMSO-d$_6$).

Fmoc-ehr=Pro-OMe (**9**)

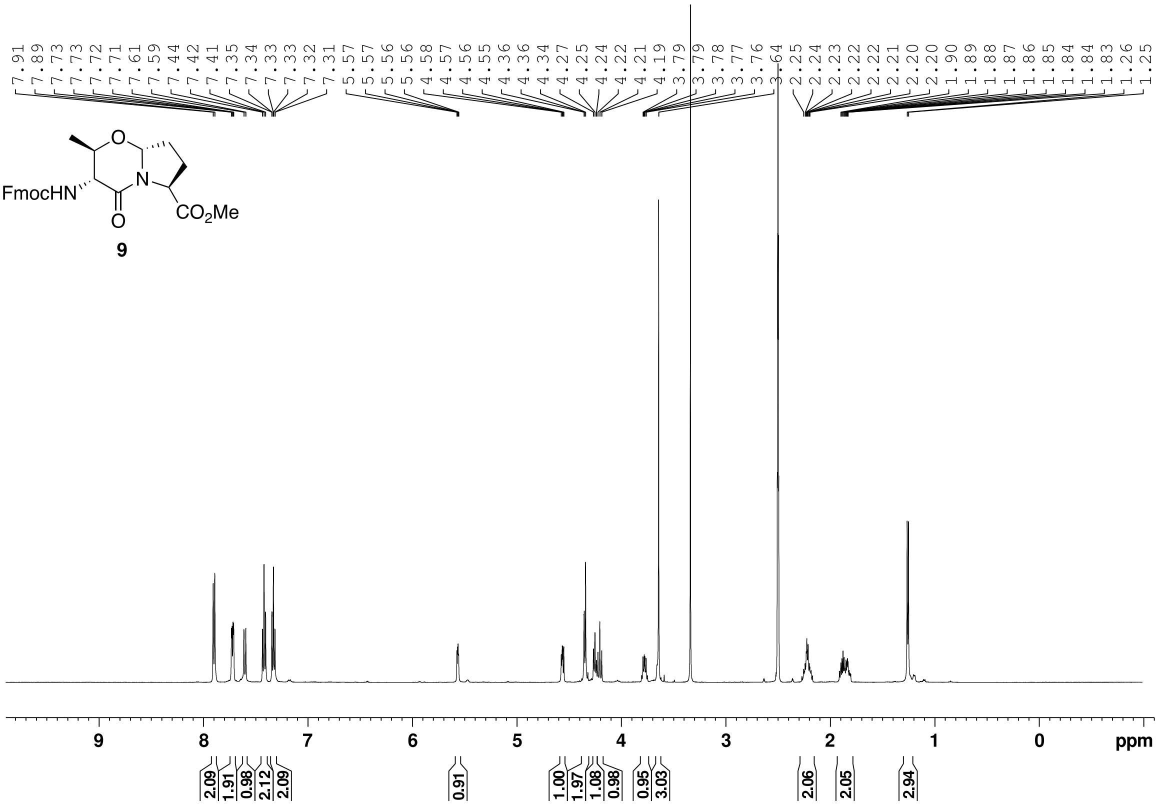

Figure S9: ^{1}H NMR spectrum of Fmoc-ehr=Pro-OMe (**9**) (500 MHz, 300 K, DMSO-d_6.)

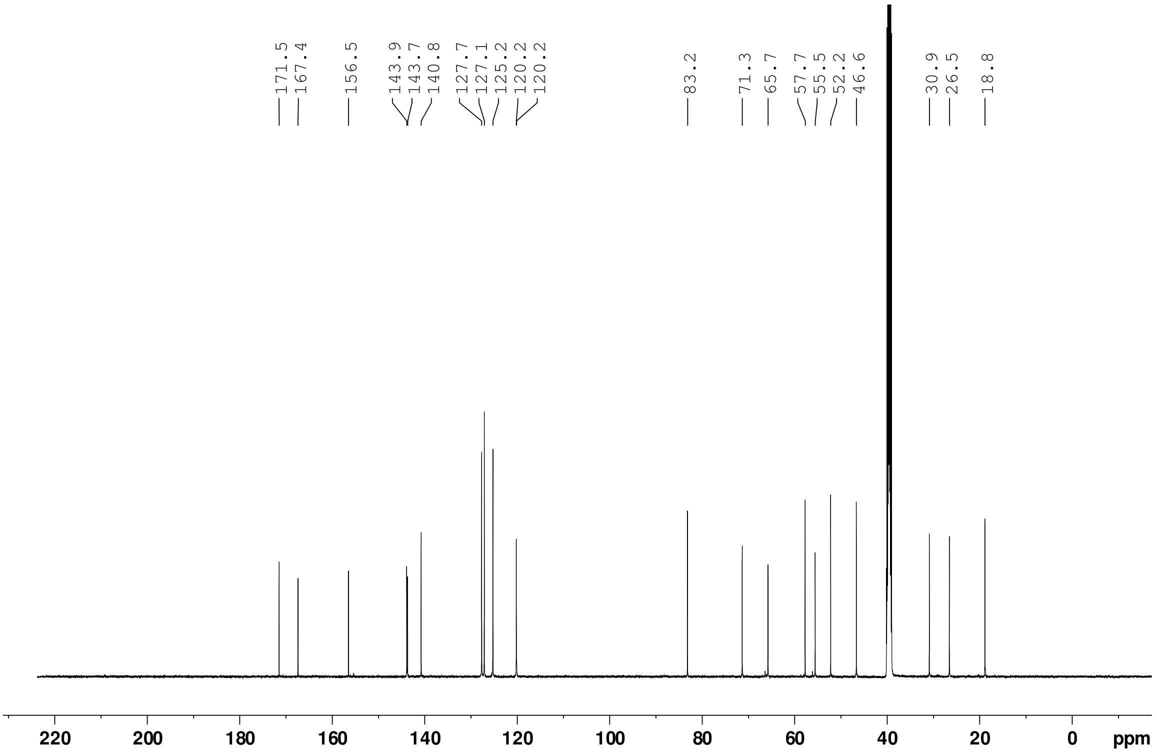

Figure S10: $^{13}C\{^{1}H\}$ NMR spectrum of Fmoc-ehr=Pro-OMe (**9**) (125 MHz, 300 K, DMSO-d_6).

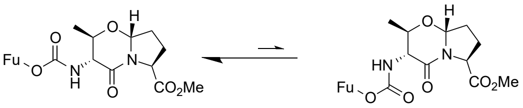

trans **9** *cis* **9**

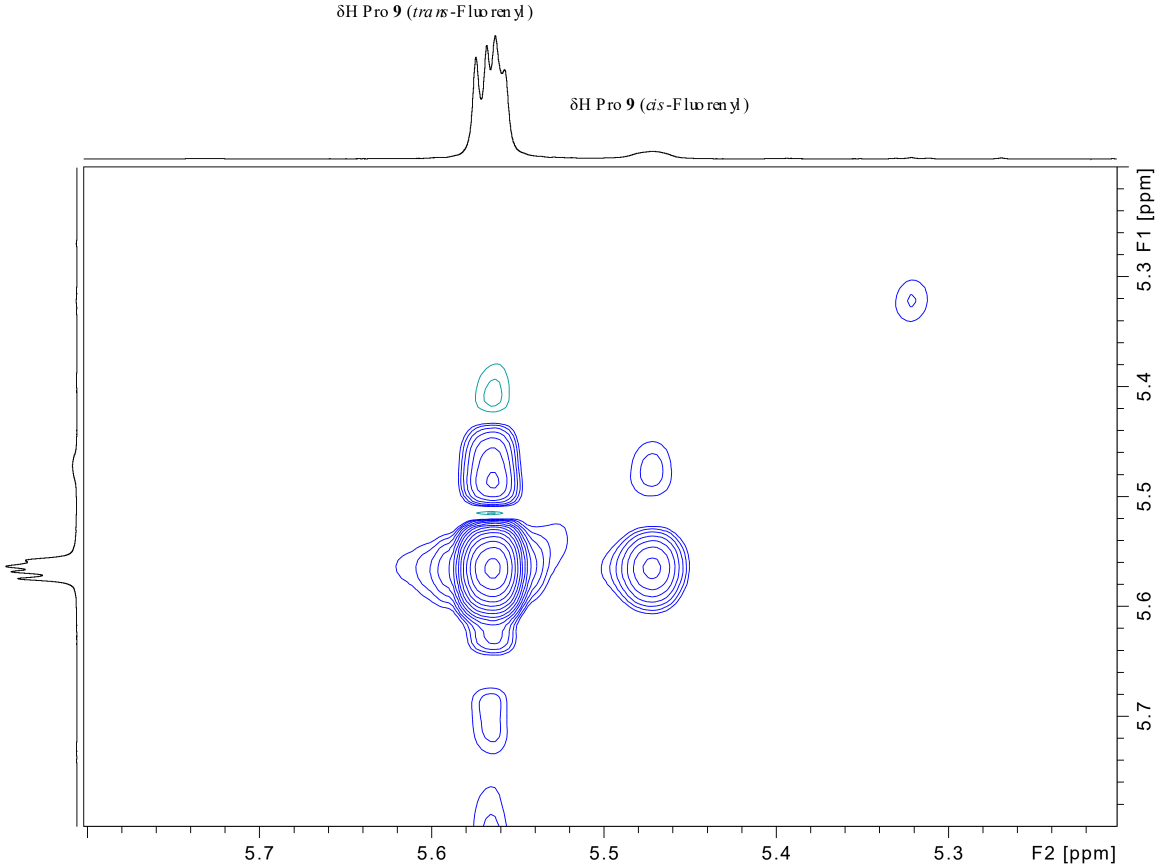

Figure S11: The section in this ROESY spectrum shows that both the *trans* isomer and the *cis* isomer are present in Fmoc-ehr=Pro-OMe (**9**).

Fmoc-ehr=Pro-OH (**2**)

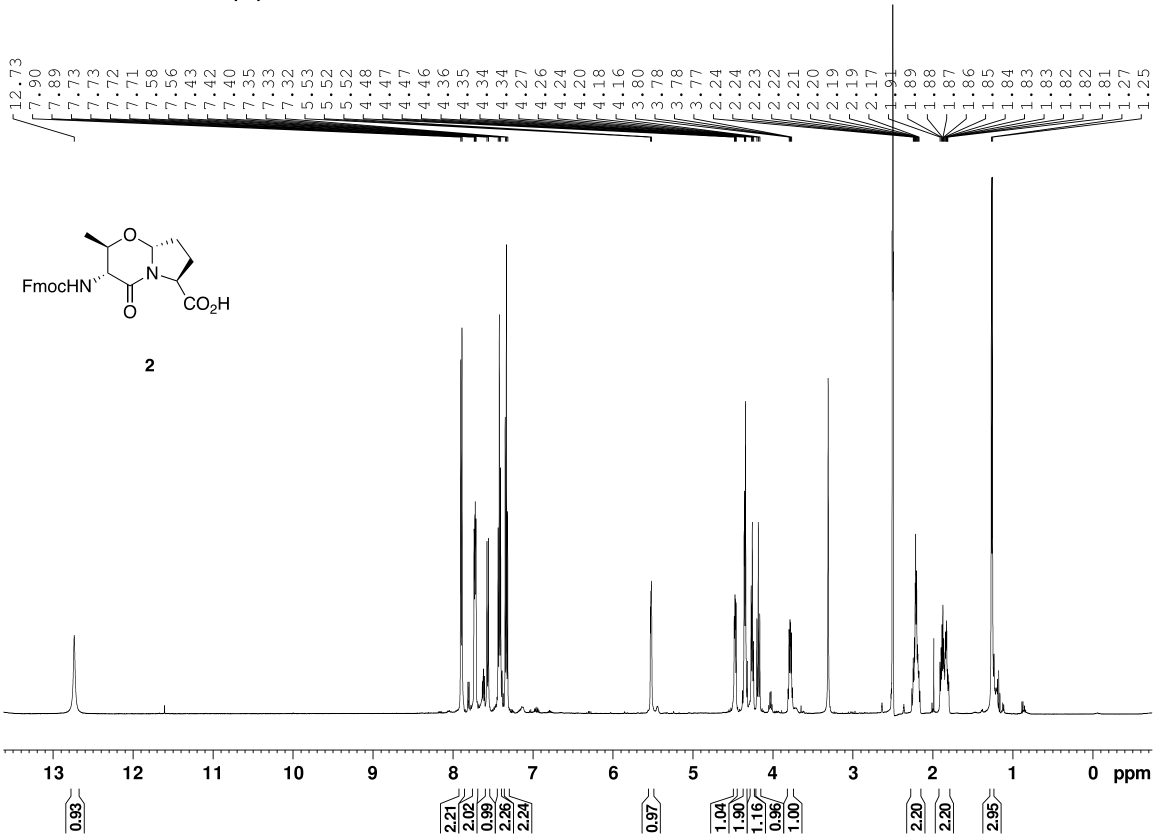

Figure S12: ^{1}H NMR spectrum of Fmoc-ehr=Pro-OH (**2**) (500 MHz, 300 K, DMSO-d$_6$).

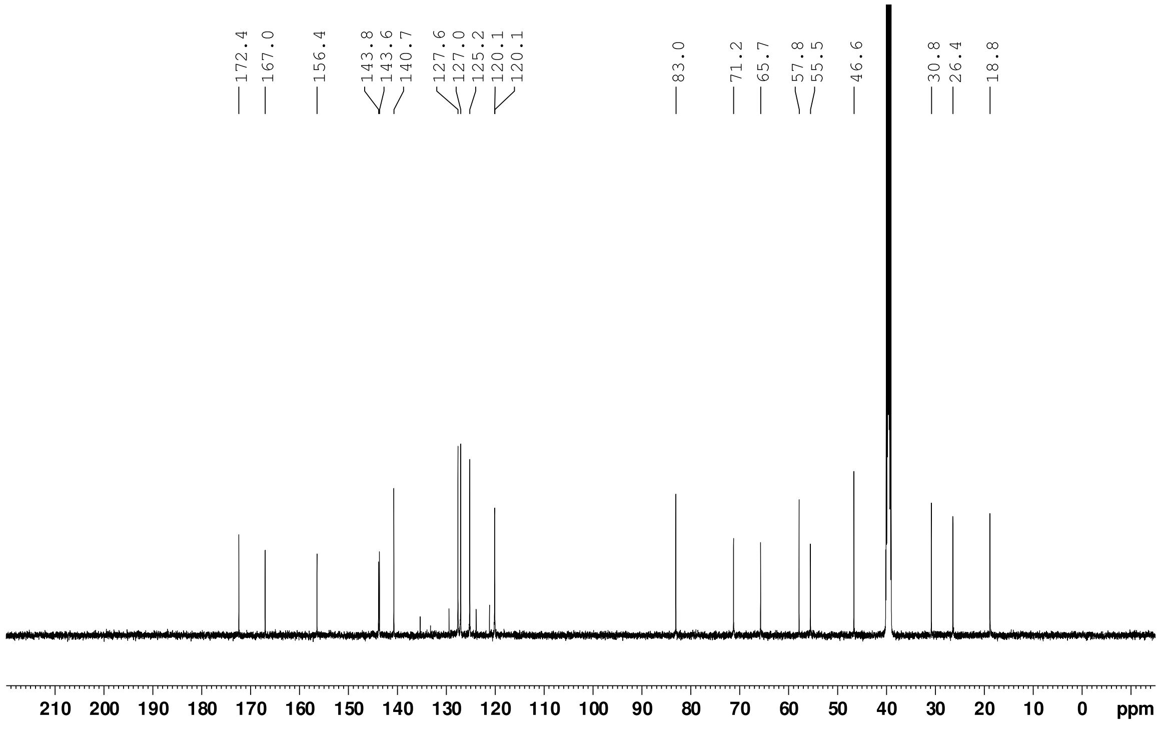

Figure S13: ^{13}C{^{1}H} NMR spectrum of Fmoc-ehr=Pro-OH (**2**) (125 MHz, 300 K, DMSO-d$_6$).

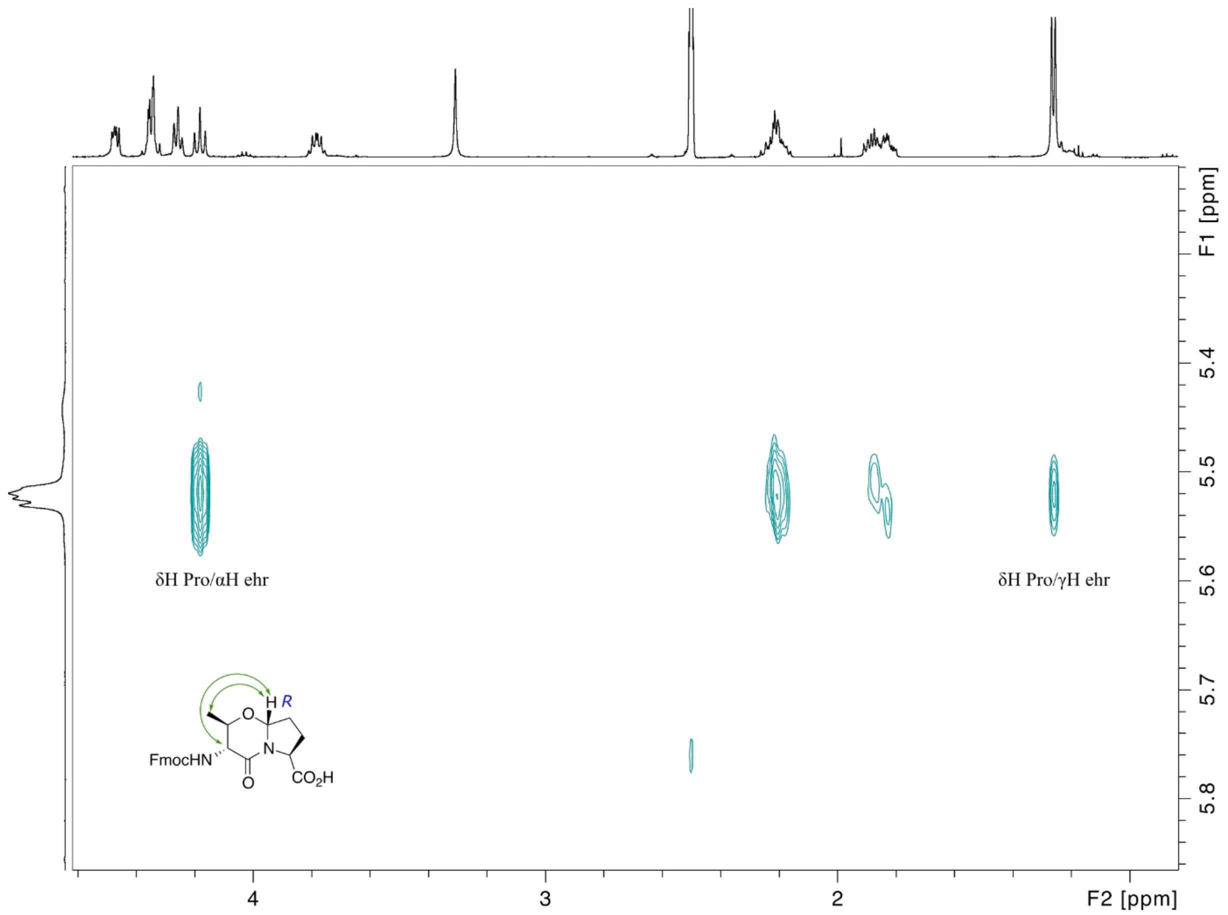

Figure S14: Section of the ROESY spectrum of dipeptide **2**, where important contacts are highlighted to confirm the configuration on the δH Pro as *R*.

TFA.H-Leu-ehr=Pro-leu-Leu-ala-OH (**15**)

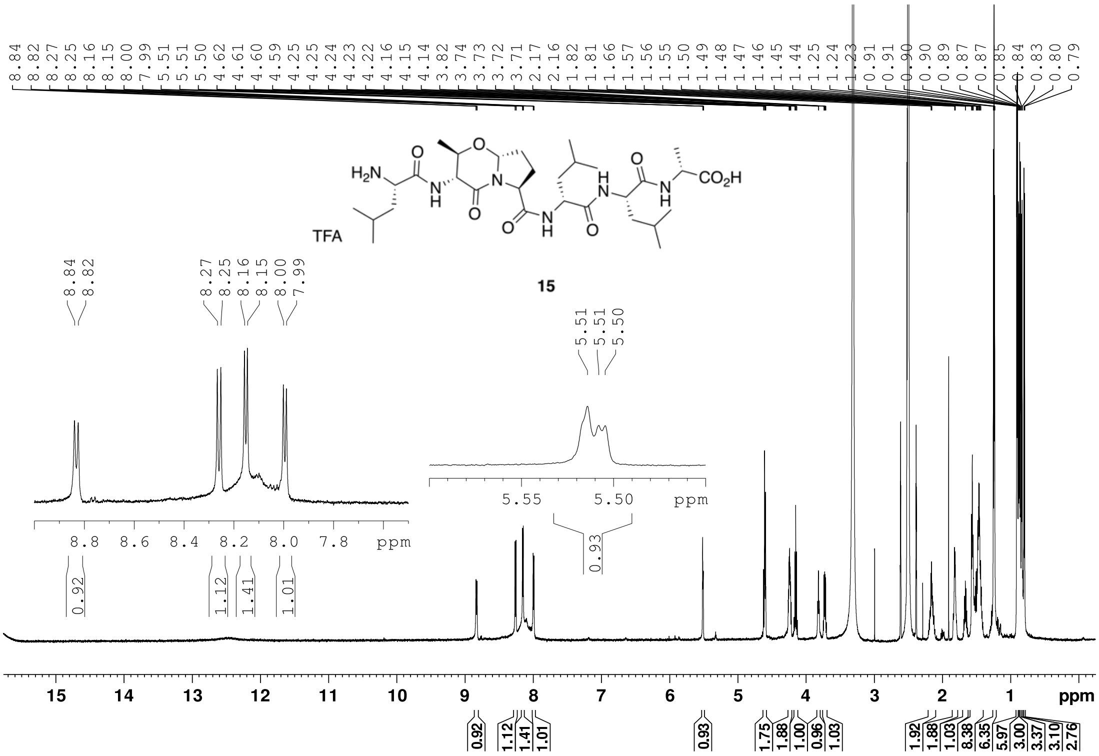

Figure S15: ^{1}H NMR spectrum of the peptide **15** with enlargements of the amide region and the δH Pro (600 MHz, 300 K, DMSO-d$_6$).

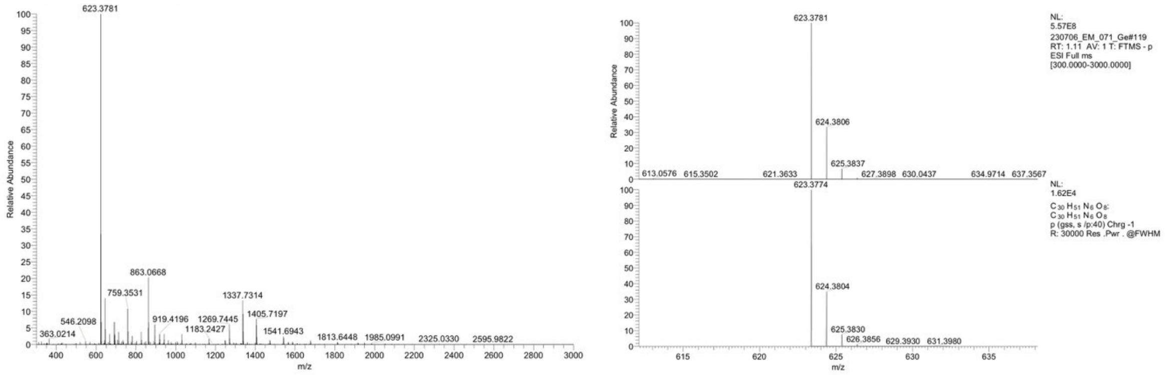

Figure S16: left: ESI+ mass spectrum of peptide **15**; right: high resolution mass spectrum of peptide **15**.

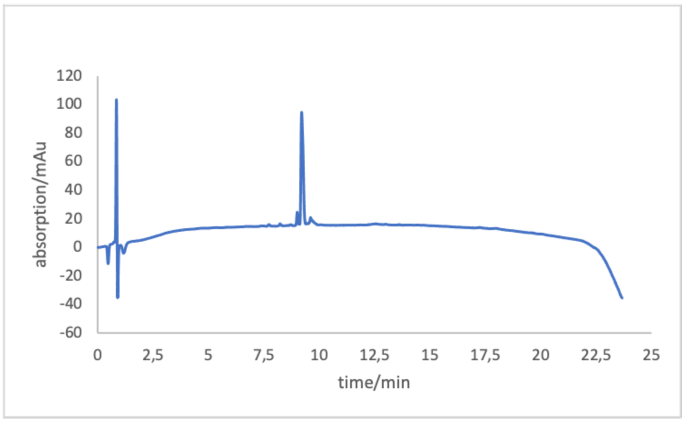

Figure S17: *rp* HPLC chromatogram of peptide **15** (0.45 mL/min, 15-65% MeCN in 20 min, 215 nm).

cyclo(-ala-Leu-ehr=Pro-leu-Leu) (**1**, Fusahexin)

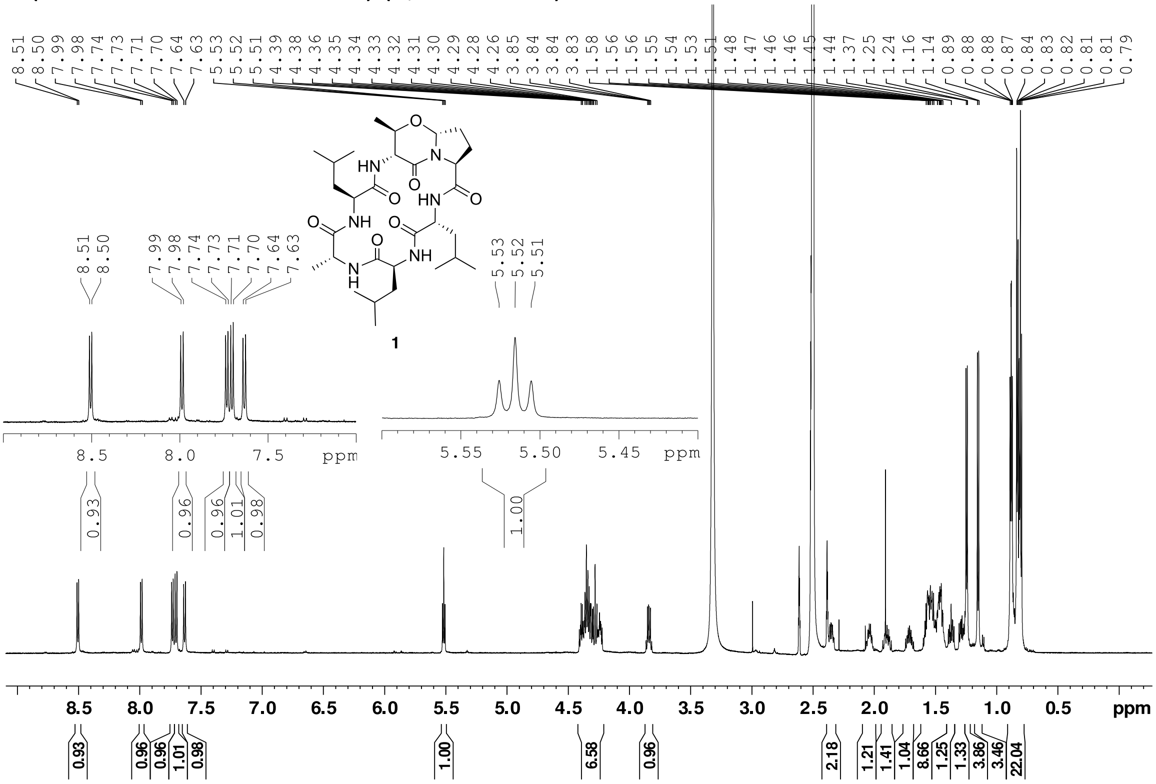

Figure S18: ^{1}H NMR spectrum of peptide **1** (Fusahexin) with enlargements of the amide region and the δH Pro (600 MHz, 300 K, DMSO-d$_6$).

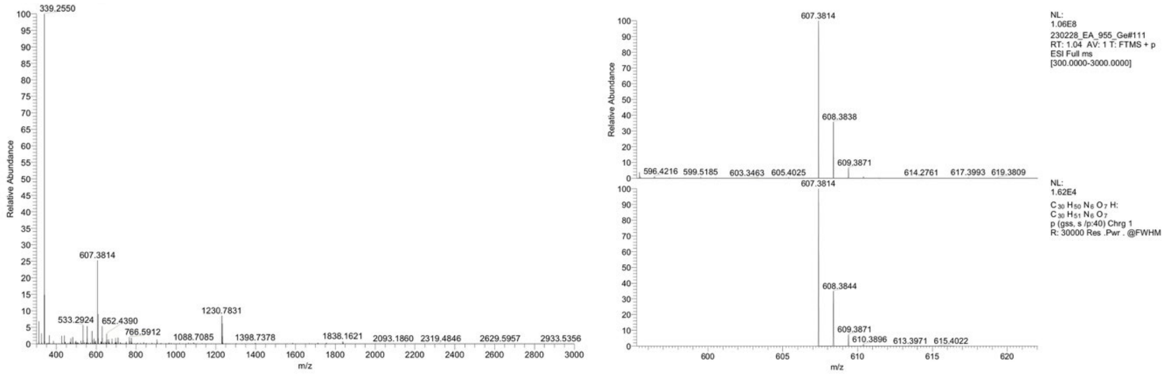

Figure S19: left: ESI+ mass spectrum of peptide **1** (Fusahexin); right: high resolution mass spectrum of peptide **1** (Fusahexin).

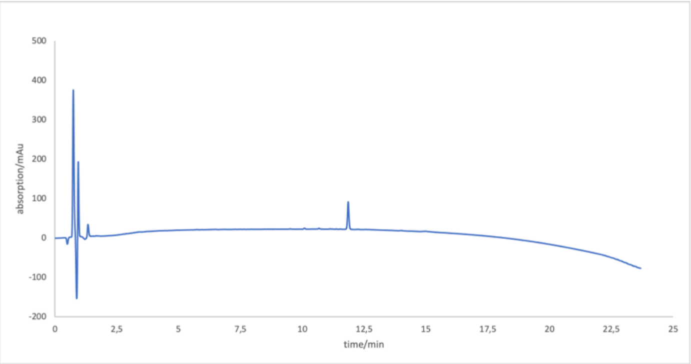

Figure S20: *rp* HPLC chromatogram of peptide **1** (0.45 mL/min, 10-100% MeCN in 20 min, 215 nm).

TFA.Leu-ehr-Hag-leu-Leu-ala-OH (**20**)

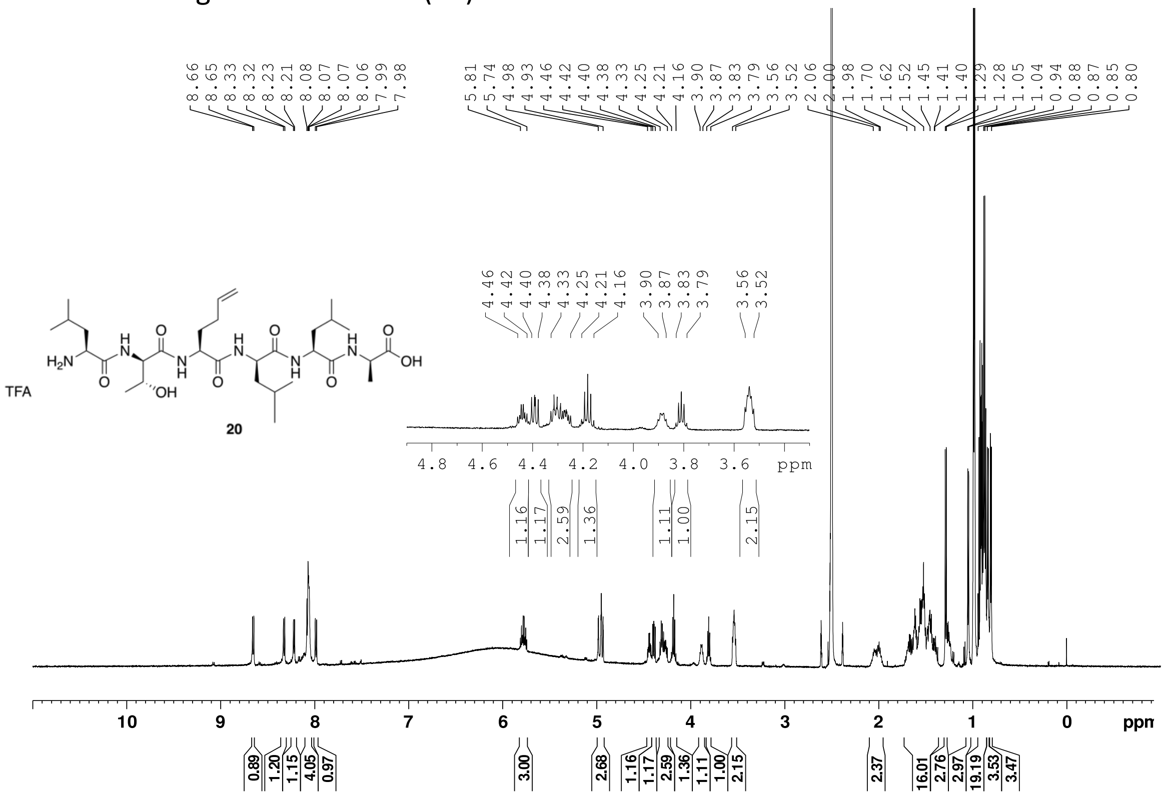

Figure S21: ^{1}H NMR spectrum of peptide **20** with enlargements of the αH region (600 MHz, 300 K, DMSO-d$_6$).

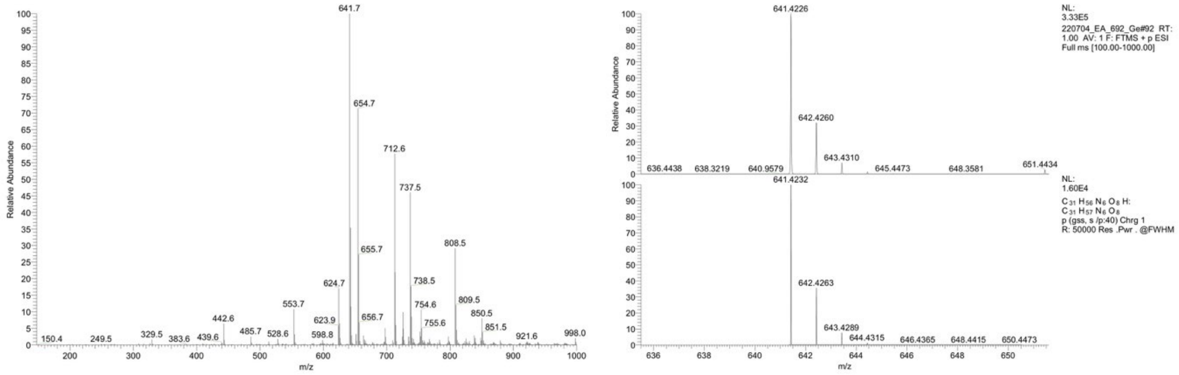

Figure S22: left: ESI+ mass spectrum of peptide **20**; right: high resolution mass spectrum of peptide **20**.

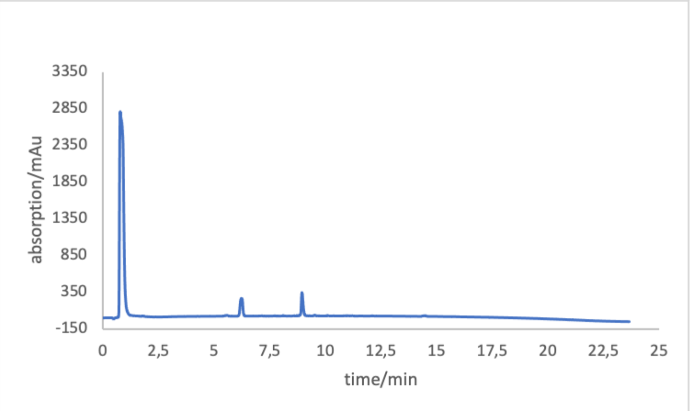

Figure S23: *rp* HPLC chromatogram of peptide **20** (0.45 mL/min, 10-90% MeCN in 20 min, 215 nm).

cyclo(-ala-Leu-ehr-Hag-leu-Leu) (**12**)

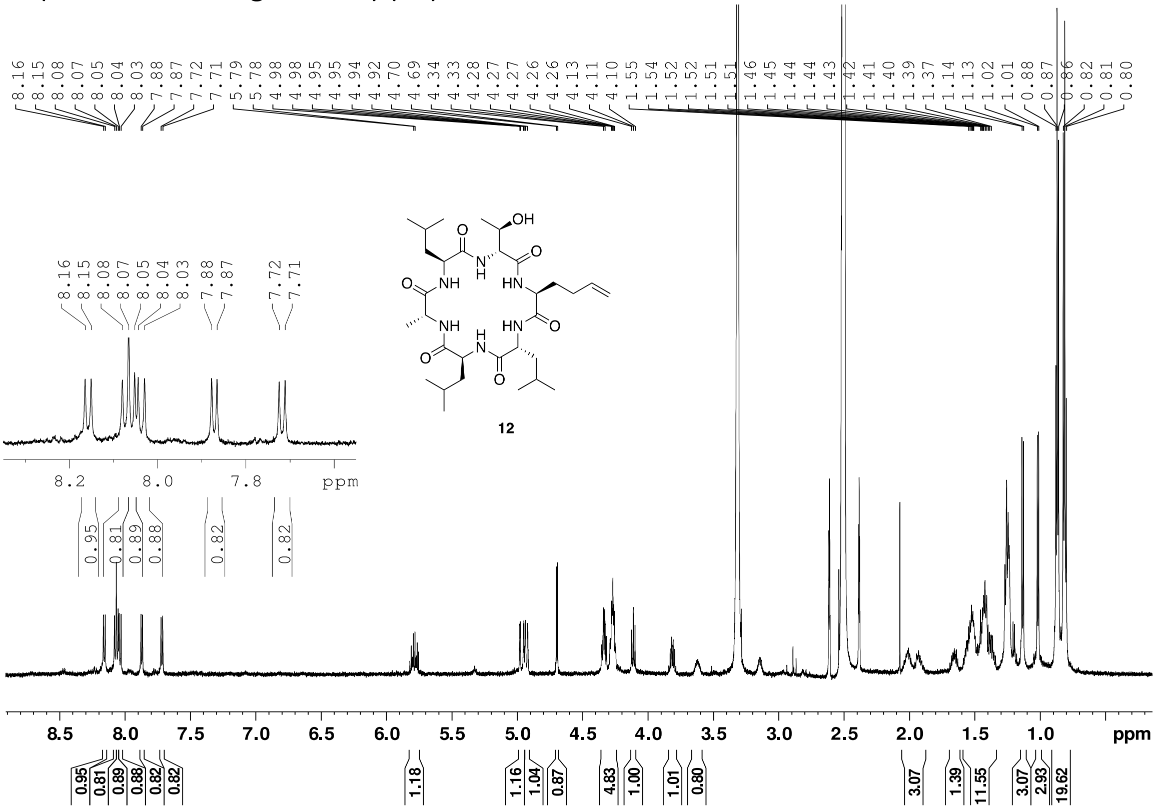

Figure S24: 1H NMR spectrum of peptide **12** with enlargements of the amide region (600 MHz, 300 K, DMSO-d_6).

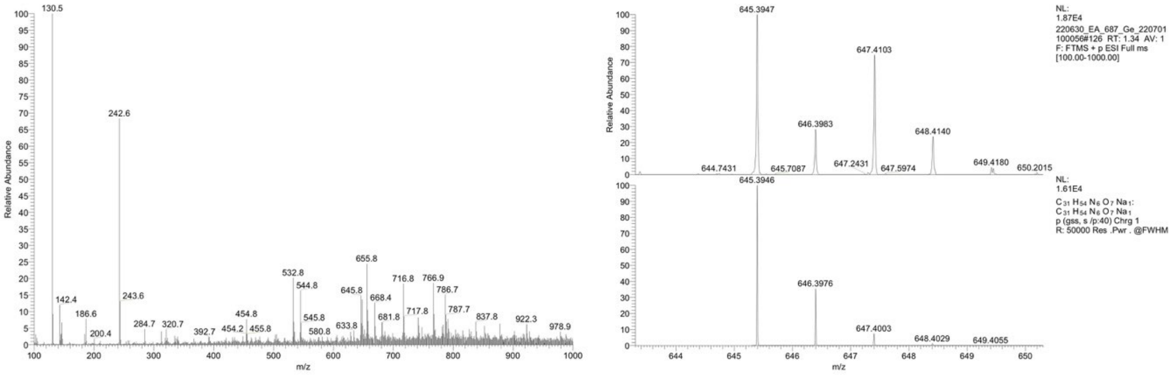

Figure S25: left: ESI+ mass spectrum of peptide **12**; right: high resolution mass spectrum of peptide **12**.

TFA.H-Leu-ehr=Pro-Leu-Leu-Ala-OH (**16**)

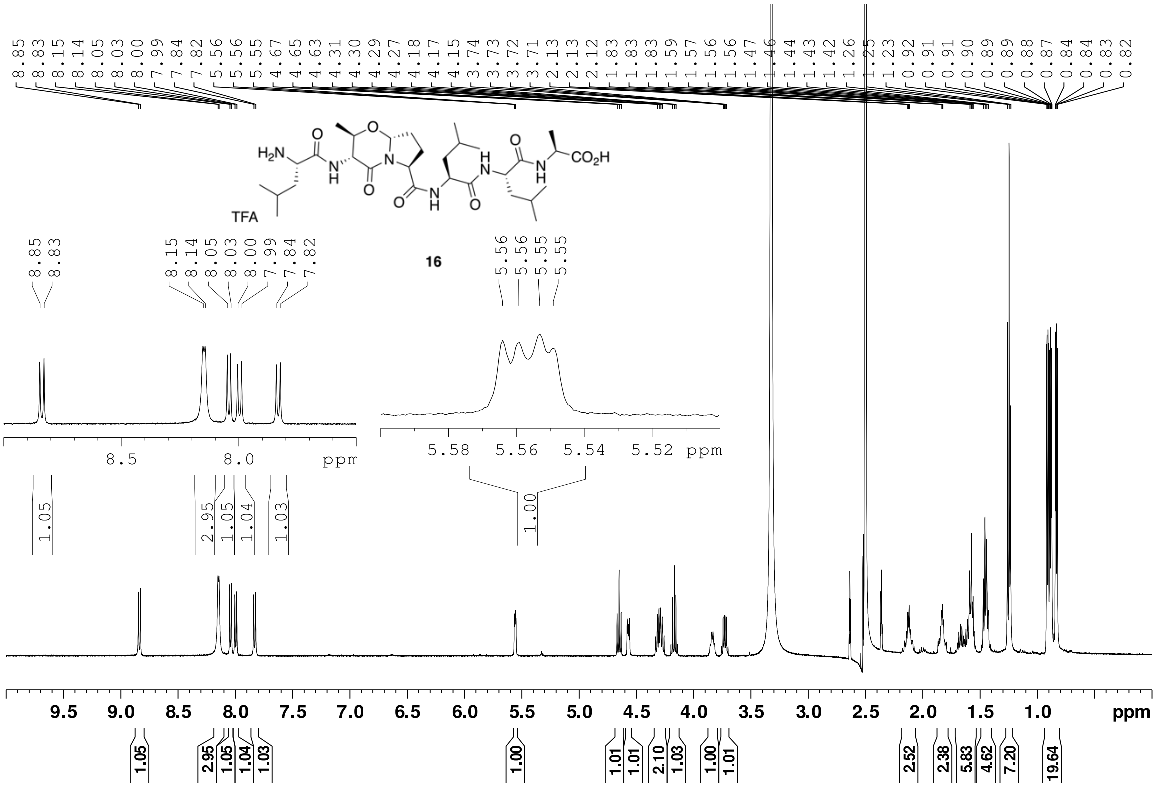

Figure S26: ^{1}H NMR spectrum of peptide **16** with enlargements of the amide region and the δH Pro (600 MHz, 300 K, DMSO-d_6).

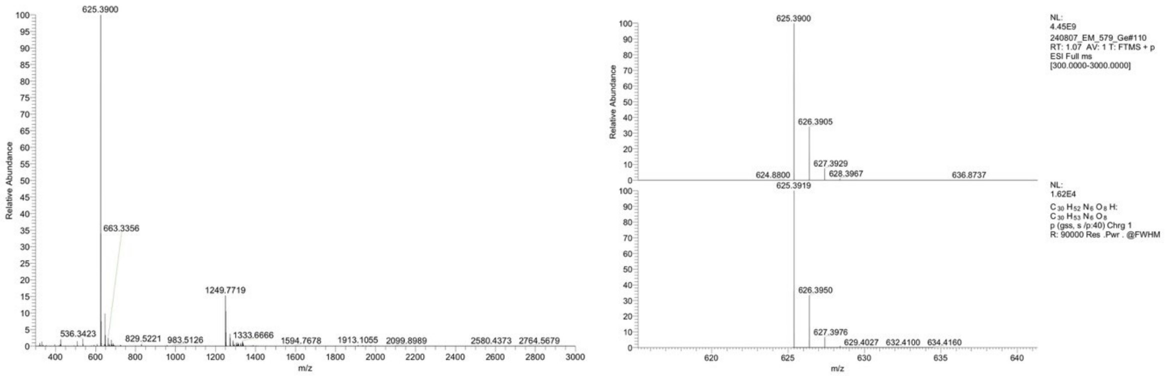

Figure S27: left: ESI+ mass spectrum of peptide **16**; right: high resolution mass spectrum of peptide **16**.

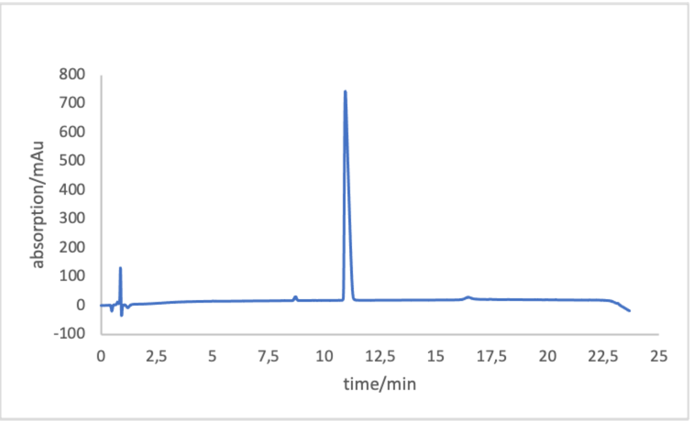

Figure S28: *rp* HPLC chromatogram of peptide **16** (0.45 mL/min, 10-50% MeCN in 20 min, 215 nm).

cyclo(-Ala-Leu-ehr=Pro-Leu-Leu) (**10**)

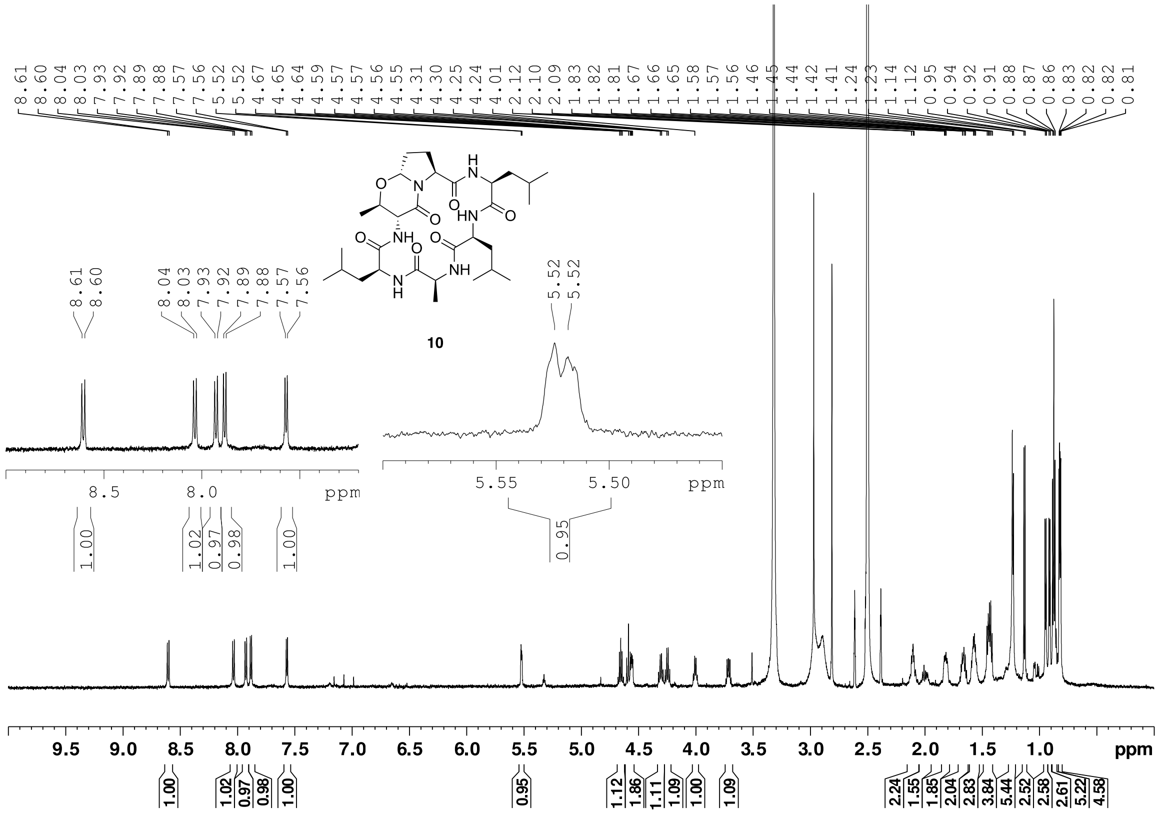

Figure S29: ^{1}H NMR spectrum of peptide **10** with enlargements of the amide region and the δH Pro (600 MHz, 300 K, DMSO-d$_6$).

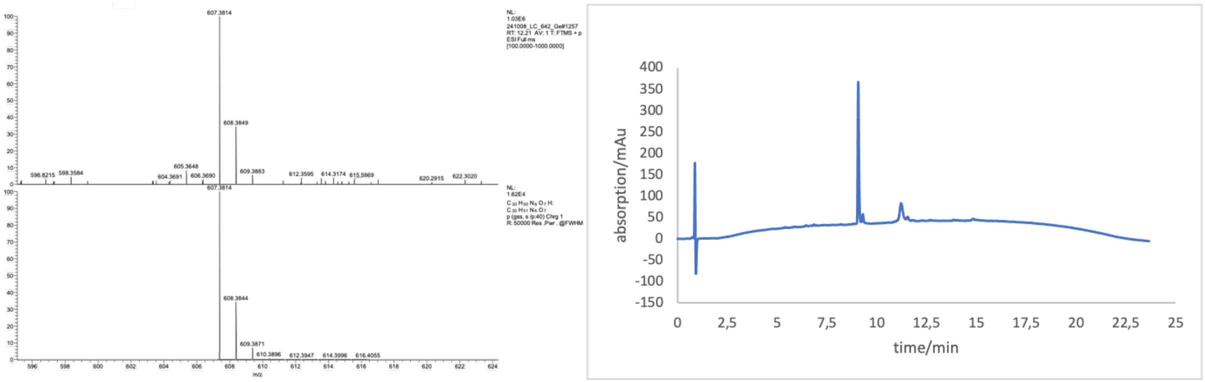

Figure 30: left: high resolution mass spectrum of peptide **10**; right: *rp* HPLC chromatogram of peptide **10** (0.45 mL/min, 10-90 % MeCN in 20 min, 215 nm).

TFA.H-Leu-ehr=Pro-Leu-leu-Ala-OH (**17**)

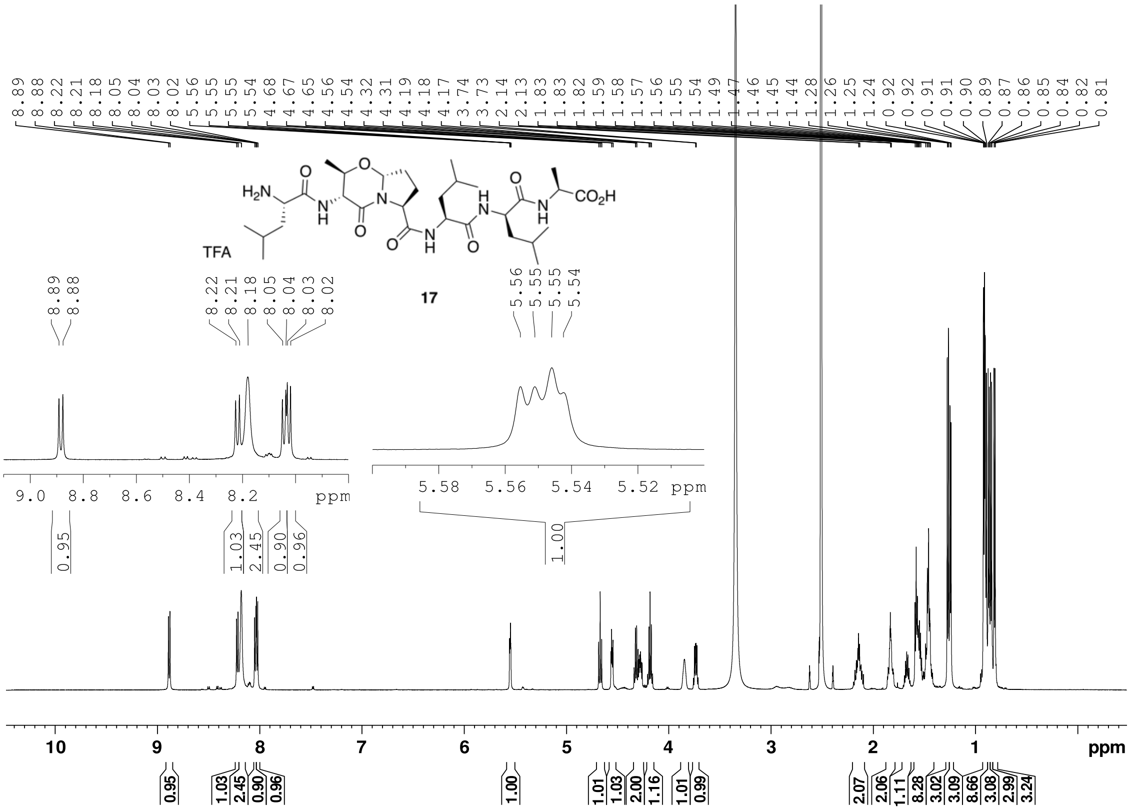

Figure 31: ^{1}H NMR spectrum of peptide **17** with enlargements of the amide region and the δH Pro (600 MHz, 300 K, DMSO-d$_6$).

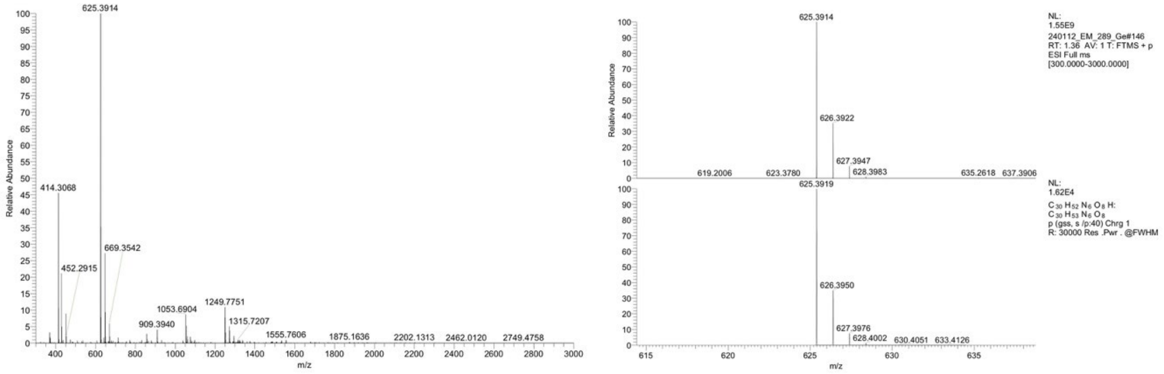

Figure S32: left: ESI+ mass spectrum of peptide **17**; right: high resolution mass spectrum of peptide **17**.

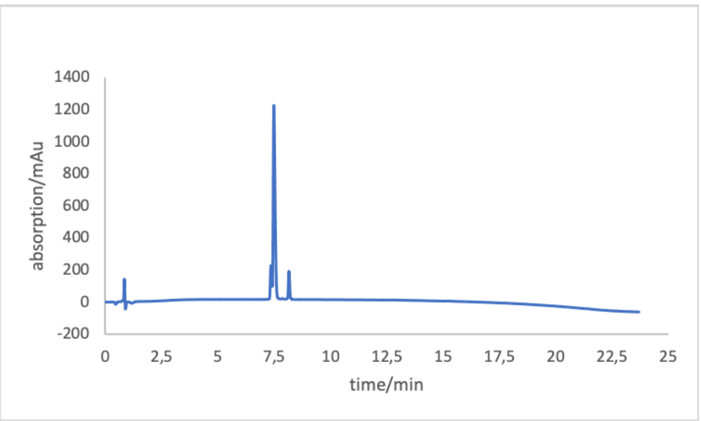

Figure S33: *rp* HPLC chromatogram of peptide **17** (0.45 mL/min, 10-90% MeCN in 20 min).

cyclo(-Ala-Leu-ehr=Pro-Leu-leu) (**11**)

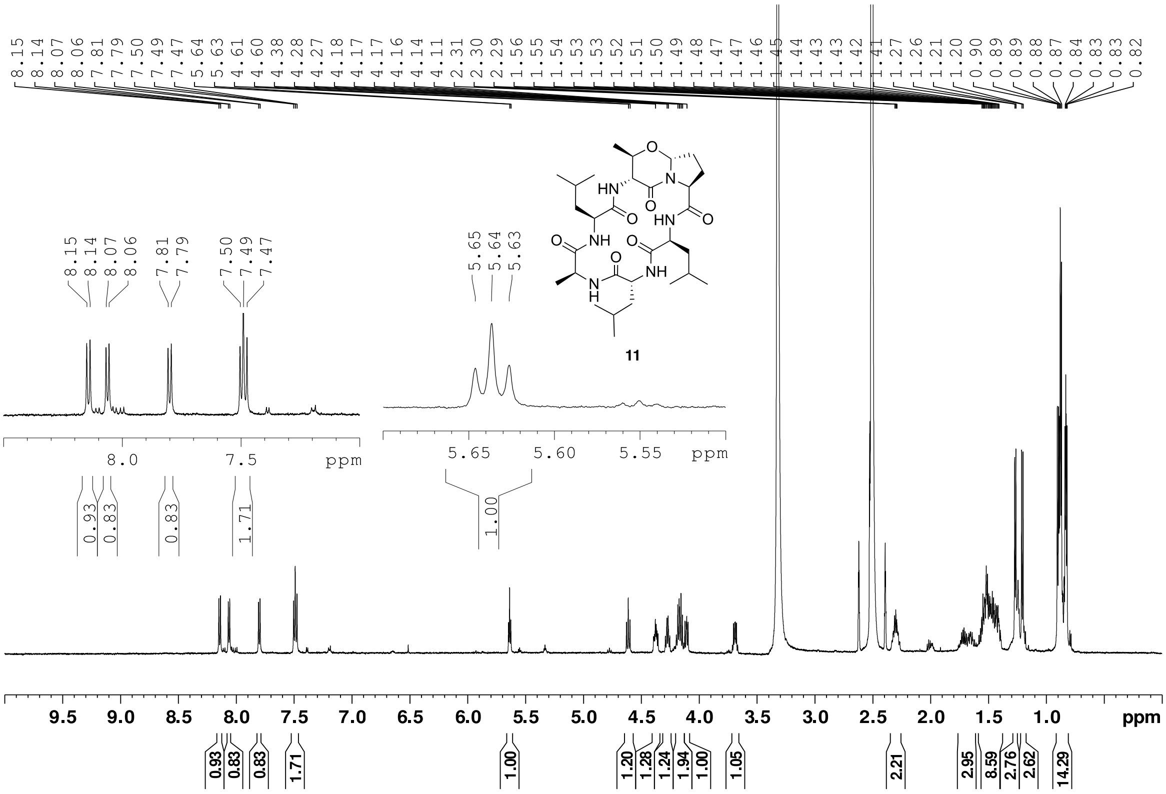

Figure 34: ^{1}H NMR spectrum of peptide **11** with enlargements of the amide region and the δH Pro (600 MHz, 300 K, DMSO-d$_6$).

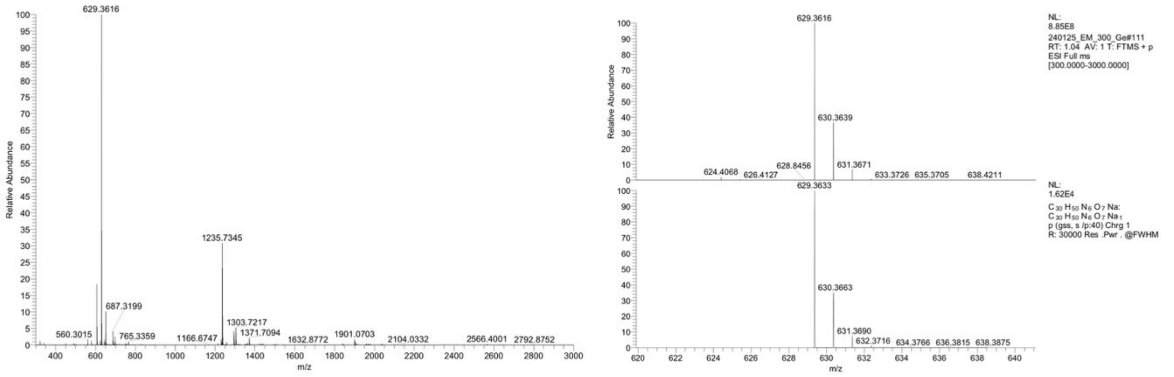

Figure S35: left: ESI+ mass spectrum of peptide **11**; right: high resolution mass spectrum of peptide **11**.

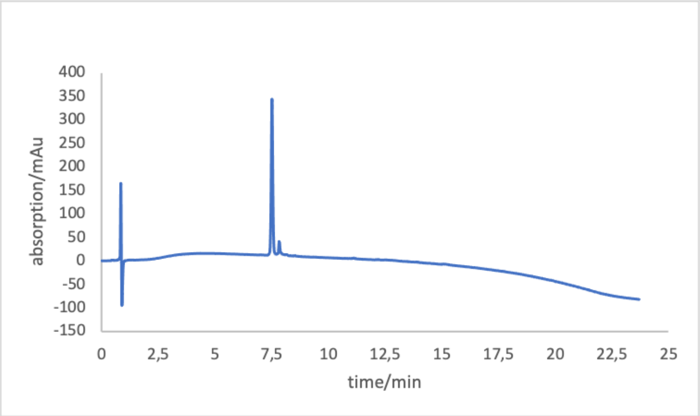

Figure S36: *rp* HPLC chromatogram of peptide **11** (0.45 mL/min, 10-90% MeCN in 20 min).

TFA.H-ehr=Pro-Phe-Gly-Gly-Gly-OH (**21**)

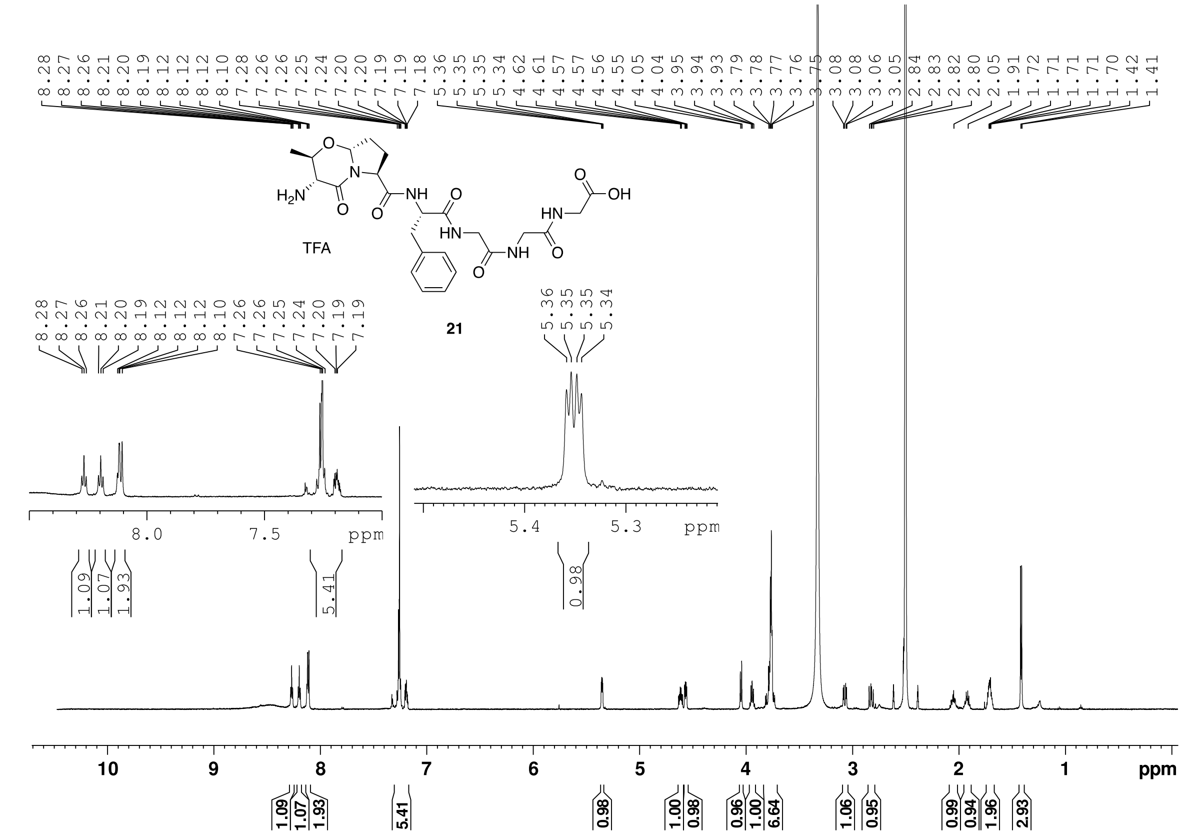

Figure S37: ^{1}H NMR spectrum of the peptide **21** with enlargements of the amide and aromatic region and the δH Pro (600 MHz, 300 K, DMSO-d_6).

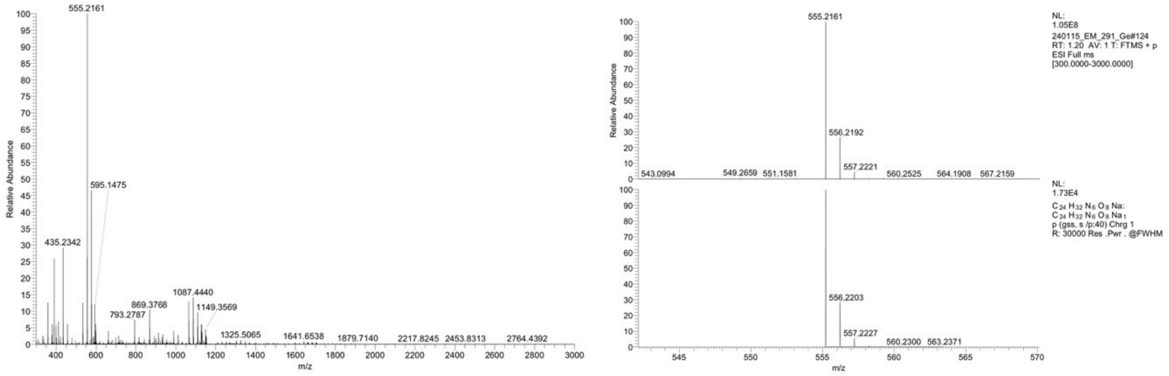

Figure S38: left: ESI+ mass spectrum of peptide **21**; right: high resolution mass spectrum of peptide **21**.

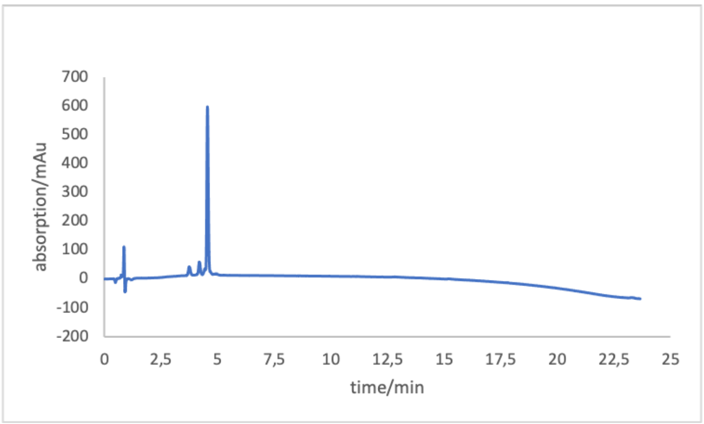

Figure S39: *rp* HPLC chromatogram of peptide **21** (0.45 mL/min, 10-90% MeCN in 20 min).

cyclo(-ehr=Pro-Phe-Gly-Gly-Gly) (**13**)

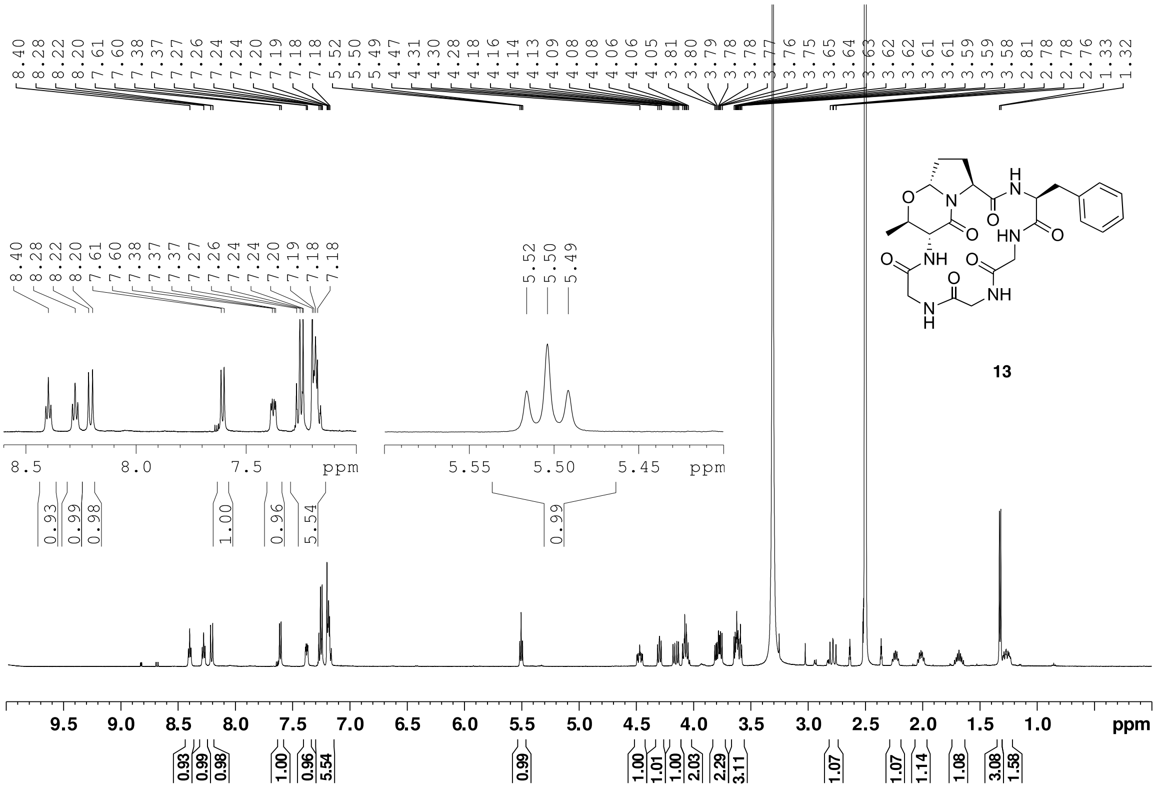

Figure 40: ^{1}H NMR spectrum of peptide **13** with enlargements of the amide and aromatic region and the δH Pro (600 MHz, 300 K, DMSO-d$_6$).

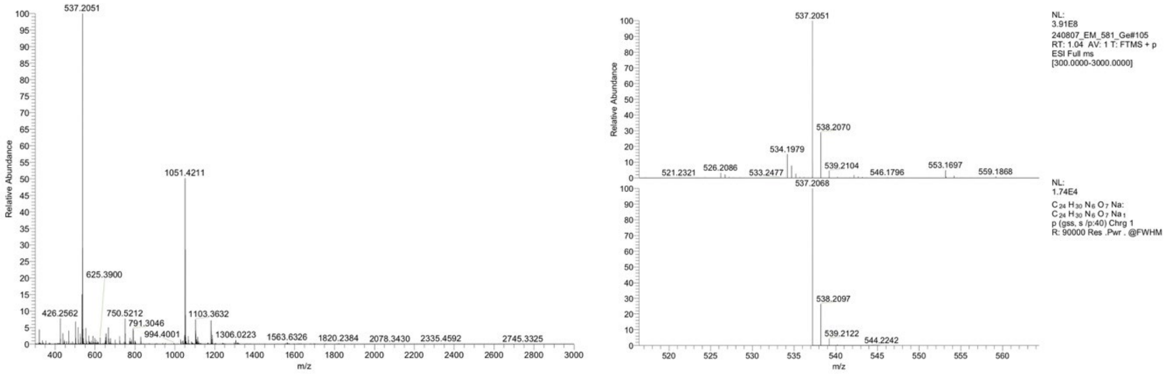

Figure S41: left: ESI+ mass spectrum of peptide **13**; right: high resolution mass spectrum of peptide **13**.

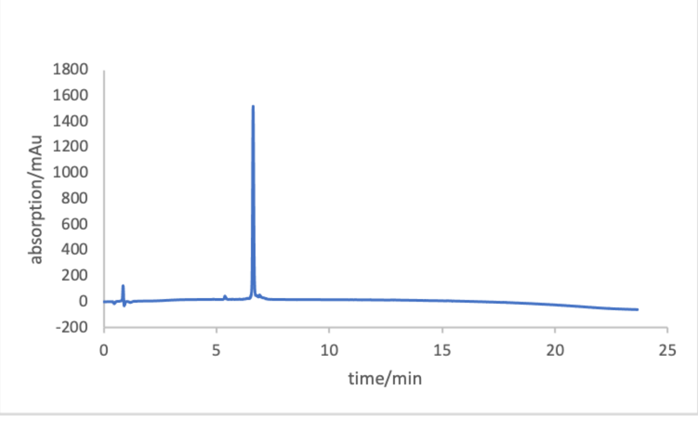

Figure S42: *rp* HPLC chromatogram of peptide **13** (0.45 mL/min, 10-90% MeCN in 20 min).

cyclo(-ehr-Hag-Phe-Gly-Gly-Gly) (**18**)

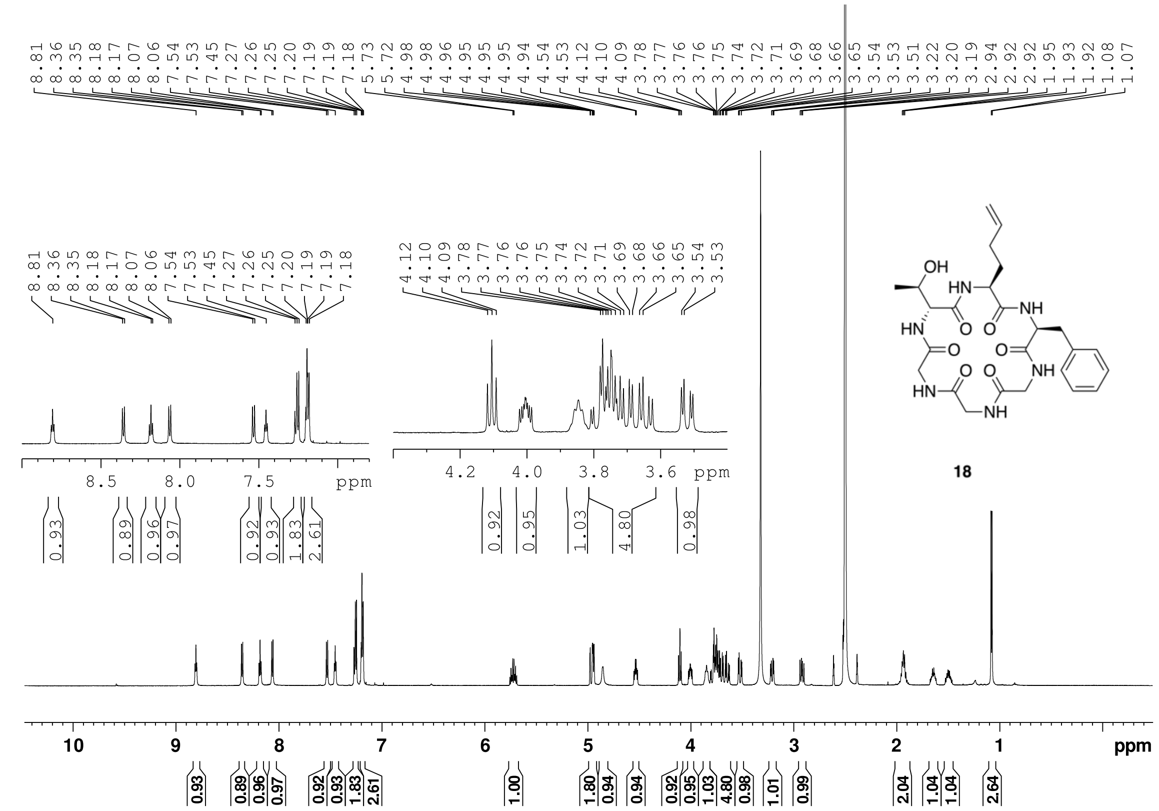

18

Figure S43: ^{1}H NMR spectrum of peptide **18** with enlargements of the amide region and the αH region (600 MHz, 300 K, DMSO-d_6).

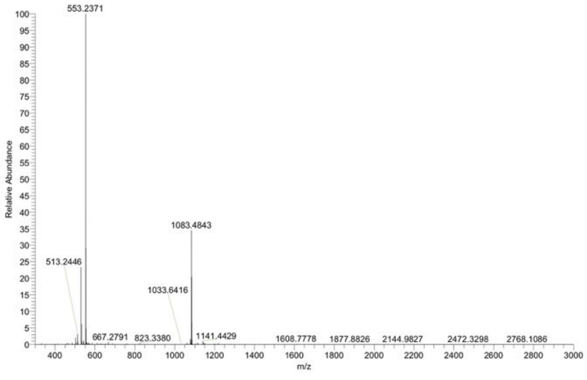

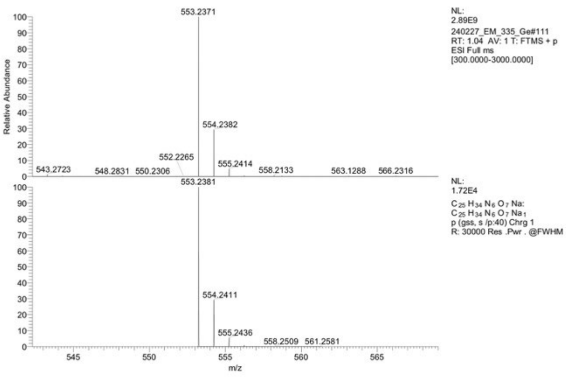

Figure S44: left: ESI+ mass spectrum of peptide **18**; right: high resolution mass spectrum of peptide **18**.

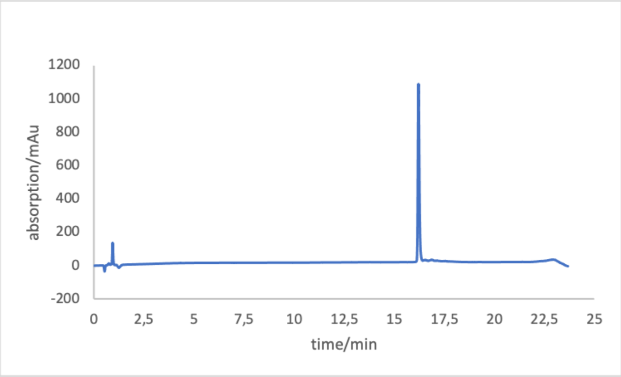

Figure S45: *rp* HPLC chromatogram of peptide **18** (0.45 mL/min, 1-30% MeCN in 20 min).

3. Temperature gradients

According to the appearence of the peptides in the main text.

TFA.H-Leu-ehr=Pro-leu-Leu-ala-OH (**15**)

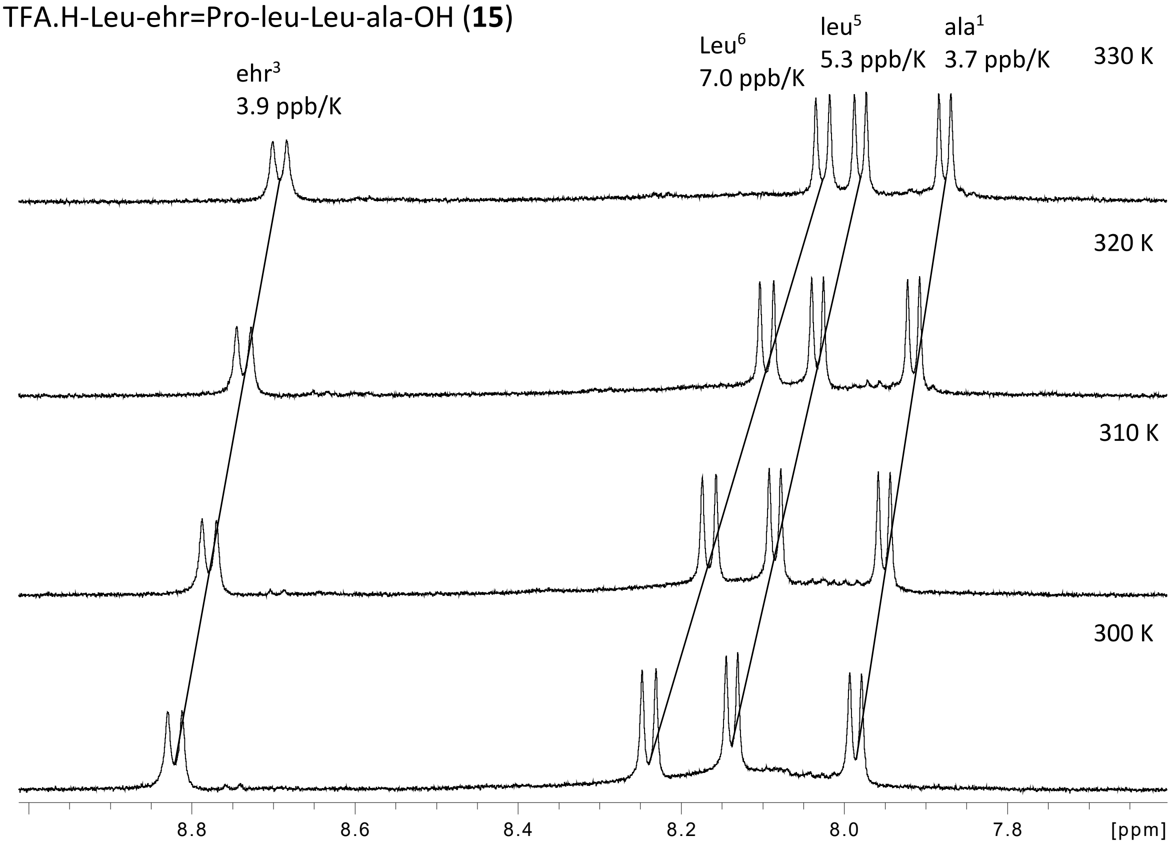

Figure S46: Amide region of ^{1}H NMR spectra at different temperatures of the peptide **15** (500 MHz, 300-330 K, DMSO-d_6).

TFA.H-Leu-ehr-Hag-leu-Leu-ala-OH (**20**)

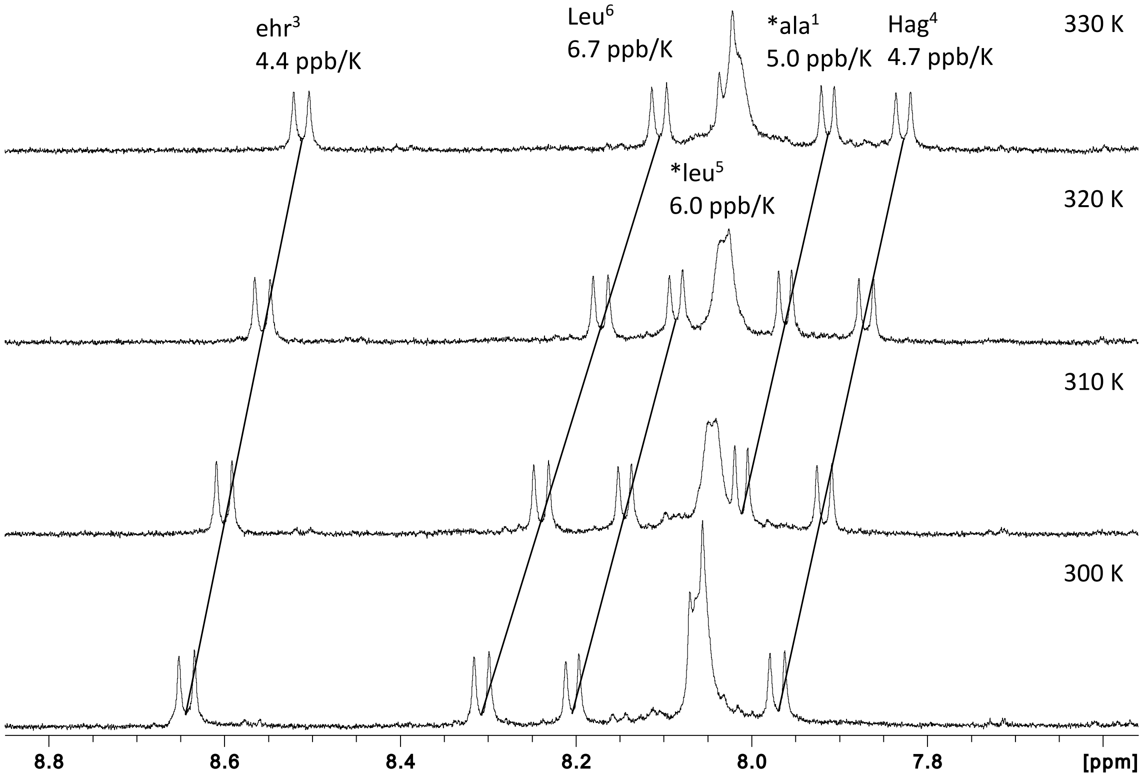

Figure S47: Amide region of ^{1}H NMR spectra at different temperatures of the peptide **20** (500 MHz, 300-330 K, DMSO-d_6). * temperature gradients were calculated only with the range of 300 K to 320 K or 310 K to 330 K.

cyclo(-ala-Leu-ehr-Hag-leu-Leu) (**12**)

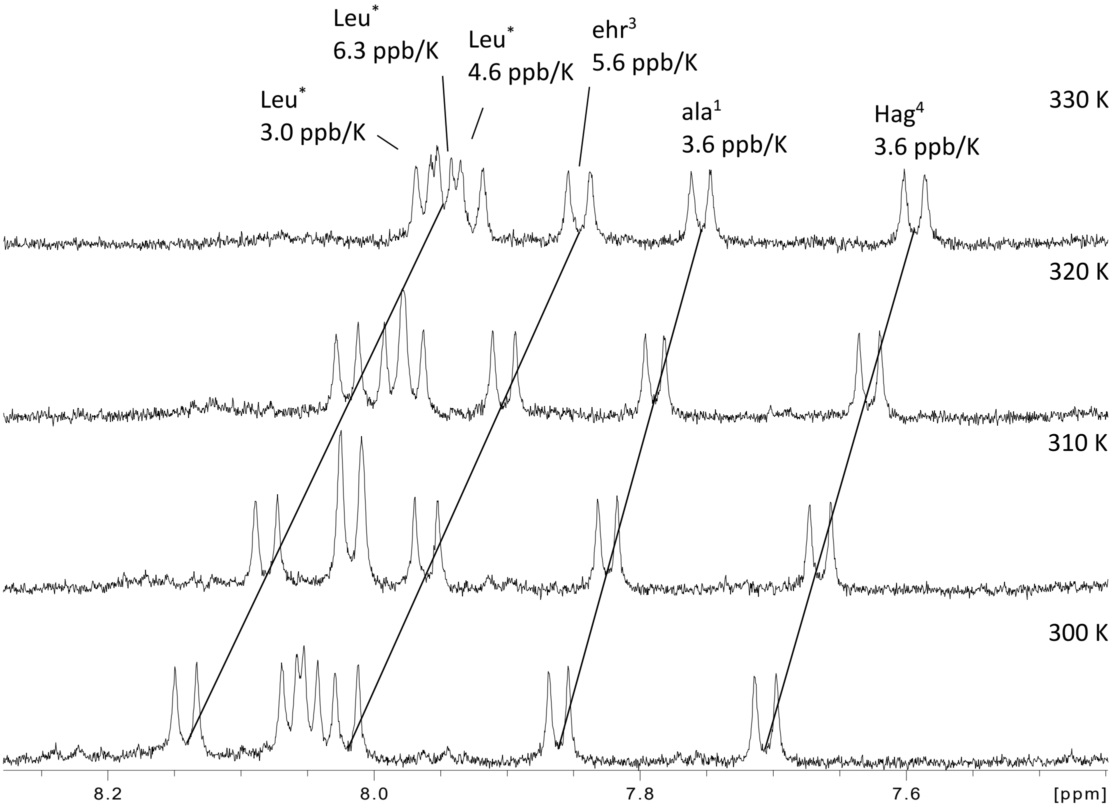

Figure S48: Amide region of ^{1}H NMR spectra at different temperatures of the peptide **12** (500 MHz, 300-330 K, DMSO-d_6). * the three leucines could not be doubtlessly differentiated and assigned.

TFA.H-Leu-ehr=Pro-Leu-Leu-Ala-OH (**16**)

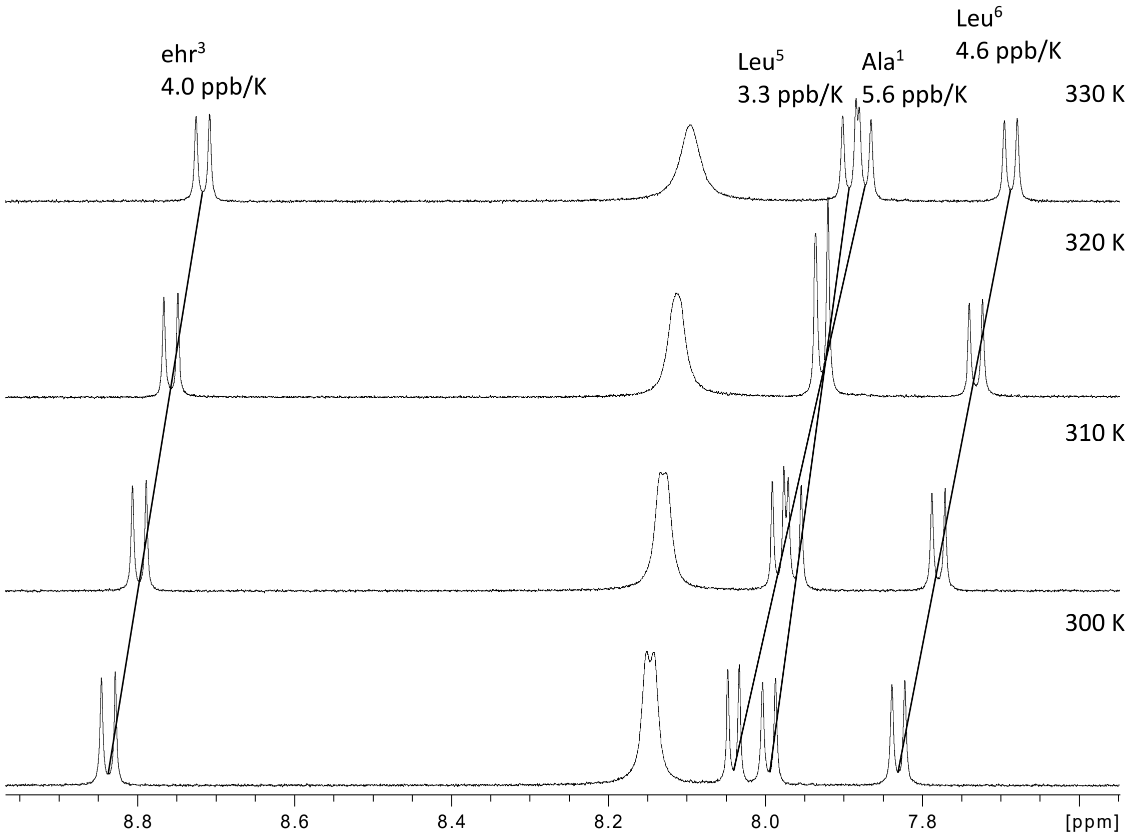

Figure S49: Amide region of ^{1}H NMR spectra at different temperatures of the peptide **16** (500 MHz, 300-330 K, DMSO-d_6).

TFA.H-Leu-ehr=Pro-Leu-leu-Ala-OH (**17**)

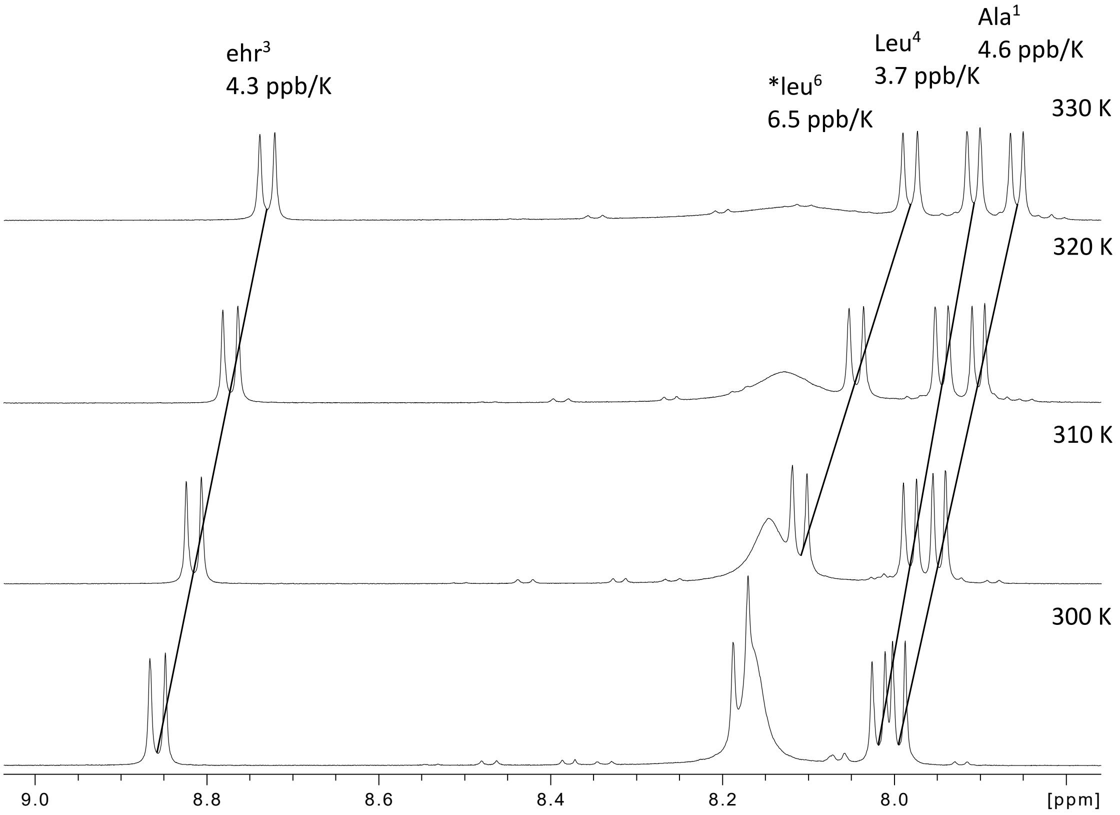

Figure S50: Amide region of ^{1}H NMR spectra at different temperatures of the peptide **17** (500 MHz, 300-330 K, DMSO-d_6). * temperature gradients were calculated only with the range of 310 K to 330 K

cyclo(-ehr=Pro-Phe-Gly-Gly-Gly) (**13**)

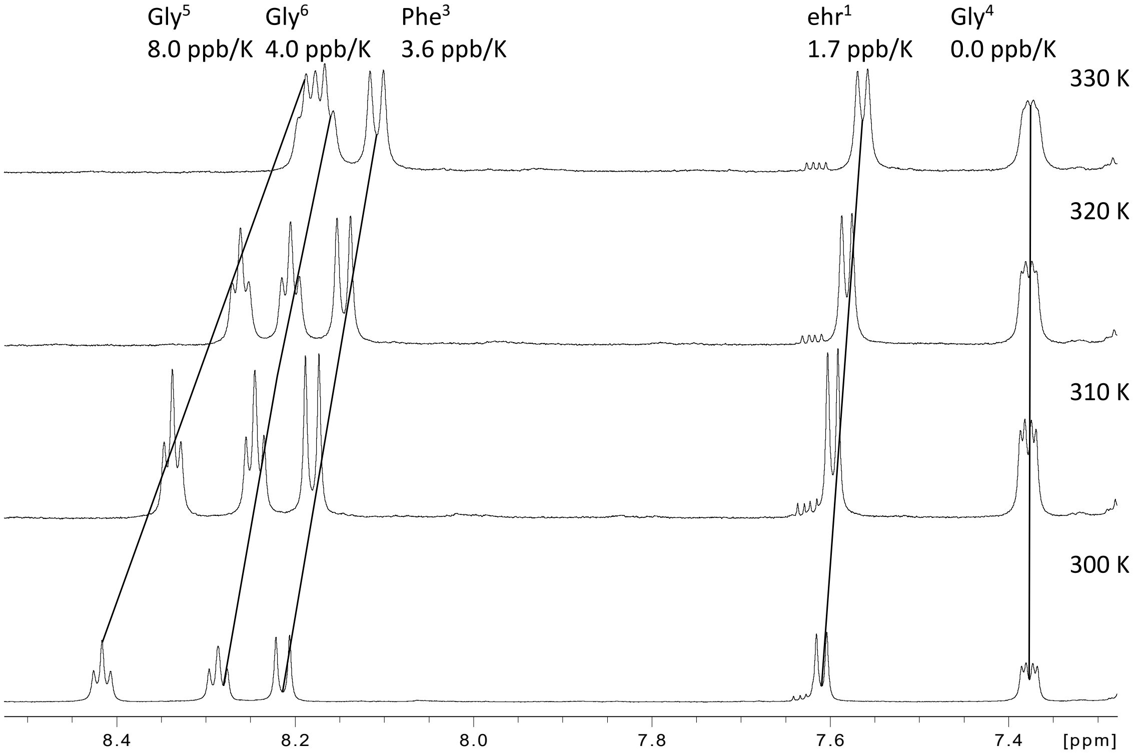

Figure S51: Amide region of ^{1}H NMR spectra at different temperatures of the peptide **13** (500 MHz, 300-330 K, DMSO-d_6).

cyclo(-ehr-Hag-Phe-Gly-Gly-Gly) (**18**)

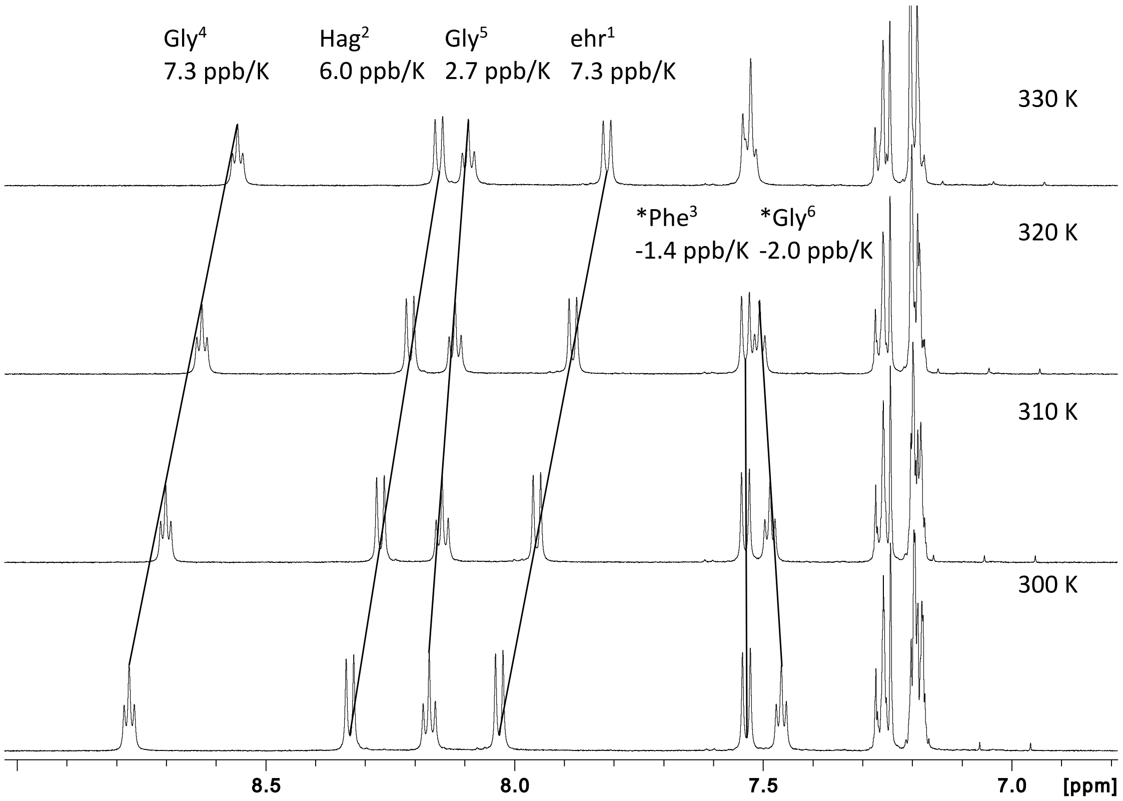

Figure S52: Amide region of ^{1}H NMR spectra at different temperatures of the peptide **18** (500 MHz, 300-330 K, DMSO-d_6). * temperature gradients were calculated only with the range of 300 K to 320 K.

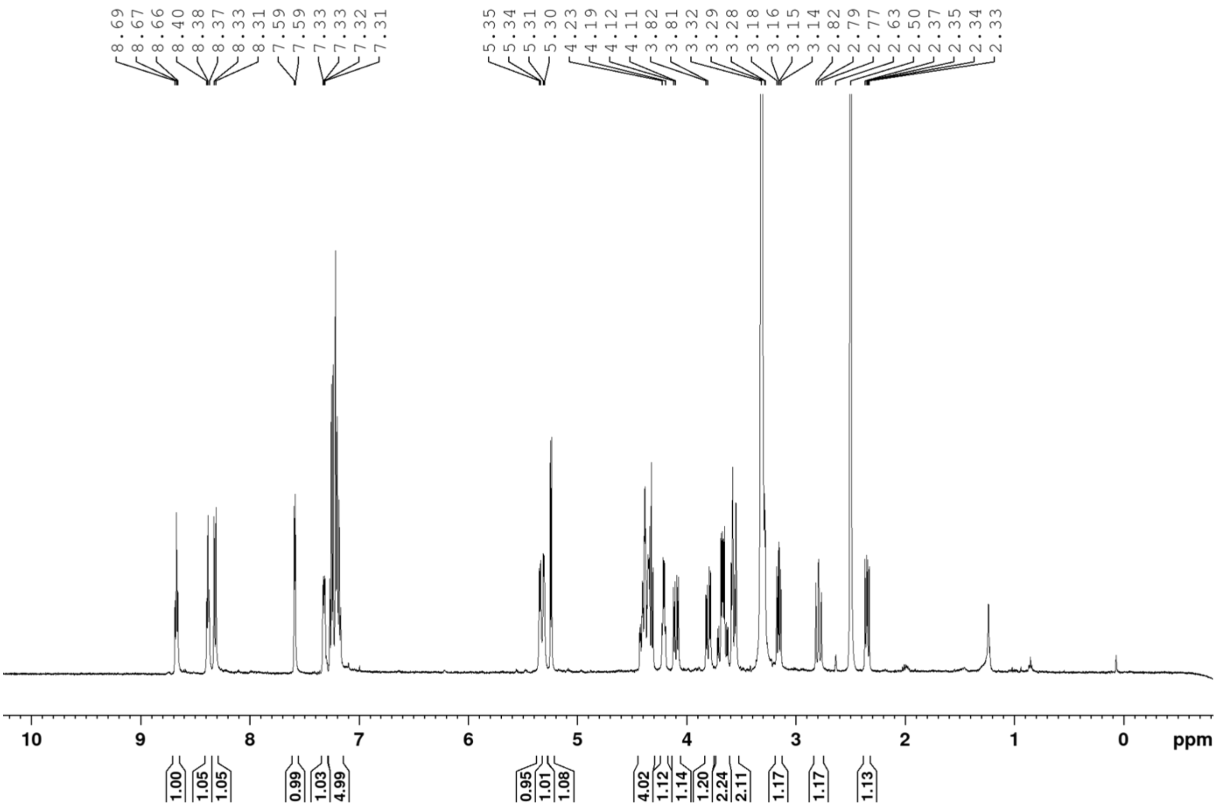

Figure S53: ^{1}H NMR spectrum of *cyclo*(-hot=Tap-Phe-Gly-Gly-Gly) (**14)** recorded by Wuttke[1] (500 MHz, 300 K, DMSO-d_6).

Table S13: Comparison of the temperature coefficients of *cyclo*(-ehr=Pro-Phe-Gly-Gly-Gly) (**13**) and *cyclo*(-hot=Tap-Phe-Gly-Gly-Gly) (**14,** taken from the literature[1])

peptide **13**	temperature coefficients	temperature coefficients	peptide **14**
ehr^1	1.7 ppb/K	2.5 ppb/K	hot^1
Pro^2	-	-	Pro^2
Phe^{3*}	3.6 ppb/K	4.0 ppb/K	Phe^{3**}
Gly^4	0.0 ppb/K	0.2 ppb/K	Gly^4
Gly^5	8.0 ppb/K	6.3 ppb/K	Gly^5
Gly^6	4.0 ppb/K	4.2 ppb/K	Gly^6

*) $^3J_{H\alpha,H\beta proS}$ = 3.7 Hz, $^3J_{H\alpha,H\beta proR}$ = 12.0 Hz; **) $^3J_{H\alpha,H\beta proS}$ = 3.5 Hz, $^3J_{H\alpha,H\beta proR}$ = 12.1 Hz

4. Late-stage Oxidation of peptide **18**

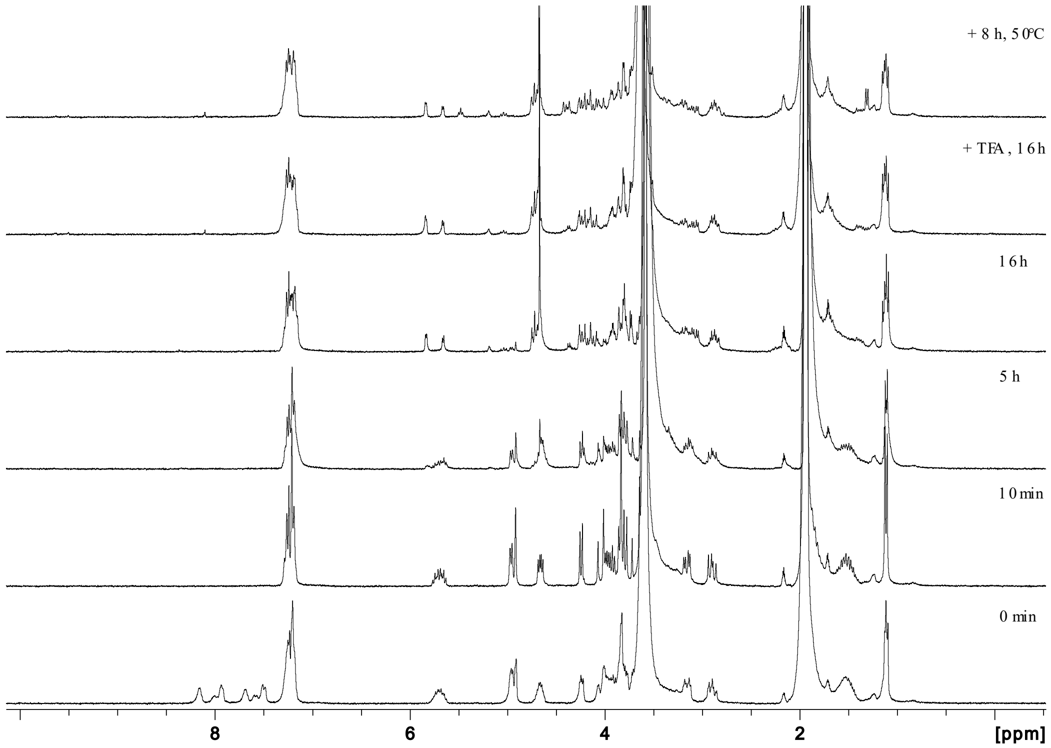

Figure S54: Complete spectra (300 MHz, 300 K, CD_3CN/D_2O 4:1) of the Lemieux-Johnson Oxidation of the peptide *cyclo*(-ehr-Hag-Phe-Gly-Gly-Gly) (**18**) to the peptide *cyclo*(-ehr=Pro-Phe-Gly-Gly-Gly) (**13**).

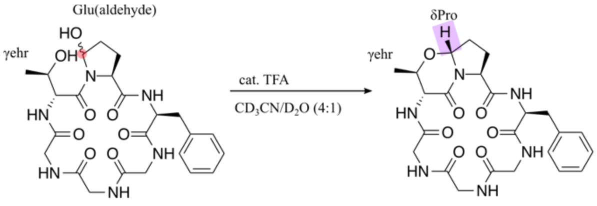

19 **13**

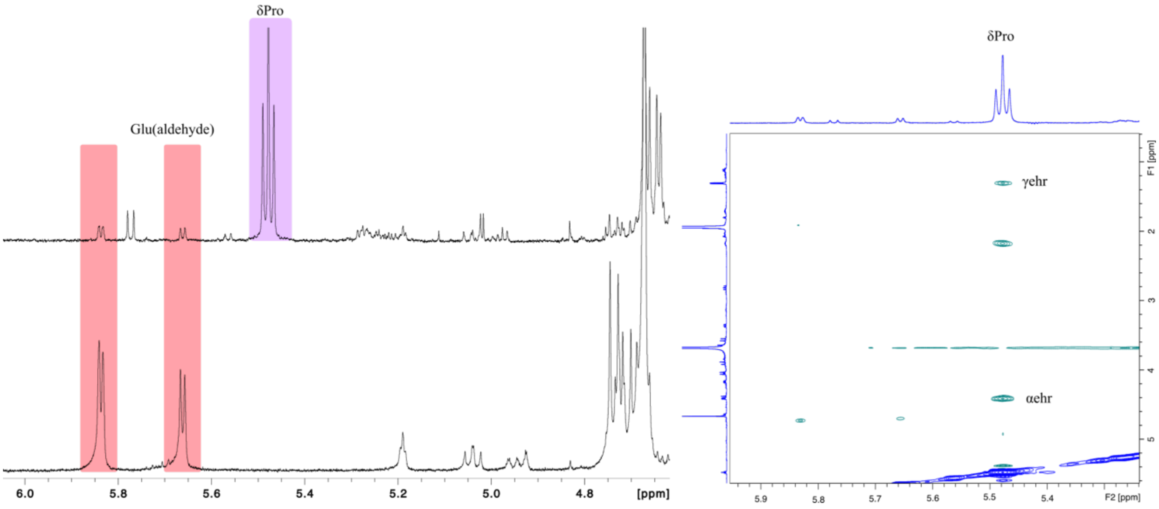

Figure S55: left: Comparison of the ^1H spectra of peptide **19** and peptide **13**, ratio 30 days after TFA addition 0.06:1 (**19**:**13**) (500 MHz, 300 K, CD_3CN/D_2O, 4:1). Right: Enlargement of the ROESY spectra of peptide **13** to determine the configuration of the bridgehead δPro.

5. Comparison of the NMR spectra

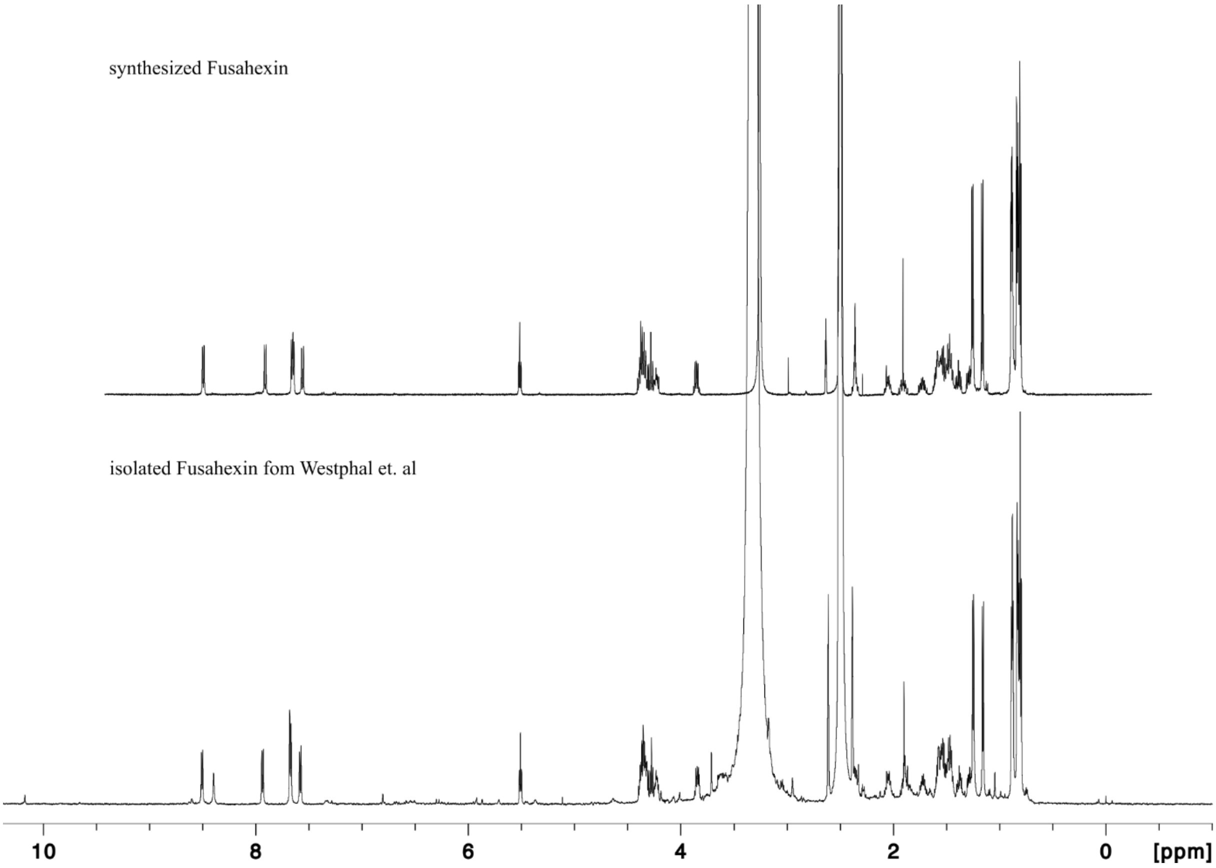

Figure S56: Comparison of the ^{1}H NMR spectra of synthetic and isolated Fusahexin. Top: Synthetic Fusahexin (**1**) (600 MHz, 310 K, DMSO-d_6). Bottom: Natural Fusahexin. Spectrum recorded by Westphal et al (600 MHz, 308 K, DMSO-d_6).[2]

6. Computer modeling

Molecular dynamics (MD) simulations were performed using YASARA[3] structure software with NOVA force field. Each protein was placed into a cubic box and the cell was filled with water molecules. Intramolecular NOEs served as restraints in the MD simulation. Interresidual NOEs were classified as strong, medium, and weak respectively and used them as restraints in the modeling. Columns lower and upper limit in the tables below describe are the deviation from the target value outside which a restoring forces starts to act. The simulation time was 100 ns (300 K) followed by energy minimization. The program Chimera was used to visualize the peptide for the figures shown in the manuscript.

Table S14: NOE and hydrogen bond restraints for Fusahexin (**1**).

residue	residue	restraint [Å]	lower limit	upper limit
Leu2 NH	ehr3 NH	3.0	0.5	0.8
leu5 NH	Leu6 NH	2.3	0.3	0.8
leu5 Hα	Leu6 NH	2.0	0.1	0.7
Leu2 Hα	ehr3 NH	2.0	0.1	0.7
Leu6 Hα	ala1 NH	2.3	0.3	0.7
Pro4 Hα	leu5 NH	2.0	0.3	0.7
ehr3 NH	Leu6 CO	2.0	0.2	0.5

Table S14: NOE and hydrogen bond restraints for peptide **11**.

Residue	residue	restraint [Å]	lower limit	upper limit
Ala1 NH	Leu2 NH	2.0	0.2	0.7
Ala1 NH	leu6 Hα	2.0	0.2	0.7
Leu2 Hα	ehr3 NH	2.0	0.1	0.7
ehr3 NH	ehr3 Hβ	2.0	0.1	0.7
ehr3 Hα	Pro4 Hδ	2.0	0.1	0.7
ehr3 Hα	Leu5 NH	3.0	0.3	0.8
Pro4 Hα	Leu5 NH	3.0	0.2	0.8
Pro4 Hδ	Leu5 NH	3.0	0.2	0.8
Leu5 Hα	leu6 NH	2.0	0.2	0.7
Leu2 NH	Leu5 CO	2.0	0.2	0.5
Leu5 NH	Leu2 CO	2.0	0.2	0.5

Table S15: NOE table for peptide **13**. NOEs are categorized in three intensities according to their cross signal hight in the ROESY spectrum (s strong, m medium, w weak).

Residue	residue	NOE
Gly6 NH	ehr1 NH	m
Phe3 NH	Gly4 NH	s
Phe3 NH	Pro2 Hδ	m
Phe3 NH	Pro2 Hα	m
Phe3 NH	Phe3 HβproR	s
ehr1 NH	ehr1 Hβ	m
ehr1 NH	Gly6 HαproS	w
ehr1 NH	Gly6 HαproR	w
Gly5 NH	Gly4 HαproS	m
Gly5 NH	Gly4 HαproR	m
Gly6 NH	Gly5 HαproS	m
Gly6 NH	Gly5 HαproR	m

7. References

[1] Wuttke A.; Synthetische Oligomere von amyloidogenen Peptiden durch reversible Chemie, Dissertation, **2016**, Philipps-University Marburg.

[2] Westphal, K. R.; Bachleitner, S.; Severinsen, M. M.; Brundtø, M. L.; Hansen, F. T.; Sørensen, T.; Wollenberg, R. D.; Lysøe, E.; Studt, L.; Sørensen, J. L.; Sondergaard, T. E.; and Wimmer R. Cyclic, Hydrophobic Hexapeptide Fusahexin Is the Product of a Nonribosomal Peptide Synthetase in *Fusarium graminearum*. *J. Nat. Prod.* **2021**, *84* (8), 2070−2080.

[3] Krieger, E.; Koraimann, G.; and Vriend G. Increasing the Precision of Comparative Models with YASARA NOVA – a Self-Parameterizing Force Field. *Proteins: Structure, Function, and Genetics*. **2002**, *47*, 393-402.
